# EthoCRED: a framework to guide reporting and evaluation of the relevance and reliability of behavioural ecotoxicity studies

**DOI:** 10.1111/brv.13154

**Published:** 2024-10-12

**Authors:** Michael G. Bertram, Marlene Ågerstrand, Eli S.J. Thoré, Joel Allen, Sigal Balshine, Jack A. Brand, Bryan W. Brooks, ZhiChao Dang, Sabine Duquesne, Alex T. Ford, Frauke Hoffmann, Henner Hollert, Stefanie Jacob, Werner Kloas, Nils Klüver, Jim Lazorchak, Mariana Ledesma, Gerd Maack, Erin L. Macartney, Jake M. Martin, Steven D. Melvin, Marcus Michelangeli, Silvia Mohr, Stephanie Padilla, Gregory Pyle, Minna Saaristo, René Sahm, Els Smit, Jeffery A. Steevens, Sanne van den Berg, Laura E. Vossen, Donald Wlodkowic, Bob B.M. Wong, Michael Ziegler, Tomas Brodin

**Affiliations:** ^1^ Department of Wildlife, Fish, and Environmental Studies Swedish University of Agricultural Sciences Skogsmarksgränd 17 Umeå 907 36 Sweden; ^2^ Department of Zoology Stockholm University Svante Arrhenius väg 18b Stockholm 114 18 Sweden; ^3^ School of Biological Sciences Monash University 25 Rainforest Walk Melbourne 3800 Australia; ^4^ Department of Environmental Science Stockholm University Svante Arrhenius väg 8c Stockholm 114 18 Sweden; ^5^ Laboratory of Adaptive Biodynamics, Research Unit of Environmental and Evolutionary Biology, Institute of Life, Earth, and Environment University of Namur Rue de Bruxelles 61 Namur 5000 Belgium; ^6^ TRANSfarm, Science, Engineering, and Technology Group KU Leuven Bijzondereweg 12 Bierbeek 3360 Belgium; ^7^ Center for Environmental Measurement and Modeling, Office of Research and Development U.S. EPA 26 Martin Luther King Drive West Cincinnati 45268 Ohio USA; ^8^ Department of Psychology, Neuroscience, & Behaviour McMaster University 1280 Main Street West Hamilton L8S 4K1 Ontario Canada; ^9^ Institute of Zoology Zoological Society of London Outer Circle, Regent's Park London NW1, 4RY UK; ^10^ Department of Environmental Science Baylor University One Bear Place #97266 Waco 76798‐7266 Texas USA; ^11^ National Institute for Public Health and the Environment (RIVM) Antonie van Leeuwenhoeklaan 9 Bilthoven 3721 MA the Netherlands; ^12^ German Environment Agency (UBA) Wörlitzer Platz 1 Dessau‐Roßlau 06844 Germany; ^13^ Institute of Marine Sciences, School of Biological Sciences University of Portsmouth Ferry Road Portsmouth PO4 9LY UK; ^14^ Department of Chemical and Product Safety The German Federal Institute for Risk Assessment (BfR) Max‐Dohrn‐Straße 8–10 Berlin 10589 Germany; ^15^ Goethe University Frankfurt Max‐von‐Laue‐Straße 13 Frankfurt am Main 60438 Germany; ^16^ Leibniz‐Institute of Freshwater Ecology and Inland Fisheries Müggelseedamm 310 Berlin 12587 Germany; ^17^ Helmholtz Centre for Environmental Research (UFZ) Permoserstraße 15 Leipzig 04318 Germany; ^18^ Swedish Chemicals Agency (KemI) Löfströms allé 5 Stockholm 172 66 Sweden; ^19^ Evolution & Ecology Research Centre, School of Biological, Earth & Environmental Sciences University of New South Wales, Biological Sciences North (D26) Sydney 2052 Australia; ^20^ Charles Perkins Centre, School of Life and Environmental Sciences The University of Sydney John Hopkins Drive Sydney 2006 Australia; ^21^ School of Life and Environmental Sciences Deakin University 75 Pigdons Road Waurn Ponds 3216 Australia; ^22^ Australian Rivers Institute, School of Environment and Science Griffith University Edmund Rice Drive Southport 4215 Australia; ^23^ School of Environment and Science Griffith University 170 Kessels Road Nathan 4111 Australia; ^24^ Center for Computational Toxicology and Exposure Office of Research and Development U.S. EPA, 109 T.W. Alexander Drive Durham 27711 North Carolina USA; ^25^ Department of Biological Sciences University of Lethbridge 4401 University Drive Lethbridge T1K 3M4 Alberta Canada; ^26^ Environment Protection Authority Victoria, EPA Science 2 Terrace Way Macleod 3085 Australia; ^27^ Department of Freshwater Ecology in Landscape Planning University of Kassel Gottschalkstraße 24 Kassel 34127 Germany; ^28^ Columbia Environmental Research Center U.S. Geological Survey (USGS) 4200 New Haven Road Columbia 65201 Missouri USA; ^29^ Wageningen University and Research P.O. Box 47 Wageningen 6700 AA the Netherlands; ^30^ Department of Anatomy, Physiology, and Biochemistry Swedish University of Agricultural Sciences Ulls väg 26 Uppsala 756 51 Sweden; ^31^ The Neurotox Lab, School of Science RMIT University 289 McKimmies Road Melbourne 3083 Australia; ^32^ Eurofins Aquatic Ecotoxicology GmbH Eutinger Strasse 24 Niefern‐Öschelbronn 75223 Germany; ^33^ Animal Physiological Ecology University of Tübingen Auf der Morgenstelle 5 Tübingen 72076 Germany

**Keywords:** behaviour, chemical regulation, data evaluation, hazard assessment, policy, pollution, population relevance, reliability evaluation, risk assessment

## Abstract

Behavioural analysis has been attracting significant attention as a broad indicator of sub‐lethal toxicity and has secured a place as an important subdiscipline in ecotoxicology. Among the most notable characteristics of behavioural research, compared to other established approaches in sub‐lethal ecotoxicology (e.g. reproductive and developmental bioassays), are the wide range of study designs being used and the diversity of endpoints considered. At the same time, environmental hazard and risk assessment, which underpins regulatory decisions to protect the environment from potentially harmful chemicals, often recommends that ecotoxicological data be produced following accepted and validated test guidelines. These guidelines typically do not address behavioural changes, meaning that these, often sensitive, effects are not represented in hazard and risk assessments. Here, we propose a new tool, the EthoCRED evaluation method, for assessing the relevance and reliability of behavioural ecotoxicity data, which considers the unique requirements and challenges encountered in this field. This method and accompanying reporting recommendations are designed to serve as an extension of the “Criteria for Reporting and Evaluating Ecotoxicity Data (CRED)” project. As such, EthoCRED can both accommodate the wide array of experimental design approaches seen in behavioural ecotoxicology, and could be readily implemented into regulatory frameworks as deemed appropriate by policy makers of different jurisdictions to allow better integration of knowledge gained from behavioural testing into environmental protection. Furthermore, through our reporting recommendations, we aim to improve the reporting of behavioural studies in the peer‐reviewed literature, and thereby increase their usefulness to inform chemical regulation.

## I. INTRODUCTION

**Fig. 1:**
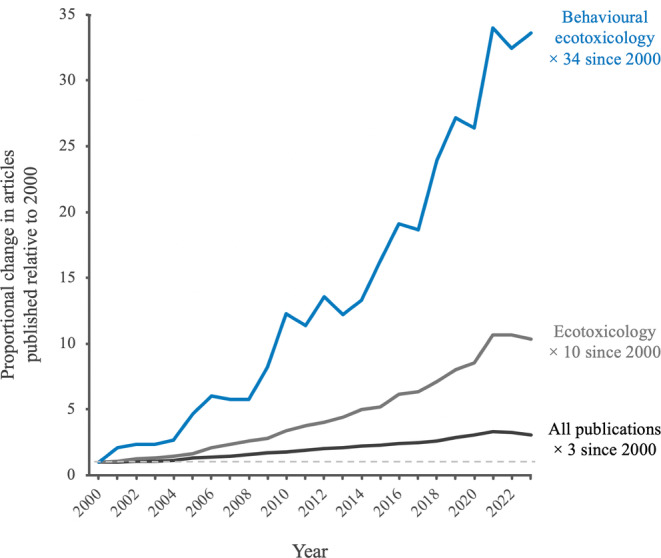
Growth in behavioural ecotoxicology literature (shown in blue; total returns: 3,684), relative to the field of ecotoxicology (shown in grey; total returns: 20,871), and publications across all research fields (shown in black; total returns: 38,143,422). Results of a *Web of Science* Core Collection search for articles published in the period between 2000 and 2023. Proportional change in yearly publications is expressed relative to the year 2000. For the full data collection method and search strings, see Appendix S1.

Behavioural analysis has become an important and widely used tool in assessing sub‐lethal toxicity. Accordingly, a substantial body of research now exists demonstrating that chemical pollutants are capable of altering animal behaviour (reviewed in Little & Finger, 1990; Clotfelter, Bell & Levering, 2004; Scott & Sloman, 2004; Zala & Penn, 2004; Gerhardt, 2007; Hellou, 2011; Melvin & Wilson, 2013; Brodin *et al*., 2014; Peterson *et al*., 2017; Pyle & Ford, 2017; Saaristo *et al*., 2018; Bertram *et al*., 2022; Porras‐Rivera, Górski & Colin, 2024). Behavioural changes have been shown across a wide array of species and as a result of contamination with a broad range of chemicals. For instance, waterborne exposure to endocrine disruptors alters reproductive behaviour and mating preferences in fish (Bertram *et al*., 2015), ingestion of polystyrene microplastics causes altered swimming activity and phototactic behaviour in daphnids (De Felice *et al*., 2019), and feeding on seeds contaminated with neonicotinoid insecticides delays migration in songbirds (Eng, Stutchbury & Morrissey, 2019). Such behavioural endpoints have drawn the attention of researchers for several key reasons. First, behaviour can be exceptionally sensitive to even low environmentally realistic contaminant exposures, and it is often disrupted at substantially lower exposure levels than conventional ecotoxicological endpoints (e.g. development, reproduction, and mortality; reviewed in Melvin & Wilson, 2013). Second, technological and methodological innovations over recent years have made behavioural ecotoxicity research more accessible and reliable than ever before (reviewed in Bertram *et al*., 2022). Third, behaviour represents the connection between an organism and its environment, meaning that a failure to generate and maintain appropriate behaviours can have adverse outcomes at both the individual and population levels (Wong & Candolin, 2015). As a result of these factors, and more, behavioural ecotoxicology research has grown rapidly over the last two decades, with the number of articles published per year increasing by a factor of 34 between 2000 and 2023 (Fig. 1; see online Supporting Information, Appendix S1, for data collection methods and search terms). Further, research in behavioural ecotoxicology is only expected to increase in the future, given that there is an ongoing shift in ecotoxicology towards sub‐lethal and environmentally realistic endpoints.

Among the most notable characteristics of behavioural ecotoxicity research compared to other established approaches in ecotoxicology are the diversity of endpoints being considered and the wide range of study designs being used (Sumpter, Donnachie & Johnson, 2014; Parker, 2015). Behavioural ecotoxicology studies consider an assortment of different responses, including evaluation of basic activity and locomotion parameters, avoidance and attraction, anxiety and anti‐predator responses, social interactions, circadian rhythmicity, learning and memory, mating and reproductive behaviours, and aggression (Bertram *et al*., 2022). Approaches used to investigate potential effects of contaminants on behavioural endpoints are similarly broad – which is to be expected, considering that behavioural ecotoxicology is a fundamentally multidisciplinary research area combining ethology, ecology, and toxicology (Gerhardt, 2007). For instance, researchers in behavioural ecotoxicology regularly use a wide variety of study species, obtain their study organisms from diverse sources, employ non‐standard exposure scenarios and durations, and perform a broad spectrum of behavioural assays. Importantly, behavioural ecologists have been using many of these experimental design elements for decades to examine interactions between organismal behaviour and environmental factors (Candolin & Wong 2012; Davies, Krebs & West, 2012). This comprehensiveness of experimental design approaches facilitates a more complete understanding of how different individuals, populations, and communities respond to pollutants across diverse contexts. Despite this, there has to date been limited uptake of behavioural data in hazard and risk assessments of chemicals (Ford *et al*., 2021).

Although there is no systematic way to assess the precise extent to which behavioural studies have been used, or have been considered for use, in hazard and risk assessments, a recent study by Ågerstrand *et al*. (2020) found just six cases in European Union chemical regulation where behavioural studies had either been employed as a key study or as supporting evidence, or were given low weight because of limited effects, reliability issues, or insufficient reporting. Ågerstrand *et al*. (2020) put forward three possible reasons for this limited use. These include a lack of promotion of behavioural endpoints in guidance documents for hazard and risk assessment of chemicals, a general low use of non‐standard studies from academia in hazard and risk assessment, and a lack of clarification of the importance of behavioural endpoints at the population level. More broadly, hazard and risk assessments are typically performed using endpoints such as mortality, developmental effects, reproductive output, and growth, and there has been an emphasis on studies performed according to internationally accepted standards, such as those developed by the Organisation for Economic Co‐operation and Development (OECD, 2023). These assessments are carried out to characterise effects, establish dose–response relationships, and to set guidance values like Predicted No Effect Concentrations (PNECs) or Environmental Quality Standards (EQS) (European Chemicals Agency, 2011b; European Commission, 2018). However, widening the scope of endpoints used in hazard and risk assessments to include non‐standard endpoints, such as behavioural traits, has the potential to reduce demands on time and resources, while being equivalently or more sensitive to exposure, and being relevant at the population level. Further, in addition to their use in hazard and risk assessments, behavioural endpoints can be valuable for other purposes, such as criteria development and toxicity testing of receiving waters and effluents. Clearly, incorporating behavioural data stands to benefit environmental protection efforts, but there is also a need for guidance on how to evaluate the relevance and reliability of behavioural ecotoxicity studies.

Here, we introduce the EthoCRED method – “Etho‐” derived from ethology, the scientific study of animal behaviour – for evaluating studies in behavioural ecotoxicology for assessment or regulatory purposes (available at ethocred.org, and in Appendix S2). The EthoCRED method provides a structured framework through which risk assessors and regulators can thoroughly, consistently, and transparently evaluate the relevance and reliability of behavioural ecotoxicology research. This method has been designed to serve as an extension of the “Criteria for Reporting and Evaluating Ecotoxicity Data (CRED)” project (Moermond *et al*., 2016), which accounts for the unique requirements and challenges encountered in research on animal behaviour. EthoCRED comprises 14 relevance criteria and 29 reliability criteria with which to evaluate behavioural ecotoxicity studies, with each criterion being accompanied by extensive guidance to support decision making. With this framework, EthoCRED is intended to accommodate the wide variety of experimental design approaches used in behavioural ecotoxicology and may be incorporated into regulatory frameworks in different jurisdictions in order to facilitate better integration of knowledge gained from behavioural studies into environmental protection. In addition, we provide reporting recommendations for researchers, comprising 72 specific aspects to consider when reporting behavioural ecotoxicity studies, with the goal of improving the reliability, reproducibility, consistency, and usefulness of peer‐reviewed behavioural data to inform assessments and chemical regulations.

## II. METHODS

The EthoCRED evaluation method for behavioural ecotoxicity studies was formulated by a group of 35 experts, having originally been conceived at a workshop organised by the German Environment Agency (UBA) and Stockholm University, titled “The behaviour of non‐target organisms after exposure to chemicals: possibilities of implementation in the regulatory process”. This group of experts includes academic researchers working across the fields of behavioural ecology, ecotoxicology, aquatic and terrestrial ecology, environmental science, chemical regulation, risk assessment, and risk management. It also includes experts from a range of governmental institutions and agencies, including the German Environment Agency (UBA), the German Federal Institute for Risk Assessment (BfR), the Swedish Chemicals Agency (KemI), the Office of Research and Development (ORD) within the U.S. Environmental Protection Agency (US EPA), the U.S. Geological Survey (USGS), the National Institute for Public Health and the Environment (RIVM, the Netherlands), and the Environment Protection Authority Victoria (EPA, Australia). When devising the EthoCRED evaluation method, the CRED method (Moermond *et al*., 2016) was used as a foundation, and was chosen since it is already recommended for use in the EU Water Framework Directive (European Commission, 2018). Certain CRED evaluation criteria remained unchanged (i.e. have no behaviour‐specific guidance), while others were modified to fit the specific characteristics of behavioural studies, with additional behaviour‐specific criteria and reporting recommendations also being added.

The EthoCRED manual for practical use of the relevance and reliability criteria is available at ethocred.org, as well as in Appendix S2. In the manual, each of the EthoCRED relevance and reliability criteria are accompanied by comprehensive guidance material, as well as the corresponding original CRED criteria (where relevant). Further, the EthoCRED manual details how to assign relevance and reliability categories to behavioural studies, and how to combine these criteria to generate an overall assessment of suitability for a particular risk assessment or regulatory purpose. In addition, the EthoCRED reporting recommendations are listed in the manual, to guide behavioural ecotoxicity researchers in designing and reporting their research. A separate spreadsheet containing the relevance and reliability criteria was also created to facilitate the use of the EthoCRED evaluation method by risk assessors and authors (Appendix S3). Specifically, this spreadsheet allows evaluators straightforwardly to document their choices and the rationale behind them, and to highlight uncertainties. This approach to documenting the implementation of EthoCRED will allow more seamless sharing of information among, for example, risk assessors, experts, and regulatory bodies. Further, an additional reporting recommendations spreadsheet (Appendix S4) allows researchers systematically to confirm inclusion of important details about their study that will strengthen their article.

## III. ETHOCRED EVALUATION METHOD

### (1) Relevance and reliability

Relevance is defined as *the extent to which data and tests are appropriate for a particular hazard identification or risk characterisation* (European Chemicals Agency, 2011a, p. 1). Reliability is defined as *the inherent quality of a test report or publication relating to preferably standardised methodology and the way the experimental procedure and results are described to give evidence of the clarity and plausibility of the findings* (European Chemicals Agency, 2011a, p. 1). These definitions make clear that relevance is contingent upon the specific purpose of the assessment and relates to how the study will be applied for a particular objective. Meanwhile, reliability concerns the inherent scientific quality of a study, irrespective of its intended purpose for evaluation. Consequently, a study deemed reliable might possess high relevance for one assessment but low relevance for another.

When conducting a survey of the behavioural ecotoxicity literature, a preliminary evaluation of each study's relevance can be conducted based on the title and abstract. For instance, terrestrial ecotoxicity studies might be disregarded when conducting an aquatic assessment. An evaluation of study relevance using the EthoCRED evaluation method is primarily intended for a more comprehensive analysis, carried out after the initial study selection. The determination of reliability hinges upon the assessment of the study's design, execution, and analysis. For instance, a study could be viewed as less reliable due to inadequacies in experimental design (such as insufficient replicates), subpar execution (e.g. excessive mortality in control groups), or deficient data analysis (e.g. inadequate statistical methods). A behavioural ecotoxicity study might yield multiple outcomes (e.g. activity levels, reproductive behaviour, physiological and/or morphological parameters) that are observed across different exposure scenarios. Within the confines of a single study, certain outcomes might possess relevance and/or reliability, while others may not. Furthermore, a study possessing lower relevance and/or reliability might still find utility as corroborative evidence in regulatory risk assessments, contingent upon the rationale behind the reduction in relevance/reliability.

The process by which the EthoCRED method is used to assess the potential adequacy of behavioural ecotoxicity studies to inform assessment and regulatory activities includes both an evaluation of study relevance and an evaluation of study reliability (Fig. S1). These two assessments are then combined, generating an overall evaluation of study adequacy for a specific assessment purpose – according to guidance of the European Chemicals Agency (2011a) – which is routinely linked to protection goals. Note that the relevance and reliability assessment need not be performed in a particular sequential order. However, it may be more efficient to start with a relevance assessment, because a reliability assessment of non‐relevant studies is often redundant.

### (2) EthoCRED relevance evaluation

Relevance concerns the intended application or the regulatory context for which the research is being evaluated. As a result, the degree of relevance can vary based on the specific purpose of the study. For example, a terrestrial toxicity study might not have relevance when deriving aquatic EQSs or PNECs, but it could be relevant when conducting a risk assessment related to terrestrial ecosystems. This highlights the fact that evaluating the various aspects of relevance often requires a clear understanding of the framework and objectives guiding the risk assessment process.

The EthoCRED evaluation method uses four relevance categories: (*i*) relevant without restrictions, (*ii*) relevant with restrictions, (*iii*) not relevant, and (*iv*) not assignable. A description of these categories is provided in Table 1.

**Table 1:** EthoCRED relevance categories. Note that these categories correspond with the original Criteria for Reporting and Evaluating Ecotoxicity Data (CRED) relevance categories outlined by Moermond *et al.* (2016).

Relevance category	Description
Relevant without restrictions	The study is relevant for the purpose for which it is evaluated.
Relevant with restrictions	The study has limited relevance for the purpose for which it is evaluated.
Not relevant	The study is not relevant for the purpose for which it is evaluated.
Not assignable	Studies that do not give sufficient details since the result is presented in abstracts or secondary literature (books, reviews, etc.) or studies for which the documentation is not sufficient for assessment of relevance for one or more vital parameters.

Using the EthoCRED method, relevance is evaluated based on 14 relevance criteria distributed across three categories: general information, biological relevance, and exposure relevance (Table 2). These criteria have been adapted from the CRED evaluation method (Moermond *et al*., 2016). Qualitative assessment is used to determine the degree of fulfilment of each criterion. In cases where expert judgement may be required, this has been indicated in the explanatory text accompanying each criterion. Although primarily intended for screening studies from the literature, the EthoCRED relevance criteria can also be used as a list of considerations for designing studies that are more likely to be useful for assessment and regulatory activities.

**Table 2:** EthoCRED relevance criteria^a^ for evaluating behavioural ecotoxicity data. The criteria are adapted from the relevance criteria provided in the Criteria for Reporting and Evaluating Ecotoxicity Data (CRED) project (Moermond *et al.*, 2016). Additional guidance on how to interpret the EthoCRED relevance criteria is provided in the main text.

Number	Criterion
General information	
	Before evaluating the test for relevance, indicate the reason for evaluating this study. The relevance of the study might be different for different purposes (e.g. environmental quality criteria derivation, PBT assessment, dossier evaluation for marketing authorisation), also depending on the framework for which the evaluation is requested.
Biological relevance	
1	Is the species tested relevant for the compartment (e.g. soil, water) under evaluation?
2	Are the organisms tested relevant for the tested compound?
3	Are the reported endpoints appropriate for the intended application or potential regulatory purposes?
4^†^	Are the behaviours quantified relevant for the study species?
5^†^	Are the behaviour‐testing arena(s) used relevant to the tested species and the endpoints quantified?
6	Are the reported endpoints appropriate for the investigated effects or the mode of action of the test substance?
7	Is the effect relevant on a population level?
8	Is the magnitude of effect statistically meaningful and biologically relevant for the intended application or potential regulatory purposes (e.g. EC_10_, EC_50_)?
9	Are relevant life stages studied?
10	Are the experimental conditions relevant for the tested species?
11	If recovery is studied, is this relevant for the framework for which the study is evaluated?
Exposure relevance	
12	Is the tested exposure scenario relevant for the substance?
13	Is the exposure duration relevant and appropriate for the studied species and endpoints?
14	In case of a formulation, other mixture, salts, or transformation products, is the substance tested representative and relevant for the substance being assessed?

^†^ Criteria that specifically relate to behavioural ecotoxicity studies, which are additional to the original CRED criteria. Note that most criteria are not *per se* critical for the relevance of a study and that this depends strongly on the purpose of the evaluation.

^a^ See main text for further explanation of the EthoCRED criteria and explanatory guidance text on how to interpret the criteria.

CRED, Criteria for Reporting and Evaluating Ecotoxicity Data; EC_10_/EC_50_, 10% and 50% effective concentrations; PBT, persistent, bioaccumulative, and toxic.

### (3) Explanation of the EthoCRED relevance criteria (criterion numbers from Table 2)

Relevance criteria with EthoCRED‐specific guidance are reported here. For the full list of criteria, including those that do not differ from the original CRED relevance criteria (Moermond *et al*., 2016), see Appendix S2.

#### (a) EthoCRED relevance criterion #2: are the organisms tested relevant for the tested compound?

In behavioural ecotoxicology, it is common that not only standard model species (e.g. zebrafish, *Danio rerio*; African clawed frog, *Xenopus laevis*) but also non‐model organisms are studied. Generally, both model and non‐model species can be relevant, although studies should ideally provide a sensible rationale for the choice of species with respect to the goal of the research – for example a species that is likely to be exposed in the wild, a keystone species, a particularly sensitive/robust species, and/or a suitable/convenient model species to predict impacts in other animals. It is important to note that, particularly for popular model species, various strains may be available that differ in genetic composition (Suurväli *et al*., 2020). This includes laboratory strains (which may or may not be genetically uniform or inbred) and wild strains (which are often, but not always, more genetically diverse), and the relevance of the strain(s) used should be evaluated considering the goals of the study. For instance, genetically impoverished laboratory strains may be relevant when high levels of standardisation (and limited among‐individual variation) are needed. However, different strains could also differ in their sensitivity and responses, making it difficult to generalise beyond the strain being tested (Aulsebrook, Wong & Hall, 2022). Also, strong selection for optimal performance under laboratory conditions means that behavioural responses of laboratory animals may no longer accurately reflect those of their wild counterparts (e.g. Morgan *et al*., 2022), and, under those circumstances, non‐domesticated strains/populations will often be more suitable for studies that are aimed at predicting behavioural responses of wild populations (Thoré *et al*., 2021c). In the latter case, it becomes important to be mindful that wild populations often experience different evolutionary trajectories (e.g. populations from non‐pristine environments may already be adapted to the chemical under investigation), which may lead to differences in how wild animals respond to chemical exposure (Almeida *et al*., 2021; Brans, Almeida & Fajgenblat, 2021). Therefore, in order to understand species‐level responses, it may be necessary to test across multiple strains or populations (see also EthoCRED reliability criterion #9, Section III.5.f).

Beyond the choice of species and strain/population, other characteristics may also determine the relevance of the study organisms for the tested compound. For instance, males and females often differ in their behavioural baseline (Thoré *et al*., 2019a), their behavioural response to chemical exposure (Bertram *et al*., 2015; Vossen *et al*., 2022), or both (Martin *et al*., 2019a). Therefore, studies that do not account for potential differences between sexes may be less relevant, unless justification can be provided – for example when it is impossible to sex individuals, as is often the case for juvenile life stages and sexually monomorphic species, when the compound is not expected to have sex‐specific effects, and/or when previous research has demonstrated no difference in behavioural baseline between sexes. Besides sex, age or life stage may also determine the behavioural baseline and behavioural effects of chemical exposure and should be justified (see also EthoCRED relevance criterion #9, Section III.3.h).

#### (b) EthoCRED relevance criterion #3: are the reported endpoints appropriate for the intended application or potential regulatory purposes?

In conventional ecotoxicology, apical endpoints such as survival, growth, and reproduction are typically used for assessment or regulatory purposes. In behavioural ecotoxicology, there is a whole range of behavioural traits that are quantifiable, sensitive to chemical exposure, and directly or indirectly linked to traditional apical endpoints and the fitness of animals. For example, a fish swimming erratically at the surface and struggling to maintain its upright position in the water column may be more vulnerable to predation, animals that show impaired courtship and mating behaviours may have lower reproductive success, and animals with reduced mobility may not be able to acquire adequate nutrition, leading to impaired growth and/or survival. Usually, behaviours/behavioural responses are classified under one of five interrelated categories: activity (e.g. activity level, swimming velocity), boldness/shyness/anxiety (e.g. thigmotaxis or wall‐hugging behaviour, light–dark preference or scototaxis, gravity‐mediated activity or geotaxis), exploration behaviour (e.g. inspection of a novel environment), aggressiveness (e.g. association time with a mirror image), and sociability (e.g. shoaling tendency, group cohesion).

It is important to note that behavioural expression is often driven by various concurrent motivational, cognitive, and emotional mechanisms (Budaev & Brown, 2011), so that classification of behavioural traits may be somewhat arbitrary. In addition, some behaviours are difficult to place within one of the five above‐mentioned categories but are nevertheless directly relevant for the fitness of animals, including but not limited to foraging behaviour (e.g. location of food resources, food consumption), antipredator behaviour (e.g. escape, avoidance, vigilance), and reproductive behaviour (e.g. mate choice, courtship, mating, parental care). Therefore, more important than the classification of behaviours, a sensible rationale should be provided as to why the endpoint is meaningful and whether the observed effect sizes are likely to be biologically/ecologically relevant, particularly when researchers investigate behaviours that are not commonly considered. When such justification is missing, expert judgement, informed by information on evolutionary conservation of targets and pathways of relevance to the behavioural measures, is needed to decide the appropriateness of the endpoint and the relevance of the effect size (see also EthoCRED relevance criterion #8, Section III.3.g).

#### (c) EthoCRED relevance criterion #4: are the behaviours quantified relevant for the study species?

Measuring the potential effect of exposure to a chemical on an animal's behaviour does not necessarily mean that the observed effect is relevant to the species under investigation. In this regard, a properly designed study to investigate the possible effects of chemicals on animal behaviour must consider the normal behavioural repertoire of the species. For example, evaluating total distance travelled in a sedentary animal may be less relevant to that species' survival than behaviours that do not require travelling significant distances, such as mandible rolling or tail flicks. Care should also be taken when translating a metric used in one species (e.g. diving response in zebrafish) to other species. Consequently, a study is only relevant when the biology and ecology of the studied species are properly factored into the design of the study (see also EthoCRED relevance criterion #10, Section III.3.i) and the behavioural test(s), which should ideally be motivated (at least in the case of species that are not commonly studied) to aid expert evaluation.

#### (d) EthoCRED relevance criterion #5: are the behaviour‐testing arena(s) used relevant to the tested species and the endpoints quantified?

Careful consideration must be given to the design of the behaviour‐testing arena and its relevance to the species and endpoints under investigation. This includes accommodating the basic physiological requirements of the species – which will, in part, be determined by size and life stage – and its natural behavioural tendencies (see also EthoCRED relevance criterion #4, Section III.3.c). These design considerations include, among others, the dimensions of the arena, temperature, photoperiod, and flow regime (in the case of wind tunnels and water flumes). The importance and impact of each of these factors is largely species‐ and life‐stage specific (see also EthoCRED reliability criterion #17, Section III.5.k). For instance, testing arenas that are either too small or too large may not allow a species to display its natural behavioural repertoire (e.g. restricted activity of large animals when the arena is too small, unanticipated fright responses of cryptic or prey animals when the arena is too large). In addition to its size, the shape of the arena must also be appropriate for the species and behavioural endpoint under investigation. For example, a vertical column is more relevant when investigating diel vertical migrations of *Daphnia* species than a shallow rectangular aquarium (Kohler, Parker & Ford, 2018).

Behavioural assessment should be conducted at ambient conditions that are relevant for the tested species and that promote the expression of normal behaviour. For instance, temperature can influence a wide range of behaviours (e.g. several species only display mating behaviour at specific temperatures, such as burbots *Lota lota*, which only spawn when temperatures fall below 4 °C; McPhail & Paragamian, 2000). Also, animals should be tested in a flow regime (i.e. still *versus* moving) that matches the species' natural habitat. For instance, testing a pelagic fish in a fast‐moving water flume is less relevant than using a static open‐field arena, or *vice versa* for a riverine fish. Similarly, if a study assesses nocturnal behaviours under brightly lit conditions instead of infrared lights, the results may not be relevant.

#### (e) EthoCRED relevance criterion #6: are the reported endpoints appropriate for the investigated effects or the mode of action of the test substance?

Fundamentally the same as CRED, but specifically related to animal behaviour. For example, endocrine‐disrupting chemicals that mimic reproductive hormones are most likely to affect reproductive behaviours (reviewed in Söffker & Tyler, 2012; Gore, Holley & Crews, 2018), and anxiolytics may cause prey animals to be excessively bold in the presence of predators (see Brodin *et al*., 2013, 2014). Importantly, however, due to the complex mechanistic underpinnings of organismal behaviour, there is also a danger of discounting the potential impact of any given contaminant on a seemingly unrelated behavioural endpoint. For instance, using the two example contaminants above, endocrine‐disrupting chemicals have also been shown to alter anxiety‐related and anti‐predator behaviours (e.g. Reyhanian *et al*., 2011; Lagesson *et al*., 2019), while anxiolytics can disrupt mating and reproductive behaviours (e.g. Bertram *et al*., 2018a; Fursdon *et al*., 2019). For this reason, it is most important that proper justification is provided for the investigated contaminant(s) and the behavioural endpoint(s) reported in a study.

#### (f) EthoCRED relevance criterion #7: is the effect relevant on a population level?

Organismal behaviour can have profound population‐level consequences through effects on key demographic parameters, such as births, deaths, and migration (Wong & Candolin, 2015; Saaristo *et al*., 2018). For example, a broad range of reproductive behaviours (e.g. courtship intensity, sexual responsiveness, mating frequency) can directly impact mating outcomes, which, in turn, can affect both the number and quality of offspring that are produced and recruited into the population (Candolin & Wong, 2019; Aulsebrook *et al*., 2020). Similarly, in species with parental care, the amount of effort invested into offspring (e.g. nest defence, provisioning rates) can also be important (Royle, Smiseth & Kölliker, 2012; Aulsebrook *et al*., 2020). Likewise, behaviours that affect how well animals are able to acquire resources (e.g. time taken to find food, feeding rates) or respond to predators (e.g. time spent hiding, activity levels) can influence population dynamics through effects on survival (Saaristo *et al*., 2018).

Most studies in behavioural ecotoxicology involve investigating the behavioural responses of animals in the laboratory, with comparatively fewer studies performed under semi‐natural or natural field conditions. When evaluating the relevance of behavioural responses at a population level, it is important to consider both the behavioural endpoint being targeted and the experimental settings in which it is being investigated. For example, standardised laboratory assays, whilst certainly valuable, are sometimes criticised for lacking ecological relevance (e.g. exposing animals to chemicals at concentrations that are several orders of magnitude higher than what is encountered in nature; not accounting for species interactions; lack of variation in natural environmental conditions; Bertram *et al*., 2022). Another important consideration is whether behavioural effects observed under laboratory conditions are predictive of how animals will respond in the wild (Saaristo *et al*., 2018). This can be tested by embracing a more integrative approach, involving research performed across multiple scales and levels of ecological complexity (e.g. testing migration of salmon smolts exposed to pharmaceutical pollution both in the laboratory and in a natural river system: Hellström *et al*., 2016), and aided by an increasingly sophisticated array of experimental tools and technological advances [e.g. high‐throughput wildlife tracking systems (Bertram *et al*., 2022; Nathan *et al*., 2022)].

#### (g) EthoCRED relevance criterion #8: is the magnitude of effect statistically meaningful and biologically relevant for the intended application or potential regulatory purposes (e.g. EC_10_ , EC_50_ )?

Statistical significance provides a degree of confidence that research findings are supported by the observed data and not due to chance. As such, it can be useful to consider any guidance provided regarding jurisdictional regulatory policy when designing experiments, choosing statistical approaches, and assessing statistical relevance. In behavioural ecotoxicology, statistical significance is important because it is widely recognised that there is considerable behavioural variation both within individuals over time and across individuals (Shaw, 2020). Among other things, the capacity to detect effects of different magnitudes relies on the sample size. In this regard, sample size is one contributing factor determining the probability of rejecting a null hypothesis of no difference between populations when they do not actually differ (i.e. type‐I error, a “false‐positive”), or failing to reject a null hypothesis that is actually false in a population (i.e. type‐II error, a “false‐negative”) (Quinn & Keough, 2002). In other words, sample size is a major determinant of statistical power, which is a measure of the probability that a study will detect a real difference in the data (Mundry, 2010). For more on sample size and statistical significance in behavioural ecotoxicology, see also EthoCRED reliability criterion #26 (Section III.5.t).

Beyond statistical significance, it is important to consider whether the size of an effect (i.e. the magnitude of difference between groups, or the strength of association between variables) is biologically or ecologically relevant. When studies fail to report effect sizes [e.g. Cohen's *d*, odds ratio, Pearson's correlation coefficient (*r*)] or fail to provide sufficient data to infer the effect size (e.g. mean and standard deviation of all groups), the results will be more challenging to interpret and the study therefore loses some relevance. Ideally, to aid expert judgement, it is good practice to make clear why the observed effect size(s) may (or may not) be biologically or ecologically relevant.

It is also worth noting that null‐hypothesis significance testing, which is the dominant method for statistical inference in many fields, including (behavioural) ecotoxicology, has received mounting criticism and the field is encouraged to move towards valid alternative methods that are less (or not at all) reliant on reporting of *P* values (e.g. confidence intervals, or credible intervals for Bayesian inference) (Erickson & Rattner, 2020). Hence, studies that do not report *P* values are not automatically irrelevant and evaluating the relevance of the reported results should always be done in light of the statistical method that was used.

#### (h) EthoCRED relevance criterion #9: are relevant life stages studied?

The life stage(s) of tested animals should be reported and appropriate to the experimental design, behaviours analysed, and purpose of the study. For instance, juveniles are typically more sensitive to the effects of chemical exposure than adults (reviewed in Mohammed, 2013). In addition, behavioural expression is plastic and typically develops/changes throughout the course of an animal's life (Thoré, Brendonck & Pinceel, 2020; Thoré *et al*., 2023b). For example, in terms of the behaviours tested, reproductive behaviours (e.g. courtship, mating events) should be studied in sexually active animals (i.e. excluding juveniles, or senescent animals which may no longer reproduce), and antipredator responses (e.g. light–dark preference, C‐start response) should be assayed in animals of a sufficient age to exhibit such behaviours. Likewise, sociability (e.g. shoaling tendency, group cohesion) should be tested in animals of an appropriate life stage (e.g. juveniles, sub‐adults, adults), given that many species display dissimilar social tendencies and social behaviours at different life stages. For instance, certain fish species shoal only during vulnerable, early life stages, while others live in groups throughout most or all of their lifespan (Ward, Kent & Webster, 2020). Further, amphibian species typically express different behaviours across their life cycle given that their juvenile and adult life stages may inhabit different environments (Johansson, Lederer & Lind, 2010). In studies involving experimental animals that have been collected from the wild, while the exact age may not be known, life stage may be inferred based on morphological and/or physiological traits that, for example, only manifest at sexual maturity (e.g. gonadosomatic index, secondary sexual characteristics such as mating colouration).

#### (i) EthoCRED relevance criterion #10: are the experimental conditions relevant for the tested species?

A study can only be considered relevant when the biology and ecology of the tested species are properly factored into the experimental design. This means that, other than the treatment under study, animals should be kept under optimal conditions that are tailored to the species and life stage under investigation, unless when deliberately manipulated (see also EthoCRED reliability criterion #11, Section III.5.g). For instance, depriving animals of their natural day–night cycle may disrupt their physiology and behaviour (e.g. under constant light exposure; Schligler *et al*., 2021), so that the response of the tested animals to chemical exposure may no longer be comparable to that of their wild counterparts. Likewise, social animals that are kept in isolation, or solitary animals that are kept in groups, may be stressed and/or no longer express their normal behaviour, so that it may become challenging to make meaningful predictions about the impact of chemical exposure in wild animals. A good understanding of the biology and ecology of the tested species is crucial to evaluate whether the experimental conditions are relevant for the species. Further, studies should ideally motivate why the methods are appropriate for the tested species to aid expert judgement, especially in the case of species that are not commonly studied.

#### (j) EthoCRED relevance criterion #11: if recovery is studied, is this relevant for the framework for which the study is evaluated?

Although recovery is not typically considered in most risk assessment frameworks, it is worth noting that behavioural expression is plastic and may – but does not always – change rapidly when changes in the environment occur (Wong & Candolin, 2015). This means that, while some behavioural changes may be permanent, others could be reversible and return to baseline values. For instance, the antidepressant fluoxetine affected foraging behaviour of hybrid striped bass (*Morone saxatilis* × *M. chrysops*) and this effect could still be observed 6 days after exposure had ceased (Gaworecki & Klaine, 2008), likely due to slow elimination of fluoxetine and its biologically more potent metabolite norfluoxetine from the central nervous system. By contrast, effects of the anxiolytic oxazepam on the swimming activity and boldness of burbots disappeared after a depuration period of 5–7 days (Sundin *et al*., 2019). This reversibility contrasts with some conventional endpoints in ecotoxicology, such as mortality and certain developmental abnormalities, which are irreversible. Recovery of behavioural traits could in theory also occur during prolonged/continued exposure. For instance, when chronically exposed to selective serotonin reuptake inhibitors such as fluoxetine, homeostatic responses in the brain could revert extracellular serotonin levels to a premedication equilibrium (Andrews *et al*., 2015) and lead to a return of behavioural expression to pre‐treatment levels. As such, results that indicate highly persistent effects of a chemical, even when exposure has ceased, can be used as supporting evidence in hazard and risk assessment. However, this does not necessarily hold in the opposite case (i.e. results that indicate rapid reversal of behavioural effects), not only because even a transient behavioural change may (in)directly have irreversible individual‐ and population‐level consequences (Wong & Candolin, 2015; Saaristo *et al*., 2018) but also because compensatory responses, such as development of resistance to chemicals, may come at a cost that could still negatively affect animal fitness (Kliot & Ghanim, 2012). Moreover, the assessment of recovery from exposure should be carried out in relation to the exposure profile (i.e. the concentration of a chemical, or chemicals, that the study organisms experienced throughout the exposure period, as a function of time).

#### (k) EthoCRED relevance criterion #12: is the tested exposure scenario relevant for the substance?

Adding to the CRED guidance for this criterion, the exposure route of the substance should be appropriate for the study organism and should be justified (e.g. waterborne, airborne, dietary). Further, direct injection of the test substance into animal tissues is less realistic in the context of environmental risk assessment of chemicals (Harris *et al*., 2014), and should therefore be avoided or appropriately justified.

#### (l) EthoCRED relevance criterion #13: is the exposure duration relevant and appropriate for the studied species and endpoints?

Studies investigating the behavioural effects of chemical exposure may be concerned with the immediate effects of short‐term (acute) exposure, the effects of continued (chronic) exposure, and/or delayed effects (i.e. those that are not observed until days or weeks after exposure, or epigenetic effects seen in subsequent generations). In contrast to classic ecotoxicology, no guidelines currently exist that define a standard exposure duration for behavioural studies in ecotoxicology, so that various exposure durations may be encountered in the literature, as well as various interpretations of what constitutes acute or short‐term exposure *versus* chronic or long‐term exposure. Importantly, the relevance and appropriateness of the exposure duration should be evaluated in light of the goal(s) of the study, the properties or environmental occurrence of the chemical compound, the studied endpoints, and the biology (e.g. life cycle) of the tested species. For instance, if the goal of the study is to assess the impact of exposure to a (pseudo‐)persistent chemical on an environmentally relevant timescale, a 2‐week exposure could be considered relevant in the case of a relatively short‐lived species such as *Daphnia*. Furthermore, biologically active chemicals (e.g. neuroactive drugs) may have a therapeutic delay rather than exerting an immediate response, so that acute or short‐term exposure may be less relevant, in particular if the compound persists in the environment. For instance, serotonin‐reuptake inhibitors (e.g. fluoxetine) may not only act directly through their pharmacological properties but also indirectly by delayed compensatory responses in the brain, which could take several weeks to develop (Andrews *et al*., 2015), as the substance slowly accumulates in brain tissue to therapeutically active levels.

### (4) EthoCRED reliability evaluation

The EthoCRED evaluation method uses four reliability categories: (*i*) reliable without restrictions, (*ii*) reliable with restrictions, (*iii*) not reliable, and (*iv*) not assignable. A description of these categories is provided in Table 3.

**Table 3:** EthoCRED reliability categories. Note that these categories correspond with the original Criteria for Reporting and Evaluating Ecotoxicity Data (CRED) reliability categories outlined by Moermond *et al.* (2016), which were adapted from Klimisch *et al.* (1997).

Reliability category	Description
Reliable without restrictions	All critical reliability criteria for this study are fulfilled. The study is well designed and performed, and it does not contain flaws that affect the reliability of the study.
Reliable with restrictions	The study is generally well designed and performed, but some minor flaws in the documentation or setup may be present.
Not reliable	Not all critical reliability criteria for this study are fulfilled. The study has clear flaws in study design and/or how it was performed.
Not assignable	Information needed to make an assessment of the study is missing. This concerns studies that do not give sufficient experimental details and that are only listed in abstracts or secondary literature (books, reviews, etc.) or studies for which the documentation is not sufficient for assessment of reliability for one or more vital parameters.

Using the EthoCRED method, reliability is evaluated according to 29 reliability criteria distributed across seven categories: general information, test setup, test compound or formulation, test organism, exposure conditions, assessing biological responses, and statistical design and analysis (Table 4). These criteria are adapted from the CRED evaluation method (Moermond *et al*., 2016). Qualitative assessment is used to determine the degree of fulfilment of each criterion. In cases where expert judgement may be required, this is indicated in the explanatory text accompanying each criterion. Although primarily intended for screening studies from the literature, the EthoCRED reliability criteria can also be used as a list of considerations for designing studies that are more likely to be useful for assessment and regulatory activities.

**Table 4:** EthoCRED reliability criteria^a^ for evaluating behavioural ecotoxicity data. The criteria are adapted and modified from the reliability criteria provided in the Criteria for Reporting and Evaluating Ecotoxicity Data (CRED) project (Moermond *et al.*, 2016). Additional guidance on how to interpret the EthoCRED reliability criteria is provided in the main text.

Number	Criterion
General information	
	Before evaluating a test, check the physicochemical characteristics of the compound (handbooks/general sources). What is the solubility, log K_OW_, or pK_a_? Is the compound volatile? Does it hydrolyse, photolyse, etc.?
Test setup	
1	Is a guideline method (e.g. OECD/ISO) or modified guideline used?^b^
2	Is the test performed under good laboratory practice (GLP) conditions?^b^
3	If applicable, are validity criteria fulfilled (e.g. control survival, growth, activity)?
4	Are appropriate controls performed (e.g. solvent control, negative and positive controls)?
Test compound or formulation	
5	Is the test substance identified by name or CAS number? Are test results reported for the appropriate compound?
6	Is the purity of the test substance reported? Or, is the source of the test substance trustworthy?
7	If a formulation is used or if impurities are present: do other ingredients in the formulation exert an effect? Is the amount of active substance or metabolites in the formulation reported?
Test organism	
8	Are the organisms well described (e.g. scientific name, mass, length, growth, age/life stage, strain/clone, sex if appropriate)?
9	Are the test organisms from a trustworthy source and, if relevant, acclimatised to laboratory conditions? Have the organisms not been pre‐exposed to the test compound or other unintended stressors?
Exposure conditions	
10	Is the experimental system appropriate for the test substance, taking into account its physicochemical characteristics?
11^†^	Is the exposure system appropriate for the test organism (e.g. choice of medium or test water, feeding, medium characteristics, temperature, light/dark conditions, pH, ammonia, dissolved oxygen)? Have conditions been kept stable throughout the exposure period?
12	Were exposure concentrations below the limit of water solubility (taking the use of a solvent into account)? If a solvent is used, is the solvent within the appropriate range and is a solvent control included?
13	Is appropriate spacing between exposure concentrations applied?
14	Is the exposure duration defined and appropriate?
15	Are chemical analyses adequate to verify concentrations of the test substance over the duration of the study?
16	Is the biomass loading of the organisms in the test system within the appropriate range (e.g. <1 g/l)?
Assessing biological responses	
17^†^	Is the behaviour‐testing environment appropriate for the experimental organism and research question(s) (e.g. size and shape of trial arenas, time window for testing, avoidance of chemical, visual, and auditory interference)?
18^†^	If relevant, was an acclimation period employed before behavioural trials?
19^†^	Is the duration of behavioural trials reported?
20^†^	For feeding and foraging trials, were animals fed an appropriate amount and at an appropriate time relative to the commencement of behavioural trials? Is the kind and quantity of feed/prey used reported and appropriate?
21^†^	In behavioural trials involving a predator, was an appropriate predatory stimulus used (e.g. was an anti‐predator response observed in controls?)?
22^†^	Were behavioural trials recorded (e.g. video and/or audio recordings)?
23^†^	Was/were the experimenter(s) blind to experimental treatment when conducting and analysing behavioural trials?
24^†^	If relevant, were experimental design elements appropriately randomised (e.g. assignment of animals to treatment groups, treatment type in behavioural trials, behavioural trial type in repeated testing, treatments across arenas in simultaneous testing, potential edge effects)?
25^†^	If animals were repeatedly tested using the same behavioural assay, were habituation effects accounted for?
Statistical design and analysis	
26	Is a sufficient number of replicates used? Is a sufficient number of organisms per replicate used for all controls and test concentrations?
27	Are appropriate statistical methods used?
28	Is a concentration–response relationship observed?
29	Are sufficient data available to check the calculation of endpoints and (if applicable) validity criteria (e.g. control data, raw data, dose–response curves)?

^†^ Criteria that specifically relate to behavioural ecotoxicity studies, which are additional to the original CRED criteria.

^a^ See main text for further explanation of the EthoCRED criteria and explanatory guidance text on how to interpret the criteria. Please note that most criteria are not *per se* critical for the reliability of a study and that this depends strongly on the compound and/or species tested.

^b^ These EthoCRED criteria are of minor importance for study reliability but may support study evaluation.

CAS, Chemical Abstracts Service; CRED, Criteria for Reporting and Evaluating Ecotoxicity Data; ISO, International Organisation for Standardisation; K_OW_, octanol–water partition coefficient; OECD, Organisation for Economic Co‐operation and Development; pK_a_, dissociation constant.

In general, the categorisation of a study as “reliable without restrictions” is appropriate when all essential information has been provided and the study exhibits no critical flaws in experimental design or outcomes. The classification of “reliable with restrictions” is appropriate for studies in which certain details may be lacking, raw data might not be available, or minor flaws in experimental design exist. Nonetheless, there remains a reasonable certainty that the results can be deemed reliable. It is important to underline that the labelling of studies as “reliable without restrictions” or “reliable with restrictions” is not exclusive to guideline and/or good laboratory practice (GLP) studies. A peer‐reviewed study, conducted and reported properly (regardless of GLP adherence), could warrant the “reliable without restrictions” label. Conversely, a guideline and/or GLP study that is executed or designed poorly should be categorised as “not reliable”. The designation “not assignable” is fitting when a study lacks essential details for reliability assessment but is not inherently unreliable.

A fundamental prerequisite for comprehensive evaluation is the accurate documentation of methods employed and the results obtained. Transparent reporting is valuable for making an efficient and thorough study evaluation, but it does not singularly dictate reliability assessment. Instead, an evaluation should be based on the details provided, rather than the clarity of the report, unless the description is so unclear that the methodologies are obscured. In this regard, a study that adheres to scientifically sound practices could be marked as “not assignable” if crucial methodological details are absent or if essential information for test result interpretation cannot be assessed and/or these data have not been retrieved by the assessor (Mensink, Smith & Montforts, 2008; Ågerstrand, Edvardsson & Rudén, 2013). If necessary and feasible, the authors of the study in question may be approached for the required details. However, additional information regarding a study, even if supplied, will not rectify known deficiencies in experimental setup or results. If flaws in study design or outcomes are present, additional information will not suffice to alter the categorisation to anything other than “not reliable”.

### (5) Explanation of the EthoCRED reliability criteria (criterion numbers from Table 4)

Only reliability criteria with EthoCRED‐specific guidance are reported. For the full list of criteria, including those that do not differ from the original CRED reliability criteria (Moermond *et al*., 2016), see Appendix S2.

#### (a) EthoCRED reliability criterion #1: is a guideline method (e.g. OECD/ISO) or modified guideline used?

Currently, behavioural endpoints are, with a few exceptions, not represented in guideline methods (discussed in Ågerstrand *et al*., 2020; Ford *et al*., 2021). Until they are, non‐standard studies need to be considered in environmental assessments, including chemical hazard and risk assessments, if behavioural endpoints – which are typically more sensitive than conventional ecotoxicological endpoints (reviewed in Melvin & Wilson, 2013) – are to be represented. Given this general lack of standardised methods for behavioural ecotoxicity testing, the reliability of a behavioural study should not be judged based on whether it is a guideline study or not. Instead, an evaluation of the test design, performance, and data analysis should determine its potential for use. Furthermore, the use of guideline tests that are adapted but not specifically developed for behavioural research may even result in reduced reliability when compared with non‐standard studies. This is, for example, the case when factors that may be crucial to a species' behaviour and/or ecology are not taken into consideration.

#### (b) EthoCRED reliability criterion #2: is the test performed under good laboratory practice (GLP) conditions?

Good laboratory practice promotes reproducibility and transparency but is not in itself a guarantee of high study reliability. Therefore, good laboratory practice should not be used as an argument to select or deselect non‐standard studies investigating behavioural effects (Moermond *et al*., 2016).

#### (c) EthoCRED reliability criterion #3: if applicable, are validity criteria fulfilled (e.g. control survival, growth, activity)?

This criterion particularly relates to studies that are conducted according to (modified) guidelines that include validity criteria. Behavioural studies are, with a few exceptions, performed in non‐standard settings that do not have predefined validity criteria. In the absence of validity criteria for a study, validity criteria from a guideline study may be used for guideline test species – although such validity criteria may also not be entirely relevant or possible to achieve given the often‐specialised experimental design and logistical requirements involved in behavioural ecotoxicity studies. In cases where modified guidelines are used, resulting in irrelevant or impossible validity criteria, expert judgement is needed to determine the potential impact of confounding factors. Importantly, any study with excess mortality in the control treatment(s) likely indicates an issue with experimental conditions or health of the study organisms. For a general discussion of issues relating to this criterion, see Moermond *et al*. (2016).

#### (d) EthoCRED reliability criterion #4: are appropriate controls performed (e.g. solvent control, negative and positive controls)?

Sufficient, appropriate controls are necessary for a study to be considered reliable. Typically, the control group receives no treatment but otherwise follows the exact same procedures as the vehicle and treatment groups, to enable direct comparisons. This means that, other than the treatment itself, all other procedures should be standardised (or randomised, when appropriate) across conditions to prevent systematic differences in behaviour due to factors other than the treatment under investigation. Examples of these potentially confounding variables include the age and/or life stage of animals (Peterson *et al*., 2017), the order in which individuals are allocated to experimental groups (Härkönen *et al*., 2016), the timing of behavioural observations with regard to potential daily fluctuations in behaviour (Thoré, Brendonck & Pinceel, 2021a), and the order of behavioural assays in cases where multiple behaviours are scored (Bell, 2013) (see also EthoCRED reliability criterion #24, Section III.5.r).

Expert judgement is needed to decide if mortality and behaviour of control animals falls within a range that can reasonably be expected. When relevant, studies should report on the number of mortalities in each treatment and how to interpret excess mortality or unexpected behaviour of control animals. However, behavioural ecotoxicity studies that do not report mortality are not necessarily unreliable, given that behavioural studies often use sublethal exposure concentrations, meaning that mortality has conventionally been reported less often because it is not an expected outcome of exposure. It should also be noted that, in behavioural studies, data points may be deleted from the final data set and considered as missing data in the case of errors during behavioural data collection (e.g. technical issues that may have affected behaviour or rendered recordings unusable). This does not threaten reliability as long as the final sample size is sufficiently large to establish the baseline variability in behaviour (Paull *et al*., 2008; Harris *et al*., 2014). However, reasons for such missing data should be reported and justified.

Other controls (e.g. positive controls, placebo controls) may be useful in some cases (e.g. Tanoue *et al*., 2019) but are not strictly required. For example, when testing known monoamine disruptors, positive controls such as serotonin or dopamine have been used along with their pharmacological agonists (e.g. Bringolf *et al*., 2010). Studies that make use of a solvent or vehicle to administer the chemical under investigation should include an appropriate solvent/vehicle control – that is animals that are treated with the solvent/vehicle alone at a concentration equal to that used in the primary experiment, with all other methods being equal. Statistical analyses should use the solvent/vehicle control as a benchmark of comparison (Harris *et al*., 2014). Lack of a solvent/vehicle control can be justified under some circumstances, for instance if historical data show no impact of the solvent/vehicle on the species/population under investigation at the administered dose, or if ethical and/or logistical constraints limit the number of test animals.

#### (e) EthoCRED reliability criterion #8: are the organisms well described (e.g. scientific name, mass, length, growth, age/life stage, strain/clone, sex if appropriate)?

As well as the traits specified in the CRED criteria, all of which can be associated with organismal behaviour, additional traits can influence behaviour and should therefore be specified where relevant. For instance, in assays of reproductive behaviour, the reproductive status (e.g. virgin, gravid, or non‐virgin) of the organisms under investigation should be described, given that reproductive status can influence reproductive behaviour and mating outcomes (e.g. Guevara‐Fiore, Skinner & Watt, 2009; Richardson & Zuk, 2023). Further, in assays involving interactions between multiple species (e.g. competitive or predator–prey interactions), it should be described whether the species under investigation co‐occur naturally in the environment (or may have experienced any previous encounters), because organisms are likely to behave differently when presented with a novel *versus* a familiar competitor, predator, or prey species (discussed in Sih *et al*., 2010; Ehlman, Trimmer & Sih, 2019).

In addition, the sex of experimental organisms should ideally be reported given that, in many species, the sexes exhibit distinctive behavioural repertoires and/or differ in the extent of expression of behaviours. The sexes, and their behavioural profiles, may therefore be differentially vulnerable to exposure to contaminants (e.g. Bertram *et al*., 2015; Martin *et al*., 2019a; Thoré, Brendonck & Pinceel, 2021b; Vossen *et al*., 2022). Potential sex differences in exposure can be accounted for by testing for potential behavioural changes in each sex separately, or by incorporating sex as a covariate in statistical models. Importantly, not accounting for, or reporting, sex does not automatically make a study unreliable, although justification should be given for why sex was not considered (e.g. the behaviour under investigation is known to be similarly expressed by males and females, or sex cannot be determined at a given life stage).

#### (f) EthoCRED reliability criterion #9: are the test organisms from a trustworthy source and, if relevant, acclimatised to laboratory conditions? Have the organisms not been pre‐exposed to the test compound or other unintended stressors?

Studies in behavioural ecotoxicology often use test organisms from a variety of different backgrounds, ranging from laboratory strains to outbred strains (e.g. crosses between laboratory strains and specimens from pet stores), and field‐collected animals. Apart from differences in genetic diversity among strains or populations, potential variation in historical exposure or handling means that organisms may already be adapted to some stressor(s) (Almeida *et al*., 2021) and/or that there could be confounding experiential/maternal effects (Bell, 2013). For instance, the behavioural response of animals adapted to a specific chemical may not reliably reflect that of specimens without such a history of exposure (see Hamilton *et al*., 2017). Further, long‐established laboratory strains that have partially or completely lost their antipredator defence mechanisms may not be appropriate for use in antipredator trials (see Vossen *et al*., 2020). Still, the availability of different strains or populations with different backgrounds allows for targeted research and may lead to a more robust overall conclusion on the environmental hazards posed by chemicals. Ideally, studies should provide a sufficient background description of the test organisms to facilitate expert judgement on the suitability of a particular strain or population (see EthoCRED reliability criterion #8, Section III.5.e), although a detailed account may not always be possible, particularly in the case of specimens from pet stores or field‐collected animals. Such a background description is therefore not a strict requirement, provided that all experimental groups share the same history and that the natural variability in behaviour is known (e.g. through the use of appropriate controls, see EthoCRED reliability criterion #4, Section III.5.d). However, providing no information on background conditions, such as a lack of samples taken to ensure the absence of contamination at the collection site(s) of animals from the field, does limit the reliability of a study.

Regardless of their origin, experimental animals should be healthy (e.g. with regard to parasite or pathogen load, unless this is part of the research question) and acclimated to the testing environment (e.g. housing conditions) to avoid stress that is associated with changing environmental conditions other than the treatment under study. Such unintended stress may be noticeable in mortality or aberrant behaviour of control animals and may render a study unreliable when not controlled for (see EthoCRED reliability criterion #4, Section III.5.d). Acclimation periods are very important in behavioural toxicity testing, for example, some species have circadian (or circatidal) rhythms that can take time to adjust to a laboratory setting. Experimenters must therefore be mindful of the time it takes for these rhythms to adjust to laboratory conditions, or fix/adjust daily recording times accordingly (see Thoré *et al*., 2023a). Likewise, in some instances, the longer specimens are removed from the wild, the more they may have habituated to laboratory conditions and the less “natural/normal” they may behave. The consequences of this are endpoint dependent and thus require a good understanding of the species' baseline behaviours.

#### (g) EthoCRED reliability criterion #11: is the exposure system appropriate for the test organism (e.g. choice of medium or test water, feeding, medium characteristics, temperature, light/dark conditions, pH, ammonia, dissolved oxygen)? Have conditions been kept stable throughout the exposure period?

The exposure system must be appropriate for the test organism for a study to be considered reliable. This means that, other than the chemical treatment under study, animals should be kept under optimal conditions throughout the experiment (unless this is part of the research question). Optimal conditions are often species‐ and life‐stage specific (Näslund & Johnsson, 2014; Thoré *et al*., 2020), so sufficient description of the test environment should be provided to facilitate expert judgement. Factors of interest include, but are not restricted to, dimensions of the housing environment (see EthoCRED reliability criterion #16, Section III.5.j), temperature, light/dark conditions (e.g. photoperiod, spectrum, light intensity) and, for aquatic organisms, water chemistry (e.g. electrical conductivity, pH, oxygen level), all of which may affect how animals behave and respond to chemical exposure. Furthermore, for tests on aquatic species, water quality measures (e.g. ammonia and nitrite levels) should be kept within appropriate ranges. Studies making use of physical enrichment – that is any physical complexity, such as substrate or refuges added to housing containers – should report sufficient characteristics (such as dimensions, ecological rationale, timing of enrichment, amount, inputs, and lighting; see Jones, Webster & Salvanes, 2021), and the social environment in which the animals are housed should be detailed, and justified, with regard to the number/density of conspecifics and the composition of groups (e.g. sex, age classes; see Martin & McCallum, 2021). Apart from being optimal, all of these conditions should be stable throughout the experiment with the notable exception of studies that deliberately use fluctuating environmental conditions to mimic natural conditions, such as daily temperature fluctuations (Verheyen, Delnat & Stoks, 2019). Stress related to suboptimal conditions of the exposure system, other than the treatment under study, may be noticeable in mortality or aberrant behaviour of control animals, and may render a study unreliable, especially if appropriate controls are not used (see EthoCRED reliability criterion #4, Section III.5.d).

It is also worth noting that in studies conducted under semi‐natural (e.g. mesocosms, enclosures) or natural conditions (e.g. whole‐lake exposures, field exposures), environmental conditions often cannot be strictly controlled, if at all. Given that ecotoxicology seeks to understand the effects of contaminants in the real world, this variability certainly does not detract from a study's reliability, although ideally it should be accounted for in the experimental design and statistical analysis where appropriate (e.g. temperature and/or light conditions may be included as covariates when modelling behavioural changes over time). These experimental design decisions include, where relevant, the selection of appropriate “control” sites for field studies to minimise differences in biotic and abiotic conditions with regard to “experimental” sites.

In contrast to acute toxicity studies in which animals are typically not fed, behavioural ecotoxicology studies often make use of chronic exposure to sub‐lethal concentrations, during which feeding is necessary. Feeding (including the type, amount, and frequency of food provided) should be appropriate for the species and life stage under investigation, and any excess food should be removed after feeding to avoid decreased bioavailability of the test substance (due to sorption), and to maintain good quality of the medium. Likewise, the frequency and method of cleaning the housing environment(s) should be appropriate and reported (e.g. frequency and proportion of water and treatment renewals in aquatic studies). In this regard, cleaning should be sufficient to maintain good quality of the medium and substance concentration, while not imposing any more stress on the animals than is absolutely necessary, and cleaning should be consistent across all (exposed and unexposed) treatment groups.

#### (h) EthoCRED reliability criterion #13: is appropriate spacing between exposure concentrations applied?

In ecotoxicology, it is common practice to characterise the dose–response of a substance, which requires a minimum of three to five exposure concentrations. However, it is always advisable to include more experimental treatment levels (OECD, 2006). When a sigmoidal (monotonic) curve emerges, a range of toxicity parameters [e.g. 50% lethal concentration/50% effective concentration (LC_50_/EC_50_), No Observable Effect Concentration (NOEC), Lowest Observable Effect Concentration (LOEC), benchmark dose calculations] can be calculated for use in environmental risk assessments (Harris *et al*., 2014). These parameters cannot be accurately calculated when the spacing between test concentrations is too small or too large, and it may therefore be necessary to perform an *a priori* range‐finding test to determine the necessary number of, and spacing between, exposure concentrations. In this regard, a scaling factor of 3.2 is typically recommended (with an upper bound of 10 as a rule of thumb; Moermond *et al*., 2016).

Importantly, while it is recommended to include such an exposure gradient when designing ecotoxicological studies, experiments in behavioural ecotoxicology are often more logistically complex than conventional ecotoxicity studies and may also face ethical constraints that limit the number of test animals and/or experimental treatments. It is therefore common, and acceptable, for studies in behavioural ecotoxicology to comprise just one or two exposure treatments, in addition to appropriate controls. This is acceptable provided that the exposure concentrations are relevant and justified – that is to demonstrate the absence or presence of effects at a certain concentration, or to characterise potential behavioural effects at an environmentally relevant dose. However, it is key for studies that are not designed to establish a dose–response relationship to abstain from making dose–response claims. When a non‐monotonic dose–response relationship emerges but the number of tested concentrations is limited due to logistical constraints, it becomes more important that those fewer concentrations producing non‐monotonic curves are repeatable and not spurious artefacts. More broadly, considering that changes to animal behaviour after sub‐lethal exposure to contaminants can elicit lethal outcomes – for example animals exposed to anxiolytic drugs that exhibit impaired anti‐predator behaviour and are therefore more likely to be consumed (e.g. Brodin *et al*., 2013; Martin *et al*., 2017) – it is essential for hormesis and other non‐linear dose–response relationships to be given proper credence when assessing behavioural studies. For a recent discussion of the importance of subthreshold effects in regulatory risk assessment, see Agathokleous *et al*. (2022). Also see EthoCRED reliability criterion #28 (Section III.5.v) for further discussion of dose–response relationships.

#### (i) EthoCRED reliability criterion #15: are chemical analyses adequate to verify concentrations of the test substance over the duration of the study?

It should be clearly described whether exposure to chemicals occurs before or during the behavioural evaluation, or both. In all cases, the original CRED criterion applies to the exposure medium. The reliability of a study may further improve when the uptake of the chemical(s) into the tissues of the exposed organisms is measured, in particular when the compound is known to bioaccumulate and if its accumulation in a specific tissue is expected to result in a behavioural effect (e.g. psychoactive or neurotoxic compounds accumulating in the brain/nervous system). For small organisms, where it may not be possible or practical to analyse accumulation of the compound in specific target tissues, whole‐body or pooled samples are acceptable in order to meet minimum biomass requirements for the analytical method that is employed.

Behavioural ecotoxicity experiments are often conducted at a much larger scale than typical ecotoxicity tests, in terms of the size and/or volume of the exposure arena(s) and the number of animals under investigation, in order to emulate natural conditions more closely. These experiments may also include natural substrates or environmental enrichment features that are not present in traditional ecotoxicity experiments. In order to achieve a higher degree of ecological realism of the exposure, there is a trade‐off with precise knowledge of exposure concentrations. For example, some exposure substances can partition between two or more compartments, such as water and sediment, affecting their bioavailability such that benthic animals may have a different exposure scenario than pelagic animals. Details about the exposure conditions and partitioning coefficients that could influence the test substance's bioavailability, bioaccessibility, or both should be provided, including for ionisable compounds tested across environmentally relevant pH gradients. Expert judgement should be used to determine the sampling strategy that best accounts for the life habits of the animal as it pertains to the bioavailability and/or bioaccessibility of the test substance, as well as the environmental compartments to be analysed.

#### (j) EthoCRED reliability criterion #16: is the biomass loading of the organisms in the test system within the appropriate range (e.g. <1 g/l)?

Biomass loading (i.e. the size and density of groups of conspecifics) is important in behavioural testing as it may affect the behaviours of the test organisms and their response to chemical exposure [see EthoCRED reliability criteria #11 (Section III.5.g) and #17 (Section III.5.k)], and loadings may also impact the exposure concentration (due to potential uptake or sorption of the chemical). The CRED biomass loading criterion is relevant to both the exposure and the behavioural testing phases of behavioural ecotoxicity studies.

#### (k) EthoCRED reliability criterion #17: is the behaviour‐testing environment appropriate for the experimental organism and research question(s) (e.g. size and shape of trial arenas, time window for testing, avoidance of chemical, visual, and auditory interference)?

To facilitate behavioural observation, animals are often transferred from an exposure system to one or more observational arenas. Many different assays and setups exist to score behaviour, which typically vary widely among studies and depend on the tested species and/or life stage. Because behaviour can be affected by many factors, and may reflect various underlying motivational and cognitive mechanisms, tests should ideally be validated for the life stage and species under investigation (Thoré *et al*., 2020). For instance, mirror‐tests are often used to assess fish aggressiveness (Balzarini *et al*., 2014) but may instead reflect sociability (Cattelan *et al*., 2017) or even self‐recognition (Kohda *et al*., 2022). When tests are not yet validated, they should at least be tailored to the biology of the test organism (e.g. open‐field arenas should be large enough for animals to be active and display exploratory behaviour without experiencing confinement stress; see also EthoCRED relevance criterion #5, Section III.3.d), and care should be taken when interpreting the results. To facilitate expert judgement, studies should provide full methodological details on the experimental setup, similar to the factors mentioned for the exposure system (see EthoCRED reliability criterion #11, Section III.3.g). For instance, the shape and dimensions of the observational arena(s), including the characteristics of the potential environmental enrichment, and social context (e.g. presence or absence of conspecifics and/or heterospecifics, density, group composition), should be reported given that this may affect how animals behave and respond to chemicals (Kohler *et al*., 2018; Henry *et al*., 2022; Michelangeli *et al*., 2022).

Likewise, abiotic environmental parameters, including temperature, light/dark conditions, and water chemistry (for aquatic studies) should be reported and kept constant throughout the assay and across trials. Ideally, these conditions should be similar to those of the exposure system to avoid stress that is associated with changes in environmental conditions other than the treatment under investigation, unless such changes are functional to the behavioural assay (e.g. testing the behavioural response to a change in temperature). In cases where wind tunnels or choice flumes are used to test attraction or avoidance in animals, it is critical that flows are not turbulent to prevent mixing of cues over the entire duration of the assay (Jutfelt *et al*., 2016). For tests on aquatic species, water quality measures (e.g. ammonia and nitrite levels) should be kept within appropriate ranges. Testing arenas for aquatic species should ideally also have their water partially or fully changed, and potentially be cleaned, between trials to avoid decreased bioavailability of the test substance and to maintain good quality of the medium (e.g. no build‐up of animal waste or cross‐contamination of chemical cues, maintenance of oxygen levels) [see EthoCRED reliability criteria #11 (Section III.3.g) and #16 (Section III.3.j)]. Importantly, water changes and/or cleaning should be consistent across all (exposed and unexposed) experimental treatments, to ensure comparability across treatments. Furthermore, arenas should be protected from potential unwanted disturbances (e.g. visual, auditory, vibrational), with the notable exception of multi‐stressor studies that may deliberately manipulate such additional stressors. Because behaviour can fluctuate diurnally (Melvin, 2017; Thoré *et al*., 2019a), trials should be conducted within a restricted time window to limit potentially confounding behavioural variation, and the timing of observations should be standardised (or randomised) across experimental conditions (Thoré *et al*., 2021a) unless doing otherwise can be justified (e.g. during a timeframe when it is known that there is no diurnal change). When the behaviour‐testing environment is not appropriate, this may be noticeable in aberrant behaviour of control animals and could render a study unreliable, although this may not always be noticeable and expert judgement is needed to make this evaluation.

#### (l) EthoCRED reliability criterion #18: if relevant, was an acclimation period employed before behavioural trials?

The timeframe over which experimental animals are allowed to acclimate to behavioural arenas (i.e. the behaviour‐testing environment) can influence the quality of behavioural data. At least two studies have explored the timeframe required for fish to reach a baseline behavioural status after being transferred into a new test environment, and these showed that optimising acclimation time led to improved baseline data for five different species (Melvin *et al*., 2017; Makaras *et al*., 2021). The consequence of insufficient acclimation is that response data (effect size) may reflect the combined impact of both the experimental treatment and general stress, leading to interpretations that cannot be extrapolated to natural environments with confidence. If exposure and data collection are performed in different systems (i.e. exposure system and behaviour‐testing environment), experimenters should report the acclimation time prior to the start of data acquisition. Species‐specific acclimation data should ideally be collected whenever possible to demonstrate that animals are exhibiting baseline behaviours and not experiencing stress associated with transfer to a new environment.

There exists a large diversity of behavioural endpoints and experimental systems (e.g. various testing apparatuses), and while these recommendations may be crucial for many standard study designs, there are also scenarios where short acclimation durations are highly relevant (and perhaps even critical to the study goal). For example, short acclimation times are inherently necessary for tests of anxiety or exploratory behaviour in a novel environment, such as with the well‐established novel tank diving test (Levin, Bencan & Cerutti, 2007). Similarly, it is also important to ensure that the duration of acclimation is not so long as to result in habituation effects (i.e. reduction in responsiveness), which can also lead to unreliable data (Blumstein, 2016; see EthoCRED reliability criterion #25, Section III.3.s). Hence, studies should ideally report the appropriateness of the acclimation period for the test organism (e.g. species, life stage) and the goal of the experiment.

#### (m) EthoCRED reliability criterion #19: is the duration of behavioural trials reported?

A wide range of study designs and test methodologies exists in behavioural ecotoxicology. Particularly the duration of behavioural trials (i.e. the length of time for data acquisition) is a factor that may vary widely and can influence the quality and validity of the measurements (Melvin *et al*., 2017). First, it is important for all individual behavioural measures to be compared among trials of a study that are of equal duration (i.e. standardised trial duration) to avoid unwanted variation and/or to prevent systematic differences in behaviour due to methodological differences [also see EthoCRED reliability criteria #4 (Section III.3.d) and #17 (Section III.3.k)]. Second, the duration of the behavioural trials should be appropriate for the behaviour under study and tailored to the biology of the test organism. For instance, fish activity level is often measured as travelled distance during a particular timeframe, commonly through assays of 10–20 min (e.g. Ansai *et al*., 2016; Thoré *et al*., 2019b; Tan *et al*., 2020). While behavioural measurement over a period of 10–20 min may yield a reasonably good approximation of the general activity level for many fish species, this may not be the case for all organisms (e.g. organisms that are more passive and/or show extended periods of inactivity) or behaviours (e.g. behaviours that may not be frequently expressed such as mating or displays of territoriality, or more complex behaviours such as nest building, may require longer study durations). Moreover, behavioural data collected over a short duration (e.g. <10 min) can potentially be statistically less powerful (and thus more prone to error) than data collected over longer durations (Melvin *et al*., 2017), which is particularly the case when short observation times lead to a low resolution of the data (e.g. zero‐inflated data sets). Because different observational timeframes may influence the overall conclusions of behavioural analysis (Melvin, 2017; Melvin *et al*., 2017), test durations should ideally be validated (i.e. robust and repeatable protocols) or justified in light of the species and life stage used, type, and overall context of the experiments that are conducted.

#### (n) EthoCRED reliability criterion #20: for feeding and foraging trials, were animals fed an appropriate amount and at an appropriate time relative to the commencement of behavioural trials? Is the kind and quantity of feed/prey used reported and appropriate?

Feeding and foraging trials are commonplace in behavioural ecotoxicology. At their simplest, these trials measure the quantity of food consumed and/or the time taken to consume food (e.g. Bertram *et al*., 2018b; Martin *et al*., 2019b; Bose *et al*., 2022a). The outcomes of such trials indicate both an individual's ability to feed and their motivation to feed. Thus, a critical consideration for feeding/foraging trials is standardising the hunger levels of animals before the trial. The experimenters should standardise the time since the animals were last fed, or were last given the opportunity to feed, prior to the beginning of the trial. Time since an individual last fed could alter its motivation to feed/forage (McNamara & Houston, 1986), and if not standardised, could introduce unwanted variability in the data. Thus, experimenters should report the food source, food quantity, and frequency of feeding in the lead up to the foraging/feeding trials. In standardising hunger levels, it is also important to consider what the motivational or energy homeostasis state of the animal should be during the time of the trial (Liu & Kanoski, 2018). For example, if animals are fed until satiation immediately prior to trials, there would be little to no motivation to feed during a trial. Therefore, there may be no observable differences across treatment groups simply because all animals were satiated. Therefore, it is ideal if the experiments withhold food for some amount of time before the feeding/foraging trial (the exact timing should be based on the biology of the model species). Another important consideration is the type and amount of food given to the animals during the trial. The amount and type of food provided to each animal should be consistent, or ideally normalised to the size of each individual (i.e. grams of food per gram body mass), and this information should always be reported.

#### (o) EthoCRED reliability criterion #21. In behavioural trials involving a predator, was an appropriate predatory stimulus used (e.g. was an anti‐predator response observed in controls?)?

Predator avoidance is a major aspect of prey decision‐making and behaviour (e.g. where, when, and for how long to forage), and can influence a range of important life‐history outcomes (e.g. reproduction, energy acquisition) (Lima, 1998). For this reason, studies in behavioural ecotoxicology are often interested in the effects of contaminant exposure on predator avoidance behaviours (e.g. freezing/inactivity, sheltering/hiding, threat/warning displays) as they are directly linked to survival probability. To elicit a typical fear or antipredator response in behavioural trials, researchers will often expose test subjects to a predatory stimulus. These stimuli can be visual, such as a predator model (e.g. Aimon *et al*., 2022) or a live predator placed behind a transparent barrier (e.g. Mason *et al*., 2021), tactile, through physical contact with a predatory stimulus (e.g. Orford *et al*., 2023), auditory, by playing back real or simulated predator sounds (e.g. Tai *et al*., 2023), or olfactory, such as predator chemical cues being added to the testing chamber (e.g. Saaristo *et al*., 2019). These can also be combined, such as predator visual and olfactory stimuli, which typically elicits a more pronounced anti‐predator reaction (e.g. Fursdon *et al*., 2019; Cerveny *et al*., 2020). A range of other anxiety‐inducing stimuli are also commonly used in assays testing anti‐predator or fear responses, including for example objects that are dropped into a testing chamber to elicit an escape response (e.g. Martin *et al*., 2017). Importantly, predatory stimuli should be ecologically relevant and replicate a threat that the prey species has experienced, either through a recent encounter with the actual predator, or in its evolutionary past. If test subjects are naïve to the predator stimuli, this will likely not elicit a relevant or typical antipredator response (Sih *et al*., 2010). Lastly, studies can also allow prey and predators to interact freely in behavioural trials, in which case predation may be directly observed (e.g. Lagesson *et al*., 2018). This is relatively rare due to ethical constraints, and so is more often seen with, for example, invertebrate prey (e.g. Bose *et al*., 2022b).

There are several key considerations when assessing the appropriateness of the predatory stimulus used in a behavioural trial. These include the ecological relevance of the chosen predator stimuli (e.g. does the prey species encounter the predator in its natural environment?), how well the predator stimulus replicates a natural predator cue (e.g. is the model predator a realistic size, shape, and colour?), and the signal capacity of the stimuli for prey detection (e.g. can the prey species adequately detect and recognise the stimuli?). At a minimum, a description of the predator stimulus should be included in the methodology of the research paper, and ideally, the above considerations should also be adequately addressed.

#### (p) EthoCRED reliability criterion #22: were behavioural trials recorded (e.g. video and/or audio recordings)?

Experimental trials in research fields such as behavioural ecology have conventionally been scored live by one or more observers. Although manual scoring does not necessarily render a study unreliable, it may be prone to bias and have a comparatively low reproducibility (see Henry & Wlodkowic, 2020; Bownik & Wlodkowic, 2021b). Manual scoring can also present a heightened risk of external interference with animal behaviour during trials, for example due to visual or auditory disruptions produced by the observer(s). Researchers using manual scoring should therefore take appropriate measures to counter such pitfalls as much as possible, for instance by adopting blind scoring (see EthoCRED reliability criterion #23, Section III.3.q) and appropriate randomisation (see EthoCRED reliability criterion #24, Section III.3.r), as well as reducing any potential for disturbances (e.g. by scoring behaviour from behind a screen). As an alternative to manual scoring, digital data acquisition is increasingly adopted to obtain behavioural measures (Henry, Rodriguez & Wlodkowic, 2019; Simão *et al*., 2019; Henry & Wlodkowic, 2020), reducing data collection errors and bias, and therefore increasing the reliability and reproducibility of results (Henry & Wlodkowic, 2020; Bownik & Wlodkowic, 2021b). Electronic data recording can be performed using diverse opto‐electronic and digital video‐recording systems (reviewed in Bownik & Wlodkowic, 2021, 2021), as well as using remote‐sensing systems such as acoustic telemetry (Bertram *et al*., 2022; Hellström *et al*., 2022) or sound‐triggered recordings of vocalisations (Hoffmann & Kloas, 2010). Video recording using digital cameras is, in this context, applicable for most behavioural ecotoxicity experiments conducted in laboratory conditions. Moreover, coupling video recording of behavioural trials with subsequent analysis of trial videos using animal‐tracking software provides not only digitally recorded data archives but also relatively unbiased analytical workflows (Henry & Wlodkowic, 2020).

From the perspective of behavioural ecotoxicity trial reporting, authors should ideally provide information on how the recording was conducted, although absence of such information does not necessarily render a study unreliable. For instance, authors should preferably report the make and model of camera and the settings used (e.g. resolution of the camera sensor, frame rate of the video, sensitivity of the sensor), what illumination sources were used and their parameters (e.g. light intensity, spectral profile of the light source), and what kind and version of animal‐tracking software was used (if any), including key settings (e.g. whether smoothing filters were applied, how missing tracks were dealt with).

#### (q) EthoCRED reliability criterion #23: was/were the experimenter(s) blind to experimental treatment when conducting and analysing behavioural trials?

Studies in behavioural ecotoxicology typically involve one or more researchers conducting experimental trials that comprise observing animals and scoring the frequency and/or duration of behaviours of interest. Trials may be observed live and/or video recorded, with behaviours being scored by hand or, more commonly, using behaviour‐analysis software – often including programs that log key presses (e.g. BORIS; Friard & Gamba, 2016). In general, the experimenter(s) should be blinded to the experimental treatments (e.g. chemical exposure) when conducting and analysing behavioural trials in order to avoid potential bias (Holman *et al*., 2015; Parker *et al*., 2016). Blinding removes both conscious and unconscious biases, including confirmation bias, where an experimenter may preferentially detect and focus on outcomes that confirm prior beliefs (Nickerson, 1998). In practical terms, blinding can be accomplished by, for example, codifying experimental animals, exposure containers, trial arenas, and/or video recordings.

Non‐blinding (or incomplete blinding), however, does not automatically make a study unreliable. For instance, although rare in behavioural ecotoxicity studies, blinding may not have been possible. This is the case when the experimental design involves treatments that are perceptible by the observer (e.g. visible chemical exposures such as wastewater effluent, male *versus* female in sexually dimorphic species, single animal *versus* group, small *versus* large animal), in which case the observer(s) should be blind to the hypothesis of the experiment. Alternatively, blind observation may have been used but not reported (discussed in Kardish *et al*., 2015). This is difficult or impossible to disentangle from not having used blinding and should therefore be avoided by researchers. Furthermore, increasingly advanced behavioural analysis software options for quantifying animal behaviour (reviewed in Bertram *et al*., 2022) may limit or remove the potential for experimenter effects during the behaviour‐scoring process, therefore partially or fully removing the need for blinding at this step. For example, software designed to track animals individually or in groups is increasingly being used in behavioural ecotoxicology research (e.g. ToxTrac; Rodriguez *et al*., 2018). In the case that analysis of video files of recorded behavioural trials is fully automated, blinding of this experimental stage is not necessary. However, if the analysis is only partially automated, including if experimenters manually “corrected” inaccurate software‐generated animal tracks, any manual interaction with data collection and/or analysis should be blinded. Moreover, given the wide variety of video‐tracking software options available, it is important that the name of the tracking software used is reported, as well as the version number where relevant.

#### (r) EthoCRED reliability criterion #24: if relevant, were experimental design elements appropriately randomised (e.g. assignment of animals to treatment groups, treatment type in behavioural trials, behavioural trial type in repeated testing, treatments across arenas in simultaneous testing, potential edge effects)?

Animal behaviour, including behavioural responses to chemical exposure, is typically sensitive to a wide range of environmental and experiential factors (Bell, 2013). For instance, juvenile Nile tilapia (*Oreochromis niloticus*) reared at a high density were more neophobic and less aggressive compared to conspecifics that were reared at a low density, and these effects were more pronounced when tested in an arena without shelter compared to that with shelter (Champneys *et al*., 2018). It is therefore necessary that, where at all possible, all procedures and conditions (other than the experimental treatment itself) are standardised for a study to be considered reliable (see also EthoCRED reliability criterion #4, Section III.3.d). To control further for the effect of extraneous/confounding variables and to avoid systematic errors (e.g. related to the order and/or timing of behavioural trials), experimental design elements should be appropriately randomised, as much as possible. For instance, the position of exposure and behavioural trial arenas should be randomised by treatment, wherever possible, to ensure that lighting, noise, and any other potential stimuli are as consistent as possible across the treatments – although potential extraneous stimuli should also be reduced as much as possible. Further, behavioural trials cannot be performed for all animals simultaneously and several rounds of observation (spread out over time) are necessary, meaning that trials should be performed in a random order with relation to the experimental condition of the animals (rather than first scoring all control animals, then scoring animals that were exposed to compound concentration 1, and so on). This randomisation over time is also important to control for potential diurnal changes in behaviour (Thoré *et al*., 2021a; see also EthoCRED reliability criterion #17, Section III.3.k). In cases where multiple behaviours are scored (i.e. when animals are successively subjected to different behavioural assays), researchers can either randomise the order of the assays or adopt a fixed order (the advantages and disadvantages of which are discussed in detail by Bell, 2013). However, systematic differences in the ordering of assays with respect to the experimental treatment render a study unreliable (unless sufficient justification is given, which may require expert judgement).

#### (s) EthoCRED reliability criterion #25: if animals were repeatedly tested using the same behavioural assay, were habituation effects accounted for?

Because behaviour is a labile trait that naturally varies across time and context, researchers may adopt repeated‐measures designs in which individuals are repeatedly tested using the same behavioural assay to increase statistical rigour and/or to account for intraspecific variation that extends beyond mean behavioural change (e.g. Polverino *et al*., 2021; Thoré *et al*., 2021c). However, animal responsiveness to repeated stimulation (such as during a series of behavioural assays) can progressively decrease as animals may acclimate/habituate to the setup and/or lose sensitivity to stimuli after continual exposure (Raderschall, Magrath & Hemmi, 2011; Bell & Peeke, 2012). For instance, the novel object test relies on avoidance/inspection behaviour exhibited towards an unfamiliar object in a familiar environment and is a standard paradigm to score neophobia/boldness in various animals (Frost *et al*., 2007; Brunet *et al*., 2022). When animals are repeatedly presented with the same stimulus (i.e. the same object), they may over time learn to recognise the object and adjust their response accordingly. This means that the scored behaviour may no longer reflect the same underlying motivational mechanisms as during the original trial, which could complicate interpretation of the results. Furthermore, individuals can vary in their habituation rates (Bell & Peeke, 2012), and habituation speed could conceivably vary with chemical exposure, either of which could further confound the results of the test. Habituation effects do not necessarily mean that a study is unreliable, but they should be accounted for statistically (e.g. testing how behaviour changes across trials with relation to the experimental treatment) and/or factored into the interpretation of the results.

Apart from habituation, acclimation, and/or sensory adaptation, repeated behavioural trials may also burden or fatigue animals, in particular when stimuli are used that are intended to startle, disturb, or otherwise stress animals (e.g. repeated trials with a predator stimulus absent and then present), and cause them to behave differently compared to earlier trials or to animals that were not subjected to the same stimuli. For this reason, studies can only be considered reliable when animals were given enough time to recover between trials, unless rapid retesting is appropriately justified and instrumental to the research goal. What is appropriate timing between trials depends on several factors, including the species, life stage, and type of behavioural test, and expert judgement is needed to make this evaluation.

#### (t) EthoCRED reliability criterion #26: is a sufficient number of replicates used? Is a sufficient number of organisms per replicate used for all controls and test concentrations?

Since individual organisms can differ substantially in their behavioural responses, behavioural data are often characterised by high levels of variability, meaning that large sample sizes may be required to ensure sufficient statistical power and to avoid generating spurious effects (Jennions & Møller, 2003; but see Melvin & Wilson, 2013). In statistics, power refers to the probability that a hypothesis test can detect the existence of a true effect. However, research has shown that the power of behavioural studies can be very low (Jennions & Møller, 2003). This means that if sample sizes are small, a non‐significant result does not necessarily mean that there is no effect; it could simply reflect low statistical power due to insufficient replication. Issues of statistical power are especially pertinent when evaluating the reliability of non‐significant results, as there is a risk of committing type‐II error (i.e. erroneously concluding that there is no effect). Such a possibility can be influenced by the kinds of statistical test(s) employed (Jennions & Møller, 2003), as well as whether and how statistical corrections are applied for multiple tests or comparisons (which can further exacerbate the likelihood of type‐II errors; Nakagawa, 2004). Of course, study design is also important, and, from a replication standpoint, even a statistically significant result may be unreliable if the results are based on a study design where the wrong entity has been replicated (i.e. pseudoreplication; *sensu* Hurlbert, 1984). Therefore, when considering whether there is a sufficient number of replicates, it may be prudent also to consider what, exactly, the unit of replication in the experiment is (e.g. the number of individual animals *versus* the number of enclosures in which the animals are being housed or exposed; see Marshall, 2024). The latter may be especially pertinent in the case of social species, where the experimental unit of replication will be at the group (rather than individual) level if animals are being tested collectively as a group. In this regard, the level of replication on which statistical analysis is based (e.g. the number of individuals or groups) is determined by the study design and the type of statistical test being performed. Expert judgment is sometimes required to determine if the replication level is appropriate.

#### (u) EthoCRED reliability criterion #27: are appropriate statistical methods used?

The use of appropriate and accepted statistical tests is critical for the robust evaluation of behavioural toxicity data. In any study, the choice of statistical methods should reflect the nature of the data (e.g. categorical, binomial, count), any underlying assumptions of the statistical test have to be met, and any potential biases or interpretive errors should not be introduced through the analysis. At the broadest level, statistical analyses typically involve a choice between parametric and non‐parametric methods. Parametric methods are commonly applied to hypothesis testing [e.g. *t*‐test, analysis of variance (ANOVA)] but rely on underlying assumptions about the characteristics of the data (e.g. error distribution, homogeneity of variance, minimum number of replicates) which must be met for the test to be valid. Should data fail to meet these assumptions, an equivalent and appropriate non‐parametric method should be used. The experimenter must demonstrate that the data have been carefully considered such that the choice of analysis is suitable, and any transformation or normalisation steps applied to the data should be reported in full.

For studies that aim to make dose–response claims [see also EthoCRED reliability criteria #13 (Section III.3.h) and #28 (Section III.3.v)], the study design and subsequent statistical analysis should allow for the determination of a reliable concentration that produces a given level of effect (EC, effective concentration), or alternatively, a concentration below which the effect is not distinguishable from background noise. Such values should be derived from interpolation rather than extrapolation, which implies that the EC falls between the lowest and highest concentrations tested in the study. Concentration–response modelling methods (e.g. regression) are generally preferable over hypothesis‐testing methods (e.g. *t*‐test, ANOVA) for determining a reliable EC, but these have their own sets of assumptions, including that the response follows a monotonic concentration–response pattern [i.e. there is no change in the sign (positive/negative) of the slope over the range of concentrations tested]. It is also common for studies to test just a few concentrations because of logistical (hence power) constraints, in which case ANOVA or mixed modelling approaches may be used, with concentration as a fixed factor rather than as a continuously distributed variable. When using such an approach, *post‐hoc* tests among concentrations can be very informative.

Data in behavioural ecotoxicology – and ecotoxicology more generally – are typically hierarchically structured. This means that multiple exposure containers are usually assigned to each treatment group, and multiple individuals are usually assigned to each of those exposure containers, and (where applicable) multiple measures may be taken for each individual. Statistical analyses should account for this hierarchical structuring in order to make accurate inferences about the effects of contaminants on endpoints of interest. For instance, in cases where the behaviour of individual organisms is tested repeatedly, care should be taken to make sure that this is accounted for in statistical analysis – for example through the use of mixed‐effects models, which can also be called “hierarchical” models or “random‐effects” models (reviewed in Arnqvist, 2020). This approach is necessary if data are collected repeatedly from the same individuals and used in the statistical analyses because these data are non‐independent. However, mixed‐effects models may not always be necessary in cases where data‐reduction approaches have been used (e.g. if all observations of each animal have been averaged).

Numerous resources provide discussion of statistical principles and commonly used techniques in ecotoxicology (OECD, 2006; Green, Springer & Holbech, 2018). When missing values or problematic data are encountered, consultation with an experienced and qualified statistician is recommended to ensure robust and reliable interpretation of behavioural data.

#### (v) EthoCRED reliability criterion #28: is a concentration–response relationship observed?

The concentration–response (dose–response) relationship is a key principle in toxicology (see also EthoCRED reliability criterion #13, Section III.3.h), and a critical component of regulatory toxicology since it helps to demonstrate causality between chemical exposure and a biological effect. A quality concentration–response curve should comprise a broad range of test concentrations, including a dose below which there is no effect (NOEC). With no concentration–response curve, or a very limited curve (few concentrations tested and not spanning a NOEC), it is not possible to establish a meaningful EC from behavioural toxicity data. Difficulties also arise when the concentration–response relationship is non‐monotonic; in other words, when the slope of the curve changes sign (positive/negative) along the concentration gradient tested – as is, for example, often the case when studying endocrine‐disrupting chemicals. In such instances, there may be uncertainty regarding the potential for effects at very low concentrations and it is not possible to establish a reliable EC. Contrarily, non‐monotonicity at high concentrations, but a linear relationship at lower concentrations, should not influence the ability to characterise risk and, thus, to establish an EC.

Behavioural toxicology studies are applied very broadly and are not always intended to establish absolute dose–response relationships. In many cases, behavioural endpoints may be explored when such relationships exist for other traditional endpoints. In these scenarios, a researcher may be interested in characterising potential behavioural effects towards the lower end of an existing dose–response curve and/or at an environmentally relevant dose, and therefore a wide concentration gradient is not required for behavioural data to offer additional evidence of risk. Similarly, if the goal of a study is to verify a lack of response at a specific concentration (e.g. with increased replication), a concentration–response relationship may not be necessary for a study to provide meaningful information. This may be particularly relevant for behavioural data since it can be logistically difficult to achieve adequate replication for robust analysis while also including a wide range of concentrations, and since behavioural responses are often more sensitive than other endpoints (e.g. developmental or reproductive traits; Melvin & Wilson, 2013). Thus, behavioural data without concentration–response relationships can provide meaningful evaluation of an EC calculated from non‐behavioural data, but the identification of a response at or below an existing NOEC would signify the need for further behavioural testing with an acceptable concentration gradient to establish a valid behavioural EC. As mentioned above (see EthoCRED reliability criterion #13, Section III.3.h), it is also important not to discount sub‐threshold effects in chemical risk assessments just because a non‐standard dose–response is observed (Agathokleous *et al*., 2022).

#### (w) EthoCRED reliability criterion #29: are sufficient data available to check the calculation of endpoints and (if applicable) validity criteria (e.g. control data, raw data, dose–response curves)?

Journals and funding agencies are increasingly mandating that authors make the raw data and statistical code used to obtain their results publicly available (e.g. in archived data repositories; Bertram *et al*., 2023). Data sharing allows for greater transparency and replication of experiments, which can increase trust in published findings and promote collaboration and further advances (Bertram *et al*., 2023). It is important to realise, however, that the absence of raw data does not, in itself, mean that a study is unreliable, especially when considering that, historically, the availability of such data was not a prerequisite for study publication and, in some fields, may still be a relatively uncommon practice. Rather, the availability of raw data can contribute to an assessment of a study's reliability by allowing readers to understand, evaluate, and reproduce a study's findings and conclusions (Gomes *et al*., 2022). Beyond transparency in sharing raw data for all response variables measured in a test, it is also valuable to include data relevant to quality assurance and control [e.g. analytical verification of exposure concentrations, see EthoCRED reliability criterion #15 (Section III.3.i); use of appropriate positive and/or negative controls, see EthoCRED reliability criterion #4 (Section III.3.d)].

It is important also to emphasise that the mere availability of raw data is not a guarantee of a study's reliability. Here, it is critical to consider the quality of the provided data. In this regard, the current onus on data archiving, along with creating clear and complete archives, typically rests with authors. As a result, the quality of raw data that are shared can be highly variable and may not always be sufficient to enable the study's reliability to be properly evaluated (e.g. authors sharing incomplete and/or indecipherable data sets, or providing summary statistics instead of actual raw data).

Consequently, in line with the original CRED reliability criterion #20 (Moermond *et al*., 2016), the availability of raw data is not a prerequisite for a study to be reliable, although it is certainly beneficial for researchers to make their data publicly available for the aforementioned reasons.

## IV. ETHOCRED REPORTING RECOMMENDATIONS

The EthoCRED reporting recommendations have been formulated as an extension of the original CRED reporting recommendations (Moermond *et al*., 2016) and encompass 72 criteria distributed across seven categories: general information, test design, test compound, test organism, exposure conditions, assessing biological responses, and statistical design and analysis (Table 5). These reporting recommendations have been designed to align with the reliability criteria from the EthoCRED evaluation method, meaning that the guidance material associated with the reliability evaluation method is also a useful resource for researchers in behavioural ecotoxicology when designing their studies. In this regard, researchers undertaking behavioural ecotoxicity studies are advised to familiarise themselves with the EthoCRED reporting recommendations during the early phases of experiment design to ensure that all factors contributing to reliability are considered.

**Table 5:** The EthoCRED reporting recommendations, containing 72 specific aspects to consider when reporting behavioural ecotoxicity studies. Importantly, not all recommendations are relevant for every study, but it is good practice to address the relevant criteria in a clear and transparent fashion. In cases where certain relevant reporting recommendations cannot be addressed, EthoCRED recommends that authors transparently explain the reason(s) behind the omission of this information.

EthoCRED reporting recommendations	
1	General information
	a. Purpose of study
	b. Description of endpoints
	c. Biological/ecological basis for the behavioural endpoint(s) investigated, with supporting evidence
	d. Population‐level relevance of the behavioural endpoint(s) investigated, with supporting evidence
2	Test design
	a. Performed according to standard/modified standard (e.g. OECD, USEPA)
	b. Performed according to good laboratory practice (GLP)
	c. Description of control(s): negative control, solvent control, positive control
	d. Control(s) mortality, growth, morbidity, and other observed non‐standard effects such as changes to colouration
	e. Comparison to validity criteria (e.g. control survival, growth) from appropriate guideline test method
3	Test compound
	a. Identification (e.g. name, CAS number, specify if the salt or the base is tested)
	b. Physicochemical characteristics that may influence the behaviour of the compound during the study [e.g. solubility, volatility, stability (hydrolysis, photolysis, degradation), solubility, log K_OW_, degradability, adsorption]
	c. Source (e.g. manufacturer, product code)
	d. Purity percentage
	e. Composition of product formulation and presence of impurities
4	Test organism
	a. Scientific name
	b. Relevant morphological characteristics (e.g. body mass, length)
	c. Age/life stage
	d. Growth/reproductive condition
	e. Sex
	f. Strain, clone
	g. Source (e.g. wild‐collected, laboratory stock, commercial supplier), ideally including analytical chemistry verification ruling out or characterising potential pre‐exposure of organisms to the test compound/other contaminants
	h. Acclimation to laboratory conditions (e.g. duration, feeding, housing conditions)
5	Exposure conditions
	a. Exposure route (e.g. waterborne, soilborne, airborne, dietary, and/or injection)
	b. Exposure schedule (static, semistatic, flow‐through system, other) and flow rate (flow‐through systems) or renewal time (semistatic systems)
	c. Open or closed system
	d. Test medium composition (e.g. source of test water: well water, deionised water, tap water)
	e. Temperature and time points for measuring
	f. pH and time points for measuring
	g. Hardness of water and time points for measuring
	h. Conductivity/salinity and time points for measuring
	i. Dissolved oxygen content and time points for measuring
	j. Light intensity and quality (e.g. source, light spectrum, homogeneity), light/dark conditions
	k. Feeding protocols, food composition
	l. Material and volume of aquarium/container and other equipment in contact with test organisms and test substance
	m. Use of sand or sediment and its characteristics (e.g. total organic carbon, particle size)
	n. Preparation of stock solutions, including solvent concentrations in test water and controls for aquatic studies
	o. Nominal concentrations of test substance
	p. Measured concentrations of test substance and time points for measuring, including exposure media (e.g. water, soil, air) and organism tissues (e.g. brain, muscle, liver)
	q. Analytical method: description of method, including limit of detection and limit of quantification
	r. Exposure duration and total test duration
	s. Time points of observations for endpoints (behavioural and non‐behavioural)
	t. Results based on nominal or measured concentrations
	u. Biomass loading (biomass per litre)
	v. Exposed individually or in a group within each exposure container, including number of individuals per exposure container
	w. Composition of groups when exposed with multiple individuals per container (e.g. single *versus* multiple species and/or sexes)
6	Assessing biological responses
	a. Size and shape of behaviour trial arenas
	b. Time window of behavioural testing (e.g. daily start and stop times of observation)
	c. Measures taken to avoid chemical, visual, and/or auditory interference
	d. Use and duration of acclimation period to behavioural trial arenas before the commencement of trials
	e. Duration of behavioural trials
	f. Description of feeding regime before – and, if relevant, during – behavioural trials, particularly important for feeding and foraging behaviour trials
	g. Whether organisms continued to be exposed, or not, to the treatment(s) throughout some or all of the behavioural assay(s)
	h. Behavioural trials carried out on individuals or groups, including number of individuals per trial
	i. For behavioural trials on groups, composition of groups (e.g. single *versus* multiple species and/or sexes)
	j. For trials involving a predator stimulus, the species, size, and type of stimulus used (e.g. live predator, model predator, predator animation)
	k. For trials involving a predator, use of visual, chemical, physical, or auditory predator cues, or a combination
	l. For trials involving a predator, ecological relevance of the predator species. Are they sympatric with the prey model species in the wild? Was an anti‐predator response seen in controls?
	m. Details of recording of behavioural trials (e.g. video and/or auditory recording) and/or live observation
	n. Details of analysis of behavioural trials [e.g. software name and version, software parameterisation, manual or human scoring from videos (e.g. using JWatcher or BORIS) or supervised automated approaches (e.g. EthoVision XT, ZebraBox, ToxTrac)], and/or live observation
	o. Biological response for each concentration and assay
	p. Blinding of experimenter(s) conducting and analysing behavioural trials (e.g. partial or full blinding, non‐blinded)
	q. Randomisation of experimental design elements, if relevant (e.g. assignment of animals to treatment groups, treatment type in behavioural trials, behavioural trial type in repeated testing, treatments across arenas in simultaneous testing, potential edge effects)
	r. Accounting for potential habituation effects (if animals were repeatedly tested using the same behavioural assay)
7	Statistical design and analysis
	a. Number of replicates for control(s) and test concentrations; setup of replicates (avoid pseudoreplication)
	b. Number of organisms per replicate
	c. Treatment design (e.g. block, randomised)
	d. Statistical methods used
	e. Dose–response observed
	f. Statistically significant responses noted (e.g. EC*x*)
	g. Significance level for NOEC and LOEC data (*α* = 0.05, or less)
	h. Estimation of variability for LC*x* and EC*x* data
	i. Availability of raw data: through supporting information, a website, or upon request (statements of data availability upon request should be avoided wherever possible)

CAS, Chemical Abstracts Service; CRED, Criteria for Reporting and Evaluating Ecotoxicity Data; EC*x*, *x*% effective concentration; K_OW_, octanol–water partition coefficient; LC*x*, *x*% lethal concentration; LOEC, Lowest Observable Effect Concentration; NOEC, No Observable Effect Concentration; OECD, Organisation for Economic Co‐operation and Development; USEPA, US Environmental Protection Agency.

Certain EthoCRED reporting recommendations are crucial for the reliability of a given study, while others carry relatively less weight. The significance of certain reporting recommendations often depends on experimental design variables such as the test organism, test duration, or test substance. For instance, specifying the sex and life stage of organisms tested for the effects of exposure to endocrine‐disrupting chemicals is of high significance given that various endocrine disruptors are known to have effects that are dependent on these parameters, while reporting the parameters of behavioural software would be irrelevant in a study that used a manual, software‐free behaviour‐scoring approach. Authors reporting behavioural ecotoxicity studies are strongly encouraged to incorporate a comprehensive and well‐structured description of their experiments, supplemented with additional data if necessary. In cases where certain relevant reporting recommendations cannot be addressed, it is recommended that authors transparently explain the reason(s) behind the omission of this information. By doing so, evaluators of the study – such as peer reviewers, editors, fellow researchers, and risk assessors – can more easily assess the experimental design, outcomes, and potential limitations of the study. Adherence to the EthoCRED reporting recommendations is expected to reduce the potential for underreporting and information gaps within a published study. Furthermore, it is likely that a study following the EthoCRED reporting recommendations will undergo the peer‐review process more efficiently.

## V. DISCUSSION

### (1) EthoCRED evaluation method

Behavioural endpoints represent a sensitive and ecologically meaningful addition to the standard endpoints used in hazard and risk assessment (Ågerstrand *et al*., 2020; Ford *et al*., 2021). Despite this, uptake of behavioural studies in hazard and risk assessment has been sparse, with, for example, just six identifiable cases in European Union chemical regulation where behavioural endpoints have contributed in some way to decision making (Ågerstrand *et al*., 2020). This means that the over 3,600 behavioural ecotoxicity studies performed between 2000 and 2023 (Fig. 1) have largely been excluded from use in an environmental protection context.

**Fig. 2:**
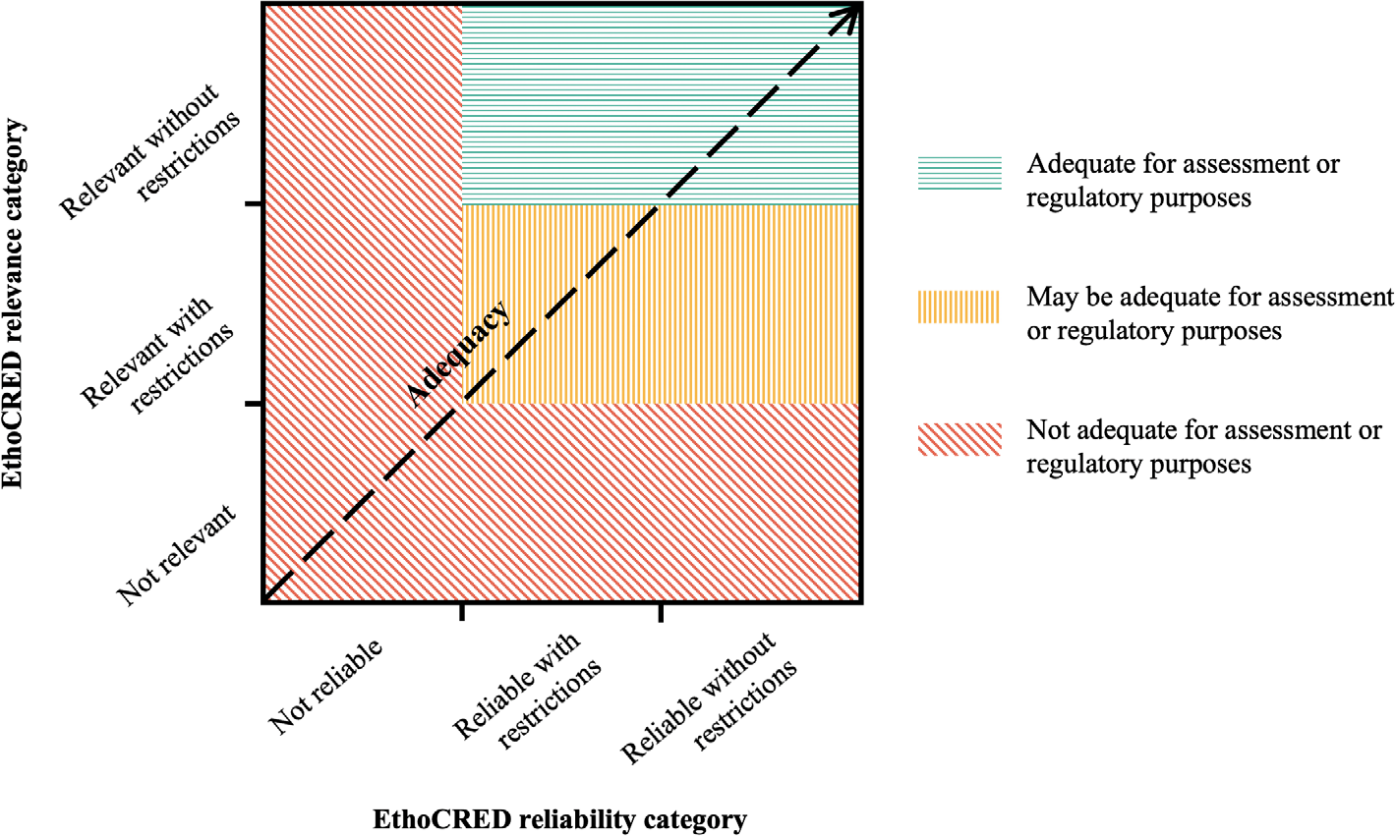
Diagram illustrating an approach for summarising the adequacy of behavioural ecotoxicity data for assessment or regulatory purposes using the EthoCRED relevance and reliability categories. Adapted from Ågerstrand *et al*. (2011), Moermond *et al*. (2016), and Hartmann *et al*. (2017).

The overarching goal of the EthoCRED evaluation method is to increase the use of behavioural studies in environmental hazard and risk assessment. EthoCRED facilitates this change not only by specifying relevance and reliability criteria with which risk assessors and regulators can evaluate behavioural studies but also by providing detailed explanations of each of these criteria. Furthermore, based on the overall conclusion of the relevance and reliability evaluations, study evaluators are able to assign studies to the appropriate relevance category (i.e. relevant without restrictions, relevant with restrictions, not relevant, or not assignable) and reliability category (i.e. reliable without restrictions, reliable with restrictions, not reliable, or not assignable). The overall adequacy of a study to inform assessment or chemical regulation may then be assessed according to the EthoCRED approach summarised in Fig. 2. Using this approach, a study in behavioural ecotoxicology is considered “adequate for assessment or regulatory purposes” if it is reliable without restrictions *or* reliable with restrictions *and* relevant without restrictions. Studies considered adequate for assessment or regulatory purposes could, for example, inform the derivation of PNEC and EQS values for risk assessment purposes. Further, a study “may be adequate for assessment or regulatory purposes” if it is reliable without restrictions *or* reliable with restrictions *and* relevant with restrictions. These studies may, for example, be used as supporting evidence in a risk assessment, or could be used for PNEC derivations in cases where limited data are available. Using the EthoCRED approach, behavioural ecotoxicology studies that are categorised as not relevant *and*/*or* not reliable are considered “not adequate for assessment or regulatory purposes”.

It is important to emphasise that, even though the current paper provides comprehensive guidance accompanying each criterion, use of expert judgment in the evaluation of behavioural ecotoxicity research remains necessary. The assessment of relevance and reliability should not be reduced to a checklist, where the determination of a relevance or reliability category relies solely on the number of criteria met or unmet (Baker, 2015; Moermond *et al*., 2016). Rather, appraising a study's relevance and reliability must be rooted in robust scientific reasoning, meaning that expert judgement should play a central role. For instance, in many cases, whether an EthoCRED relevance or reliability criterion is sufficiently met will depend on the study species tested, given that species can vary greatly in their natural expression of traits like reproductive behaviour, anti‐predator behaviour, and/or learning and cognition (Hager, 2010). Similarly, species vary considerably in their natural expression of anxiety‐like behaviours and resilience to disturbances (Maximino *et al*., 2015), meaning that the evaluation of experimental design choices such as laboratory housing duration upon collection from the wild, or acclimation time before behavioural trials, may be highly dependent on the species used. Moreover, behavioural data may be strengthened through the incorporation of complementary endpoints, such as bioaccumulation of the target chemical(s) in organismal tissues, as well as physiological and molecular biomarkers associated with contaminant exposure (Gunnarsson *et al*., 2019; Matthee *et al*., 2023). Hence, the EthoCRED method does not provide a solution for every possible scenario but, instead, represents a framework through which evaluations can be made with increased reproducibility, transparency, and consistency, and with expert judgement often being necessary.

It is also necessary to highlight that some degree of flexibility may be warranted when implementing the EthoCRED evaluation method. Considering the very limited uptake of behavioural data in hazard and risk assessment to date, the vast majority of studies in this field have not been designed with regulatory purposes in mind. As such, overly rigid application of the EthoCRED evaluation method may lead to the loss of valuable data, with potentially significant consequences for substances with already‐limited data availability. Although such challenges may be unavoidable in the short term, we anticipate that the adoption of the EthoCRED evaluation method and adherence to the EthoCRED reporting recommendations will ultimately enhance the reliability of peer‐reviewed articles. This improvement is expected to make a broader range of behavioural studies accessible for future risk assessments and EQS derivations.

### (2) EthoCRED reporting recommendations

Published scientific papers should include a sufficient level of detail to enable the replication of the reported experiments and findings. In fact, detailed reporting is crucial to the scientific method, allowing fellow researchers to validate and build upon existing work, as well as enabling external parties to evaluate the research. Despite this, peer‐reviewed articles often fall short in providing sufficient information for a comprehensive assessment of the research (Ågerstrand, Breitholtz & Rudén, 2011; Ågerstrand *et al*., 2013). This may be partly due to the space and word limitations of scholarly publication but can also result from complacency, as well as a lack of standardisation of the experimental details that should be reported. Within the field of behavioural ecotoxicology, which is a relatively young discipline that has not yet been integrated into hazard and risk assessment, this issue of insufficient and/or inconsistent reporting is also driven by the fact that researchers may not anticipate that their work will be used in an applied environmental protection context. As a result, despite the fact that many researchers in behavioural ecotoxicology may aspire for their data to be used in this context, they may not be aware of the requirements set by regulatory agencies for inclusion of studies in risk assessments.

The EthoCRED reporting recommendations provide a structured framework to guide the reporting of behavioural ecotoxicity studies. Crucially, these recommendations encourage transparent reporting of details relating to test designs and results, as well as ensuring that at least the minimum amount of information on these elements is available to evaluators. Moreover, the EthoCRED reporting recommendations are expected to simplify the writing process for authors by serving as a structured template that can be followed. Considering that behavioural ecotoxicity studies vary considerably in terms of their experimental design, not only are these reporting recommendations expected to facilitate a simplified and more thorough evaluation for hazard and risk assessment, but by increasing the consistency of reporting they will also make published studies more useful for other researchers for planning and implementing their own experiments. For example, the bioaccumulation and bioconcentration of certain ionisable contaminants can vary based on the pH of the water in which organisms are exposed (Martin *et al*., 2019a), meaning that failing to report pH during waterborne exposure could mislead other researchers as to the effects of these chemicals. Other physicochemical parameters (e.g. temperature, hardness, conductivity, dissolved oxygen concentration) can also modify the accumulation and effects of contaminants, and should therefore be considered and reported, where relevant. As such, improved reporting of behavioural ecotoxicity studies stands to increase reproducibility in the field as a whole.

## VI. CONCLUSIONS

(1) Behavioural analysis represents a sensitive, powerful, and ecologically meaningful means of evaluating the potential environmental impacts of chemical contaminants. Despite this, uptake of behavioural endpoints into hazard and risk assessment to date has been very limited.
(2) In this paper, a group of 35 experts working in the field of behavioural ecotoxicology present the EthoCRED evaluation method for assessing the relevance and reliability of published studies in this field. We hope that this framework will aid risk assessors and regulators in evaluating behavioural studies, and thereby allow these valuable data to be applied to environmental protection. Further, we expect that the EthoCRED reporting recommendations for researchers will increase the transparency and reproducibility of published behavioural ecotoxicity studies, and thereby make these studies more useful in hazard and risk assessments.
(3) Only through concerted and transparent research efforts, and progressive risk assessment approaches, will we be able to make the informed decisions required to reduce the environmental impacts of chemicals.

## VIII. DISCLAIMER

The views expressed in this paper are those of the authors and do not necessarily reflect the view or opinions of their institutions. The mention of trade names or commercial products does not constitute endorsement or recommendation for use. Any use of trade, firm, or product names is for descriptive purposes only and does not imply endorsement by the U.S. Government. This product (article, paper, etc.) has been peer reviewed and approved for publication consistent with USGS Fundamental Science Practices (https://pubs.usgs.gov/circ/1367/). The content is solely the responsibility of the authors and does not necessarily represent the official views of the National Institutes of Health.

## Supporting information


**Appendix S1.** Fig. 1 data collection method and search terms.

**Appendix S2.** Manual for the practical use of the EthoCRED relevance and reliability criteria.

**Appendix S3.** EthoCRED method for evaluating relevance and reliability, to be used together with the accompanying guidance material (.xls spreadsheet).

**Appendix S4.** EthoCRED reporting recommendations, to be used together with the accompanying guidance material (.xls spreadsheet).
