# Supplementary material for: EthoCRED: a framework to guide reporting and evaluation of the relevance and reliability of behavioural ecotoxicity studies
Source: Biol Rev Camb Philos Soc. 2024 Oct 12;100(2):556–85. doi: 10.1111/brv.13154 (PMC11885694; doi:10.1111/brv.13154)
Supplement: Supplementary file 1 — Appendix S1. Fig. 1 data collection method and search terms. [file BRV-100-556-s003.pdf]

Appendix S1. Fig. 1 data collection method and search terms

Data for Fig. 1 were collected from the *Web of Science* Core Collection on 22-07-2024 by M.G. Bertram. Searches for topic terms were carried out in the following fields within each record: title, abstract, keywords, and keywords plus (words or phrases that frequently appear in the titles of an article's references but do not appear in the title of the article itself). Title and abstract screening were not conducted, therefore, the total number of returns does not necessary accurately reflect the total number of relevant articles.

Search string for behavioural ecotoxicology literature: ((TS = (behav* OR personalit* OR courtship* OR "parental care" OR "maternal care" OR "paternal care" OR mating OR "mate choice" OR "mate selection" OR "mate attract*" OR spawn* OR cuckold* OR nest* OR predat* OR antipredat* OR anti-predat* OR escap* OR burrow* OR cryptic OR hiding OR shelter* OR forag* OR feed* OR hunt* OR provision* OR aggress* OR schooli* OR shoal* OR social* OR affiliat* OR defen* OR contest OR dispers* OR migrat* OR swim* OR locomot* OR move* OR "activity level*" OR exploration OR anxiety OR bold* OR scototaxis OR phototaxis OR thigmotaxis OR learn* OR memory OR cognit*)) AND (TS = (animal* OR wildlife OR organism* OR fish* OR amphibia* OR reptile* OR insect* OR invertebrate* OR arthropod* OR echinoderm* OR crustacea* OR mollusc* OR amphipod* OR mammal* OR bird* OR zooplankton* OR daphnia OR zebrafish OR elegans)) AND (TS = (ecotox*)) NOT (TS = ("environment* behav*" NOT "chem* behav*" NOT "part* behav*" NOT "human* behav*" NOT "consum* behav*" NOT "phase behav*" NOT "behav* of chem*" NOT "behav* of contamin*" NOT "behav* of comp*" NOT "behav* of pollut*" NOT "drug discovery" NOT "drug development" NOT "marine corps" NOT "drug design" NOT "essential oil"))) AND (DT==("ARTICLE"))

Search for ecotoxicology literature: TS = ecotox* AND (DT==("ARTICLE"))

Search for publications across all research fields: (PY= (2000-2023)) AND (DT==("ARTICLE"))
