# Supplementary material for: EthoCRED: a framework to guide reporting and evaluation of the relevance and reliability of behavioural ecotoxicity studies
Source: Biol Rev Camb Philos Soc. 2024 Oct 12;100(2):556–85. doi: 10.1111/brv.13154 (PMC11885694; doi:10.1111/brv.13154)
Supplement: Supplementary file 2 — Appendix S2. Manual for the practical use of the EthoCRED relevance and reliability criteria. [file BRV-100-556-s002.pdf]

Appendix S2. Manual for the practical use of the EthoCRED relevance and reliability criteria

EthoCRED: a framework to guide reporting and evaluation of the relevance and reliability of behavioural ecotoxicity studies

Michael G. Bertram[1,2,3,*], Marlene Ågerstrand[4], Eli S.J. Thoré[1,5,6], Joel Allen[7], Sigal Balshine[8], Jack A. Brand[1,9], Bryan W. Brooks[10], ZhiChao Dang[11], Sabine Duquesne[12], Alex T. Ford[13], Frauke Hoffmann[14], Henner Hollert[15], Stefanie Jacob[12], Werner Kloas[16], Nils Klüver[17], Jim Lazorchak[7], Mariana Ledesma[18], Gerd Maack[12], Erin L. Macartney[2,19,20], Jake M. Martin[1,2,21], Steven D. Melvin[22], Marcus Michelangeli[1,23], Silvia Mohr[12], Stephanie Padilla[24], Gregory Pyle[25], Minna Saaristo[26], René Sahm[12,27], Els Smit[11], Jeffery A. Steevens[28], Sanne van den Berg[29], Laura E. Vossen[30], Donald Wlodkowic[31], Bob B.M. Wong[3], Michael Ziegler[32,33] and Tomas Brodin[1]

[1]*Department of Wildlife, Fish, and Environmental Studies, Swedish University of Agricultural Sciences, Skogsmarksgränd 17, Umeå, 907 36, Sweden*
[2]*Department of Zoology, Stockholm University, Svante Arrhenius väg 18b, Stockholm, 114 18, Sweden*
[3]*School of Biological Sciences, Monash University, 25 Rainforest Walk, Melbourne, 3800, Australia*
[4]*Department of Environmental Science, Stockholm University, Svante Arrhenius väg 8c, Stockholm, 114 18, Sweden*
[5]*Laboratory of Adaptive Biodynamics, Research Unit of Environmental and Evolutionary Biology, Institute of Life, Earth, and Environment, University of Namur, Rue de Bruxelles 61, Namur, 5000, Belgium*
[6]*TRANSfarm, Science, Engineering, and Technology Group, KU Leuven, Bijzondereweg 12, Bierbeek, 3360, Belgium*
[7]*Center for Environmental Measurement and Modeling, Office of Research and Development, U.S. EPA, 26 Martin Luther King Drive West, Cincinnati, Ohio, 45268, USA*
[8]*Department of Psychology, Neuroscience, & Behaviour, McMaster University, 1280 Main Street West, Hamilton, Ontario, L8S 4K1, Canada*
[9]*Institute of Zoology, Zoological Society of London, Outer Circle, Regent's Park, London, NW1, 4RY, UK*
[10]*Department of Environmental Science, Baylor University, One Bear Place #97266, Waco, Texas, 76798-7266, USA*
[11]*National Institute for Public Health and the Environment (RIVM), Antonie van Leeuwenhoeklaan 9, Bilthoven, 3721 MA, the Netherlands*
[12]*German Environment Agency (UBA), Wörlitzer Platz 1, Dessau-Roßlau, 06844, Germany*
[13]*Institute of Marine Sciences, School of Biological Sciences, University of Portsmouth, Ferry Road, Portsmouth, PO4 9LY, UK*
[14]*Department of Chemical and Product Safety, The German Federal Institute for Risk Assessment (BfR), Max-Dohrn-Straße 8–10, Berlin, 10589, Germany*
[15]*Goethe University Frankfurt, Max-von-Laue-Straße 13, Frankfurt am Main, 60438, Germany*
[16]*Leibniz-Institute of Freshwater Ecology and Inland Fisheries, Müggelseedamm 310, Berlin, 12587, Germany*
[17]*Helmholtz Centre for Environmental Research (UFZ), Permoserstraße 15, Leipzig, 04318,*

Germany
[18]*Swedish Chemicals Agency (KemI), Löfströms allé 5, Stockholm, 172 66, Sweden*
[19]*Evolution & Ecology Research Centre, School of Biological, Earth & Environmental Sciences, University of New South Wales, Biological Sciences North (D26), Sydney, 2052, Australia*
[20]*Charles Perkins Centre, School of Life and Environmental Sciences, The University of Sydney, John Hopkins Drive, Sydney, 2006, Australia*
[21]*School of Life and Environmental Sciences, Deakin University, 75 Pigdons Road, Waurn Ponds, 3216, Australia*
[22]*Australian Rivers Institute, School of Environment and Science, Griffith University, Edmund Rice Drive, Southport, 4215, Australia*
[23]*School of Environment and Science, Griffith University, 170 Kessels Road, Nathan, 4111, Australia*
[24]*Center for Computational Toxicology and Exposure, Office of Research and Development, U.S. EPA, 109 T.W. Alexander Drive, Durham, North Carolina, 27711, USA*
[25]*Department of Biological Sciences, University of Lethbridge, 4401 University Drive, Lethbridge, Alberta, AB T1K 3M4, Canada*
[26]*Environment Protection Authority Victoria, EPA Science, 2 Terrace Way, Macleod, 3085, Australia*
[27]*Department of Freshwater Ecology in Landscape Planning, University of Kassel, Gottschalkstraße 24, Kassel, 34127, Germany*
[28]*Columbia Environmental Research Center, U.S. Geological Survey (USGS), 4200 New Haven Road, Columbia, Missouri, 65201, USA*
[29]*Wageningen University and Research, P.O. Box 47, Wageningen, 6700 AA, the Netherlands*
[30]*Department of Anatomy, Physiology, and Biochemistry, Swedish University of Agricultural Sciences, Ulls väg 26, Uppsala, 756 51, Sweden*
[31]*The Neurotox Lab, School of Science, RMIT University, 289 McKimmies Road, Melbourne, 3083, Australia*
[32]*Eurofins Aquatic Ecotoxicology GmbH, Eutinger Strasse 24, Niefern-Öschelbronn, 75223, Germany*
[33]*Animal Physiological Ecology, University of Tübingen, Auf der Morgenstelle 5, Tübingen, 72076, Germany*

[*]Author for correspondence (E-mail: michael.bertram@slu.se; Tel.: +46 (0)70 446 90 55).

Table of Contents

Introduction 4

Overview of the process of evaluating study adequacy 4

Step 1: Evaluate relevance 5

Explanation of the EthoCRED relevance criteria 6

Step 2: Assign relevance category 21

Step 3: Evaluate reliability 22

Explanation of the EthoCRED reliability criteria 24

Step 4: Assign reliability category 57

Step 5: Combine relevance and reliability 58

EthoCRED reporting recommendations 59

Disclaimer 64

References 65

Introduction

The EthoCRED relevance and reliability criteria, and the associated guidance materials, were developed to guide risk assessors when evaluating studies in behavioural ecotoxicology for assessment or regulatory purposes, as well as to aid scientists designing experiments. The EthoCRED method provides a structured framework through which risk assessors can thoroughly, consistently, and transparently evaluate behavioural ecotoxicology research.

Overview of the process of evaluating study adequacy

The process by which the EthoCRED method is used to assess the potential adequacy of behavioural ecotoxicity studies to inform assessment and regulatory activities includes both an evaluation of study relevance and an evaluation of study reliability (Fig. S1). These two assessments are then combined, generating an overall evaluation of study adequacy for a specific assessment purpose – according to guidance of the European Chemicals Agency (2011*a*) – which is routinely linked to protection goals. Note that the relevance and reliability assessment need not be performed in a particular sequential order. However, it may be more efficient to start with a relevance assessment, because reliability assessment of non-relevant studies is often redundant.

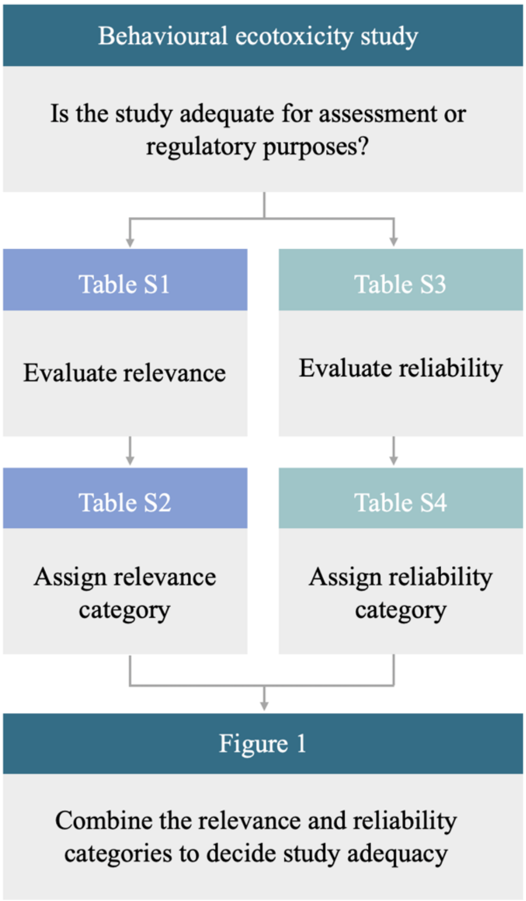

Fig. S1. Diagram of the process of implementing the EthoCRED evaluation method to assess the relevance and reliability of behavioural ecotoxicity studies. The EthoCRED method comprises the following steps: (1) evaluation of relevance, (2) assignment of relevance category, (3) evaluation of reliability, (4) assignment of reliability category, and (5) merging relevance and reliability evaluations into an overall evaluation of study adequacy.

Step 1: Evaluate relevance

Using the EthoCRED method, relevance is evaluated based on 14 relevance criteria (Table S1), which have been adapted from the Criteria for Reporting and Evaluating Ecotoxicity Data (CRED) project (Moermond *et al.*, 2016). Qualitative assessment is used to determine the degree of fulfilment of each criterion. In cases where expert judgement may be required, this has been indicated in the explanatory text accompanying each criterion (see 'Explanation of the EthoCRED relevance criteria'). Although primarily intended for screening studies from the literature, the EthoCRED relevance criteria can also be used as a list of considerations for designing studies that are more likely to be useful for assessment and regulatory activities.

Table S1. EthoCRED relevance criteria[a] for evaluating behavioural ecotoxicity data. The criteria are adapted from the relevance criteria provided in the Criteria for Reporting and Evaluating Ecotoxicity Data (CRED) project (Moermond *et al.*, 2016). Additional guidance on how to interpret the EthoCRED relevance criteria is provided in the text below.

**Number**	**Criterion**
**General information**	
	Before evaluating the test for relevance, indicate the reason for evaluating this study. The relevance of the study might be different for different purposes (e.g. environmental quality criteria derivation, PBT assessment, dossier evaluation for marketing authorisation), also depending on the framework for which the evaluation is requested.
**Biological relevance**	
1	Is the species tested relevant for the compartment (e.g. soil, water) under evaluation?
2*	Are the organisms tested relevant for the tested compound?
3*	Are the reported endpoints appropriate for the intended application or potential regulatory purposes?
4*,†	Are the behaviours quantified relevant for the study species?
5*,†	Are the behaviour-testing arena(s) used relevant to the tested species and the endpoints quantified?
6*	Are the reported endpoints appropriate for the investigated effects or the mode of action of the test substance?
7*	Is the effect relevant on a population level?

8*	Is the magnitude of effect statistically meaningful and biologically relevant for the intended application or potential regulatory purposes (e.g. EC_{10}, EC_{50})?
9*	Are relevant life stages studied?
10*	Are the experimental conditions relevant for the tested species?
11*	If recovery is studied, is this relevant for the framework for which the study is evaluated?
**Exposure relevance**	
12*	Is the tested exposure scenario relevant for the substance?
13*	Is the exposure duration relevant and appropriate for the studied species and endpoints?
14	In case of a formulation, other mixture, salts, or transformation products, is the substance tested representative and relevant for the substance being assessed?

*Criteria discussed in the main article.

†Criteria that specifically relate to behavioural ecotoxicity studies, which are additional to the original CRED criteria.

[a] See main text for further explanation of the EthoCRED criteria and explanatory guidance text on how to interpret the criteria. Note that most criteria are not *per se* critical for the relevance of a study and that this depends strongly on the purpose of the evaluation.

CRED = Criteria for Reporting and Evaluating Ecotoxicity Data; EC_{10}/EC_{50} = 10% and 50% effective concentrations; PBT = persistent, bioaccumulative, and toxic.

Explanation of the EthoCRED relevance criteria (numbers correspond with Table S1)

The guidance provided below comprises a combination of material from the original CRED project (Moermond *et al.*, 2016) and guidance that is specific to the evaluation of behavioural ecotoxicity studies, provided by the EthoCRED project (see main article: Bertram *et al.*, 2024).

1. Is the species tested relevant for the compartment (e.g. soil, water) under evaluation?

CRED: “The species tested should be relevant for the compartment under evaluation. For aquatic ecotoxicity studies, the test species should be relevant for the aquatic compartment. For instance, soil organisms such as nematodes, even when tested in aqueous medium, have lower relevance for aquatic risk assessments. Likewise, terrestrial plants could give information on the sensitivity of plants to a substance but would be of lower relevance in aquatic risk

assessments. Depending on the substance and the framework, saltwater species may or may not be relevant for a freshwater assessment and *vice versa*." (Moermond *et al.*, 2016, p. 1304)

EthoCRED: No EthoCRED-specific guidance for this criterion.

2. Are the organisms tested relevant for the tested compound?

CRED: "Because the purpose of most assessments is to evaluate the potential risks of a substance to sensitive nontarget organisms, care should be given to the representativeness of test species; for example, an insecticide should preferably also be tested on insects, and an antimicrobial substance on cyanobacteria. For a study to be relevant, the test organisms do not necessarily have to be a test species for which an accepted test guideline is available. Information from nonsensitive species can also be relevant, especially when enough data are available to perform a species sensitivity distribution or for hazard assessments. When endocrine-disrupting substances are tested, effects might differ between males and females; for example, one substance mainly affects egg production, and another substance only affects sperm viability and fertility. Thus, a distinction between data on male and female organisms should be made for these kinds of compounds since the relevance of study results could differ between sexes.

Example 11: An insecticide is tested on algae. Although these are not a potentially sensitive species group for this substance, algal growth inhibition is still relevant for PNEC [Predicted No Effect Concentrations] or EQS [Environmental Quality Standards] derivation and is needed for the "base set" of algae, invertebrates, and fish that is required in most regulatory frameworks." (Moermond *et al.*, 2016, p. 1304)

EthoCRED: In behavioural ecotoxicology, it is common that not only standard model species (e.g. zebrafish, *Danio rerio*; African clawed frog, *Xenopus laevis*) but also non-model organisms are studied. Generally, both model and non-model species can be relevant, although studies should ideally provide a sensible rationale for the choice of species with respect to the goal of the research – e.g. a species that is likely to be exposed in the wild, a keystone species, a particularly sensitive/robust species, and/or a suitable/convenient model species to predict impacts in other animals. It is important to note that, particularly for popular model species, various strains may be available that differ in genetic composition (Suurväli *et al.*, 2020). This

includes laboratory strains (which may or may not be genetically uniform or inbred) and wild strains (which are often, but not always, more genetically diverse), and the relevance of the strain(s) used should be evaluated considering the goals of the study. For instance, genetically impoverished laboratory strains may be relevant when high levels of standardisation (and limited among-individual variation) are needed. However, different strains could also differ in their sensitivity and responses, making it difficult to generalise beyond the strain being tested (Aulsebrook, Wong & Hall, 2022). Also, strong selection for optimal performance under laboratory conditions means that behavioural responses of laboratory animals may no longer accurately reflect those of their wild counterparts (e.g. Morgan *et al.*, 2022), and, under those circumstances, non-domesticated strains/populations will often be more suitable for studies that are aimed at predicting behavioural responses of wild populations (Thoré *et al.*, 2021*c*). In the latter case, it becomes important to be mindful that wild populations often experience different evolutionary trajectories (e.g. populations from non-pristine environments may already be adapted to the chemical under investigation), which may lead to differences in how wild animals respond to chemical exposure (Almeida *et al.*, 2021; Brans, Almeida & Fajgenblat, 2021). Therefore, in order to understand species-level responses, it may be necessary to test across multiple strains or populations (see also EthoCRED reliability criterion #9).

Beyond the choice of species and strain/population, other characteristics may also determine the relevance of the study organisms for the tested compound. For instance, males and females often differ in their behavioural baseline (Thoré *et al.*, 2019*a*), their behavioural response to chemical exposure (Bertram *et al.*, 2015; Vossen *et al.*, 2022), or both (Martin *et al.*, 2019*a*). Therefore, studies that do not account for potential differences between sexes may be less relevant, unless justification can be provided – e.g. when it is impossible to sex individuals, as is often the case for juvenile life stages and sexually monomorphic species, when the compound is not expected to have sex-specific effects, and/or when previous research has demonstrated no difference in behavioural baseline between sexes. Besides sex, age or life stage may also determine the behavioural baseline and behavioural effects of chemical exposure and should be justified (see also EthoCRED relevance criterion #9).

3. Are the reported endpoints appropriate for the intended application or potential regulatory purposes?

CRED: "For PNEC and EQS derivation, studies on bioaccumulation may not be relevant. For the determination of an acute EQS, chronic data may be less relevant and *vice versa*." (Moermond *et al.*, 2016, p. 1304)

EthoCRED: In conventional ecotoxicology, apical endpoints such as survival, growth, and reproduction are typically used for assessment or regulatory purposes. In behavioural ecotoxicology, there is a whole range of behavioural traits that are quantifiable, sensitive to chemical exposure, and directly or indirectly linked to traditional apical endpoints and the fitness of animals. For example, a fish swimming erratically at the surface and struggling to maintain its upright position in the water column may be more vulnerable to predation, animals that show impaired courtship and mating behaviours may have lower reproductive success, and animals with reduced mobility may not be able to acquire adequate nutrition, leading to impaired growth and/or survival. Usually, behaviours/behavioural responses are classified under one of five interrelated categories: activity (e.g. activity level, swimming velocity), boldness/shyness/anxiety (e.g. thigmotaxis or wall-hugging behaviour, light–dark preference or scototaxis, gravity-mediated activity or geotaxis), exploration behaviour (e.g. inspection of a novel environment), aggressiveness (e.g. association time with a mirror image), and sociability (e.g. shoaling tendency, group cohesion).

It is important to note that behavioural expression is often driven by various concurrent motivational, cognitive, and emotional mechanisms (Budaev & Brown, 2011), so that classification of behavioural traits may be somewhat arbitrary. In addition, some behaviours are difficult to place within one of the five above-mentioned categories but are nevertheless directly relevant for the fitness of animals, including but not limited to foraging behaviour (e.g. location of food resources, food consumption), antipredator behaviour (e.g. escape, avoidance, vigilance), and reproductive behaviour (e.g. mate choice, courtship, mating, parental care). Therefore, more important than the classification of behaviours, a sensible rationale should be provided as to why the endpoint is meaningful and whether the observed effect sizes are likely to be biologically/ecologically relevant, particularly when researchers investigate behaviours that are not commonly considered. When such justification is missing, expert judgement, informed by information on evolutionary conservation of targets and pathways of relevance to the behavioural measures, is needed to decide the appropriateness of the endpoint and the relevance of the effect size (see also EthoCRED relevance criterion #8).

4. Are the behaviours quantified relevant for the study species?

† Criterion specifically relates to behavioural ecotoxicity studies.

EthoCRED: Measuring the potential effect of exposure to a chemical on an animal's behaviour does not necessarily mean that the observed effect is relevant to the species under investigation. In this regard, a properly designed study to investigate the possible effects of chemicals on animal behaviour must consider the normal behavioural repertoire of the species. For example, evaluating total distance travelled in a sedentary animal may be less relevant to that species' survival than behaviours that do not require travelling significant distances, such as mandible rolling or tail flicks. Care should also be taken when translating a metric used in one species (e.g. diving response in zebrafish) to other species. Consequently, a study is only relevant when the biology and ecology of the studied species are properly factored into the design of the study (see also EthoCRED relevance criterion #10) and the behavioural test(s), which should ideally be motivated (at least in the case of species that are not commonly studied) to aid expert evaluation.

5. Are the behaviour-testing arena(s) used relevant to the tested species and the endpoints quantified?

† Criterion specifically relates to behavioural ecotoxicity studies.

EthoCRED: Careful consideration must be given to the design of the behaviour-testing arena and its relevance to the species and endpoints under investigation. This includes accommodating the basic physiological requirements of the species – which will, in part, be determined by size and life stage – and its natural behavioural tendencies (see also EthoCRED relevance criterion #4). These design considerations include, among others, the dimensions of the arena, temperature, photoperiod, and flow regime (in the case of wind tunnels and water flumes). The importance and impact of each of these factors is largely species- and life-stage specific (see also EthoCRED reliability criterion #17). For instance, testing arenas that are either too small or too large may not allow a species to display its natural behavioural repertoire (e.g. restricted activity of large animals when the arena is too small, unanticipated fright responses of cryptic or prey animals when the arena is too large). In addition to its size, the

shape of the arena must also be appropriate for the species and behavioural endpoint under investigation. For example, a vertical column is more relevant when investigating diel vertical migrations of *Daphnia* species than a shallow rectangular aquarium (Kohler, Parker & Ford, 2018).

Behavioural assessment should be conducted at ambient conditions that are relevant for the tested species and that promote the expression of normal behaviour. For instance, temperature can influence a wide range of behaviours (e.g. several species only display mating behaviour at specific temperatures, such as burbots *Lota lota*, which only spawn when temperatures fall below 4 °C; McPhail & Paragamian, 2000). Also, animals should be tested in a flow regime (i.e. still *versus* moving) that matches the species' natural habitat. For instance, testing a pelagic fish in a fast-moving water flume is less relevant than using a static open-field arena, or *vice versa* for a riverine fish. Similarly, if a study assesses nocturnal behaviours under brightly lit conditions instead of infrared lights, the results may not be relevant.

6. Are the reported endpoints appropriate for the investigated effects or the mode of action of the test substance?

CRED (corresponds with CRED relevance criterion #4): "When a risk assessment is performed for substances with a specific mode of action or a known adverse outcome pathway, studies that assess this particular mode of action or adverse outcome pathway are most relevant. For example, fish biomarkers, vitellogenin concentrations, secondary sex characteristics, and sex ratio are considered to indicate endocrine-disrupting chemicals interfering with estrogens, androgens, and steroidogenesis pathways (OECD, 2012). These biomarkers, however, are not useful for indicating other modes of action such as the glucocorticoid receptor pathway. However, even if the use of a biomarker is not (yet) accepted for use in EQS derivation (Hutchinson *et al.*, 2006), studies on this biomarker can still be listed as supporting information in dossiers, to show the concentration range in which effects may occur." (Moermond *et al.*, 2016, p. 1304)

EthoCRED: Fundamentally the same as CRED, but specifically related to animal behaviour. For example, endocrine-disrupting chemicals that mimic reproductive hormones are most likely to affect reproductive behaviours (reviewed in Söffker & Tyler, 2012; Gore, Holley & Crews, 2018), and anxiolytics may cause prey animals to be excessively bold in the presence of

predators (see Brodin *et al.*, 2013, 2014). Importantly, however, due to the complex mechanistic underpinnings of organismal behaviour, there is also a danger of discounting the potential impact of any given contaminant on a seemingly unrelated behavioural endpoint. For instance, using the two example contaminants above, endocrine-disrupting chemicals have also been shown to alter anxiety-related and anti-predator behaviours (e.g. Reyhanian *et al.*, 2011; Lagesson *et al.*, 2019), while anxiolytics can disrupt mating and reproductive behaviours (e.g. Bertram *et al.*, 2018*a*; Fursdon *et al.*, 2019). For this reason, it is most important that proper justification is provided for the investigated contaminant(s) and the behavioural endpoint(s) reported in a study.

7. Is the effect relevant on a population level?

CRED (corresponds with CRED relevance criterion #5): "Most frameworks consider only traditional test endpoints, such as mortality, growth, and reproduction, which are assumed to be linked to population sustainability. However, nonguideline tests may also report nonguideline or nonstandard endpoints that could be relevant, such as filtration rate and behavioural endpoints. The discussion on which endpoints are population-relevant is ongoing and differs between frameworks. Examples of debated endpoints include blood parameters, general behaviour, swimming speed, gene expression, vitellogenin concentrations, in vitro tests, and coloration." (Moermond *et al.*, 2016, p. 1305)

EthoCRED: Organismal behaviour can have profound population-level consequences through effects on key demographic parameters, such as births, deaths, and migration (Wong & Candolin, 2015; Saaristo *et al.*, 2018). For example, a broad range of reproductive behaviours (e.g. courtship intensity, sexual responsiveness, mating frequency) can directly impact mating outcomes, which, in turn, can affect both the number and quality of offspring that are produced and recruited into the population (Candolin & Wong, 2019; Aulsebrook *et al.*, 2020). Similarly, in species with parental care, the amount of effort invested into offspring (e.g. nest defence, provisioning rates) can also be important (Royle, Smiseth & Kölliker, 2012; Aulsebrook *et al.*, 2020). Likewise, behaviours that affect how well animals are able to acquire resources (e.g. time taken to find food, feeding rates) or respond to predators (e.g. time spent hiding, activity levels) can influence population dynamics through effects on survival (Saaristo *et al.*, 2018).

Most studies in behavioural ecotoxicology involve investigating the behavioural responses of animals in the laboratory, with comparatively fewer studies performed under semi-natural or natural field conditions. When evaluating the relevance of behavioural responses at a population level, it is important to consider both the behavioural endpoint being targeted and the experimental settings in which it is being investigated. For example, standardised laboratory assays, whilst certainly valuable, are sometimes criticised for lacking ecological relevance (e.g. exposing animals to chemicals at concentrations that are several orders of magnitude higher than what is encountered in nature; not accounting for species interactions; lack of variation in natural environmental conditions; Bertram *et al.*, 2022). Another important consideration is whether behavioural effects observed under laboratory conditions are predictive of how animals will respond in the wild (Saaristo *et al.*, 2018). This can be tested by embracing a more integrative approach, involving research performed across multiple scales and levels of ecological complexity (e.g. testing migration of salmon smolts exposed to pharmaceutical pollution both in the laboratory and in a natural river system: Hellström *et al.*, 2016), and aided by an increasingly sophisticated array of experimental tools and technological advances [e.g. high-throughput wildlife tracking systems (Bertram *et al.*, 2022; Nathan *et al.*, 2022)].

8. Is the magnitude of effect statistically meaningful and biologically relevant for the intended application or potential regulatory purposes (e.g. EC_{10}, EC_{50})?

CRED (corresponds with CRED relevance criterion #6): "In a standardised test system with relatively little control variation, minor changes may be statistically significant without necessarily being considered ecologically relevant. Expert judgment is needed to decide if the observed effect is caused by the chemical under investigation, especially when no concentration–response relationship is observed. Please note that if enough data are presented in tables or graphs, additional endpoints may be calculated by the assessor if not reported in the study.

For the derivation of chronic risk limits, EC_{10} [10% effective concentration] and NOEC [No Observable Effect Concentration] values are the preferred type of effect values. However, EC_{50} [50% effective concentration] values can be used if EC_{10} or NOEC values are missing. If in a certain dataset an EC_{50} value from an acute study is lower than the lowest NOEC value from chronic studies, this information is relevant for the risk assessment. For the derivation of

acute risk limits in the European Union, EC_{50} values are preferred and NOEC/EC_{10} values derived from acute studies are less relevant.

Example 12: An NOEC value is available from an acute toxicity study with *Gammarus* sp. This NOEC value is well below the lowest available EC_{50} values for other species and, thus, indicates that *Gammarus* sp. is a very sensitive species. Although in the European Union, EQSs or PNECs for acute toxicity are not based on NOECs, depending on the regulatory framework this information might be used to adjust the assessment factor. In contrast, if the acute NOEC value were higher than EC_{50} values for other species, this would indicate that *Gammarus* sp. is not sensitive to this substance." (Moermond *et al.*, 2016, p. 1305)

EthoCRED: Statistical significance provides a degree of confidence that research findings are supported by the observed data and not due to chance. As such, it can be useful to consider any guidance provided regarding jurisdictional regulatory policy when designing experiments, choosing statistical approaches, and assessing statistical relevance. In behavioural ecotoxicology, statistical significance is important because it is widely recognised that there is considerable behavioural variation both within individuals over time and across individuals (Shaw, 2020). Among other things, the capacity to detect effects of different magnitudes relies on the sample size. In this regard, sample size is one contributing factor determining the probability of rejecting a null hypothesis of no difference between populations when they do not actually differ (i.e. type-I error, a 'false-positive'), or failing to reject a null hypothesis that is actually false in a population (i.e. type-II error, a 'false-negative') (Quinn & Keough, 2002). In other words, sample size is a major determinant of statistical power, which is a measure of the probability that a study will detect a real difference in the data (Mundry, 2010). For more on sample size and statistical significance in behavioural ecotoxicology, see also EthoCRED reliability criterion #26.

Beyond statistical significance, it is important to consider whether the size of an effect (i.e. the magnitude of difference between groups, or the strength of association between variables) is biologically or ecologically relevant. When studies fail to report effect sizes [e.g. Cohen's *d*, odds ratio, Pearson's correlation coefficient (*r*)] or fail to provide sufficient data to infer the effect size (e.g. mean and standard deviation of all groups), the results will be more challenging to interpret and the study therefore loses some relevance. Ideally, to aid expert judgement, it is good practice to make clear why the observed effect size(s) may (or may not) be biologically or ecologically relevant.

It is also worth noting that null-hypothesis significance testing, which is the dominant method for statistical inference in many fields, including (behavioural) ecotoxicology, has received mounting criticism and the field is encouraged to move towards valid alternative methods that are less (or not at all) reliant on reporting of *p* values (e.g. confidence intervals, or credible intervals for Bayesian inference) (Erickson & Rattner, 2020). Hence, studies that do not report *p* values are not automatically irrelevant and evaluating the relevance of the reported results should always be done in light of the statistical method that was used.

9. Are relevant life stages studied?

CRED (corresponds with CRED relevance criterion #7): "The studied life-stage should be appropriate for the experimental design and the purpose of the study. For instance, an early life-stage test with fish embryos or larvae is relevant for investigations of developmental effects but not relevant for investigating effects on reproduction." (Moermond *et al.*, 2016, p. 1305)

EthoCRED: The life stage(s) of tested animals should be reported and appropriate to the experimental design, behaviours analysed, and purpose of the study. For instance, juveniles are typically more sensitive to the effects of chemical exposure than adults (reviewed in Mohammed, 2013). In addition, behavioural expression is plastic and typically develops/changes throughout the course of an animal's life (Thoré, Brendonck & Pinceel, 2020; Thoré *et al.*, 2023*b*). For example, in terms of the behaviours tested, reproductive behaviours (e.g. courtship, mating events) should be studied in sexually active animals (i.e. excluding juveniles, or senescent animals which may no longer reproduce), and antipredator responses (e.g. light–dark preference, C-start response) should be assayed in animals of a sufficient age to exhibit such behaviours. Likewise, sociability (e.g. shoaling tendency, group cohesion) should be tested in animals of an appropriate life stage (e.g. juveniles, sub-adults, adults), given that many species display dissimilar social tendencies and social behaviours at different life stages. For instance, certain fish species shoal only during vulnerable, early life stages, while others live in groups throughout most or all of their lifespan (Ward, Kent & Webster, 2020). Further, amphibian species typically express different behaviours across their life cycle given that their juvenile and adult life stages may inhabit different environments (Johansson, Lederer & Lind, 2010). In studies involving experimental animals that have been collected from the wild, while the exact age may not be known, life stage may be inferred based on morphological

and/or physiological traits that, for example, only manifest at sexual maturity (e.g. gonadosomatic index, secondary sexual characteristics such as mating colouration).

10. Are the experimental conditions relevant for the tested species?

CRED (corresponds with CRED relevance criterion #8): "Not only the species (criterion 1) but also the exposure route and conditions should reflect the compartment under investigation. For instance, freshwater species should be tested in fresh water, and saltwater species should be tested in salt water. If this is not the case, the result may not be relevant. If organisms are exposed through water (e.g. *Chironomus* sp.) and sediment is only needed to provide hiding space or as substrate for eggs, inert alternatives such as glass beads, silica sand, and cotton sheets may be used to prevent interference with the substance in the water phase." (Moermond *et al*., 2016, p. 1305)

EthoCRED: A study can only be considered relevant when the biology and ecology of the tested species are properly factored into the experimental design. This means that, other than the treatment under study, animals should be kept under optimal conditions that are tailored to the species and life stage under investigation, unless when deliberately manipulated (see also EthoCRED reliability criterion #11). For instance, depriving animals of their natural day–night cycle may disrupt their physiology and behaviour (e.g. under constant light exposure; Schligler *et al*., 2021), so that the response of the tested animals to chemical exposure may no longer be comparable to that of their wild counterparts. Likewise, social animals that are kept in isolation, or solitary animals that are kept in groups, may be stressed and/or no longer express their normal behaviour, so that it may become challenging to make meaningful predictions about the impact of chemical exposure in wild animals. A good understanding of the biology and ecology of the tested species is crucial to evaluate whether the experimental conditions are relevant for the species. Further, studies should ideally motivate why the methods are appropriate for the tested species to aid expert judgement, especially in the case of species that are not commonly studied.

11. If recovery is studied, is this relevant for the framework for which the study is evaluated?

CRED (corresponds with CRED relevance criterion #10): "Recovery is not taken into account in most frameworks, the exception being the European authorisation of plant protection products, where results based on recovery are relevant for risk assessments." (Moermond *et al.*, 2016, p. 1305)

EthoCRED: Although recovery is not typically considered in most risk assessment frameworks, it is worth noting that behavioural expression is plastic and may – but does not always – change rapidly when changes in the environment occur (Wong & Candolin, 2015). This means that, while some behavioural changes may be permanent, others could be reversible and return to baseline values. For instance, the antidepressant fluoxetine affected foraging behaviour of hybrid striped bass (*Morone saxatilis* × *M. chrysops*) and this effect could still be observed 6 days after exposure had ceased (Gaworecki & Klaine, 2008), likely due to slow elimination of fluoxetine and its biologically more potent metabolite norfluoxetine from the central nervous system. By contrast, effects of the anxiolytic oxazepam on the swimming activity and boldness of burbots disappeared after a depuration period of 5–7 days (Sundin *et al.*, 2019). This reversibility contrasts with some conventional endpoints in ecotoxicology, such as mortality and certain developmental abnormalities, which are irreversible. Recovery of behavioural traits could in theory also occur during prolonged/continued exposure. For instance, when chronically exposed to selective serotonin reuptake inhibitors such as fluoxetine, homeostatic responses in the brain could revert extracellular serotonin levels to a premedication equilibrium (Andrews *et al.*, 2015) and lead to a return of behavioural expression to pre-treatment levels. As such, results that indicate highly persistent effects of a chemical, even when exposure has ceased, can be used as supporting evidence in hazard and risk assessment. However, this does not necessarily hold in the opposite case (i.e. results that indicate rapid reversal of behavioural effects), not only because even a transient behavioural change may (in)directly have irreversible individual- and population-level consequences (Wong & Candolin, 2015; Saaristo *et al.*, 2018) but also because compensatory responses, such as development of resistance to chemicals, may come at a cost that could still negatively affect animal fitness (Kliot & Ghanim, 2012). Moreover, the assessment of recovery from exposure should be carried out in relation to the exposure profile (i.e. the concentration of a chemical, or chemicals, that the study organisms experienced throughout the exposure period, as a function of time).

12. Is the tested exposure scenario relevant for the substance?

CRED (corresponds with CRED relevance criterion #12): "The exposure scenario includes duration of exposure, exposure concentrations, application of the substance, route of administration, and the exposure schedule (static, semistatic, renewal, flow-through, etc.). Some exposure scenarios may not be relevant for the situation to be assessed within a certain framework. For plant protection products and veterinary pharmaceuticals, for example, the application regime determines the predicted exposure pattern. If the exposure is predicted to be a single peak that declines quickly, a chronic fish study may be less relevant. However, if a substance is present over a longer period of time, because there is continuous discharge into aquatic systems and/or the substance disappears slowly from the water phase, then a chronic fish study may be very relevant." (Moermond *et al*., 2016, p. 1305)

EthoCRED: Adding to the CRED guidance for this criterion, the exposure route of the substance should be appropriate for the study organism and should be justified (e.g. waterborne, airborne, dietary). Further, direct injection of the test substance into animal tissues is less realistic in the context of environmental risk assessment of chemicals (Harris *et al*., 2014), and should therefore be avoided or appropriately justified.

13. Is the exposure duration relevant and appropriate for the studied species and endpoints?

CRED (corresponds with CRED relevance criterion #9): "The exposure time should be in line with the endpoints and the test organism under investigation. For algae, the maximum exposure time is usually 3 d to 4 d; but depending on the test species, 7-d exposure may also be used. Although most guidelines recommend exposure for 96 h for an acute toxicity tests on fish, this does not mean that a 5-d or 10-d test is not relevant. Expert judgment is needed to decide whether a test should be considered acute or chronic. When studying chronic effects, sensitive life-stages should be included or a whole life cycle should be studied." (Moermond *et al*., 2016, p. 1305)

CRED (also corresponds with CRED relevance criterion #13): "Depending on the framework and the purpose of assessment, the exposure scenario may not be relevant for the species tested. For example, exposure for only a few minutes can be relevant to study reproductive effects in fish eggs but may not be relevant to assess acute or chronic effects on adult fish." (Moermond *et al*., 2016, p. 1305)

EthoCRED: Studies investigating the behavioural effects of chemical exposure may be concerned with the immediate effects of short-term (acute) exposure, the effects of continued (chronic) exposure, and/or delayed effects (i.e. those that are not observed until days or weeks after exposure, or epigenetic effects seen in subsequent generations). In contrast to classic ecotoxicology, no guidelines currently exist that define a standard exposure duration for behavioural studies in ecotoxicology, so that various exposure durations may be encountered in the literature, as well as various interpretations of what constitutes acute or short-term exposure *versus* chronic or long-term exposure. Importantly, the relevance and appropriateness of the exposure duration should be evaluated in light of the goal(s) of the study, the properties or environmental occurrence of the chemical compound, the studied endpoints, and the biology (e.g. life cycle) of the tested species. For instance, if the goal of the study is to assess the impact of exposure to a (pseudo-)persistent chemical on an environmentally relevant timescale, a 2-week exposure could be considered relevant in the case of a relatively short-lived species such as *Daphnia*. Furthermore, biologically active chemicals (e.g. neuroactive drugs) may have a therapeutic delay rather than exerting an immediate response, so that acute or short-term exposure may be less relevant, in particular if the compound persists in the environment. For instance, serotonin-reuptake inhibitors (e.g. fluoxetine) may not only act directly through their pharmacological properties but also indirectly by delayed compensatory responses in the brain, which could take several weeks to develop (Andrews *et al.*, 2015), as the substance slowly accumulates in brain tissue to therapeutically active levels.

14. In case of a formulation, other mixture, salts, or transformation products, is the substance tested representative and relevant for the substance being assessed?

CRED (corresponds with CRED relevance criterion #11): "A substance may be tested as a pure active substance or in a formulation. Tests performed with formulations may be of lower

relevance for EQS derivation within the Water Framework Directive and of higher relevance for assessments within the Plant Protection Product framework. For pharmaceuticals, the metabolite excreted by humans or livestock may be more relevant for risk assessment than the parent substance. For unstable substances, it should be known if transformation products are formed and if these transformation products are toxic. If the substance causing the effect is not the substance under investigation, expert judgment is needed to decide on how to deal with the results of the study and the resulting risk assessment." (Moermond *et al.*, 2016, p. 1305)

EthoCRED: No EthoCRED-specific guidance for this criterion.

Step 2: Assign relevance category

Based on the overall conclusion from the relevance evaluation, the appropriate relevance category (as described in Table S2) is assigned to the study.

Table S2. EthoCRED relevance categories. Note that these categories correspond with the original Criteria for Reporting and Evaluating Ecotoxicity Data (CRED) relevance categories outlined by Moermond *et al.* (2016).

Relevance category	Description
Relevant without restrictions	The study is relevant for the purpose for which it is evaluated.
Relevant with restrictions	The study has limited relevance for the purpose for which it is evaluated.
Not relevant	The study is not relevant for the purpose for which it is evaluated.
Not assignable	Studies that do not give sufficient details since the result is presented in abstracts or secondary literature (books, reviews, etc.) or studies for which the documentation is not sufficient for assessment of relevance for one or more vital parameters.

Step 3: Evaluate reliability

Using the EthoCRED method, reliability is evaluated according to 29 reliability criteria (Table S3). These criteria are adapted from the CRED evaluation method (Moermond *et al.*, 2016). Qualitative assessment is used to determine the degree of fulfilment of each criterion. In cases where expert judgement may be required, this is indicated in the explanatory text accompanying each criterion (see 'Explanation of the EthoCRED reliability criteria'). Although primarily intended for screening studies from the literature, the EthoCRED reliability criteria can also be used as a list of considerations for designing studies that are more likely to be useful for assessment and regulatory activities.

Table S3. EthoCRED reliability criteria[a] for evaluating behavioural ecotoxicity data. The criteria are adapted and modified from the reliability criteria provided in the Criteria for Reporting and Evaluating Ecotoxicity Data (CRED) project (Moermond *et al.*, 2016). Additional guidance on how to interpret the EthoCRED reliability criteria is provided in the text below.

**Number**	**Criterion**
**General information**	
	Before evaluating a test, check the physicochemical characteristics of the compound (handbooks/general sources). What is the solubility, log K_{OW}, or pK_a? Is the compound volatile? Does it hydrolyse, photolyse, etc.?
**Test setup**	
1*	Is a guideline method (e.g. OECD/ISO) or modified guideline used?[b]
2*	Is the test performed under good laboratory practice (GLP) conditions?[b]
3*	If applicable, are validity criteria fulfilled (e.g. control survival, growth, activity)?
4*	Are appropriate controls performed (e.g. solvent control, negative and positive controls)?
**Test compound or formulation**	
5	Is the test substance identified by name or CAS number? Are test results reported for the appropriate compound?
6	Is the purity of the test substance reported? Or, is the source of the test substance trustworthy?
7	If a formulation is used or if impurities are present: do other ingredients in the formulation exert an effect? Is the amount of active substance or metabolites in the formulation reported?

Test organism

8* Are the organisms well described (e.g. scientific name, mass, length, growth, age/life stage, strain/clone, sex if appropriate)?

9* Are the test organisms from a trustworthy source and, if relevant, acclimatised to laboratory conditions? Have the organisms not been pre-exposed to the test compound or other unintended stressors?

Exposure conditions

10 Is the experimental system appropriate for the test substance, taking into account its physicochemical characteristics?

11*,† Is the exposure system appropriate for the test organism (e.g. choice of medium or test water, feeding, medium characteristics, temperature, light/dark conditions, pH, ammonia, dissolved oxygen)? Have conditions been kept stable throughout the exposure period?

12 Were exposure concentrations below the limit of water solubility (taking the use of a solvent into account)? If a solvent is used, is the solvent within the appropriate range and is a solvent control included?

13* Is appropriate spacing between exposure concentrations applied?

14 Is the exposure duration defined and appropriate?

15* Are chemical analyses adequate to verify concentrations of the test substance over the duration of the study?

16* Is the biomass loading of the organisms in the test system within the appropriate range (e.g. <1 g/l)?

Assessing biological responses

17*,† Is the behaviour-testing environment appropriate for the experimental organism and research question(s) (e.g. size and shape of trial arenas, time window for testing, avoidance of chemical, visual, and auditory interference)?

18*,† If relevant, was an acclimation period employed before behavioural trials?

19*,† Is the duration of behavioural trials reported?

20*,† For feeding and foraging trials, were animals fed an appropriate amount and at an appropriate time relative to the commencement of behavioural trials? Is the kind and quantity of feed/prey used reported and appropriate?

21*,† In behavioural trials involving a predator, was an appropriate predatory stimulus used (e.g. was an anti-predator response observed in controls?)?

22*,† Were behavioural trials recorded (e.g. video and/or audio recordings)?

23*,†	Was/were the experimenter(s) blind to experimental treatment when conducting and analysing behavioural trials?
24*,†	If relevant, were experimental design elements appropriately randomised (e.g. assignment of animals to treatment groups, treatment type in behavioural trials, behavioural trial type in repeated testing, treatments across arenas in simultaneous testing, potential edge effects)?
25*,†	If animals were repeatedly tested using the same behavioural assay, were habituation effects accounted for?
**Statistical design and analysis**	
26*	Is a sufficient number of replicates used? Is a sufficient number of organisms per replicate used for all controls and test concentrations?
27*	Are appropriate statistical methods used?
28*	Is a concentration–response relationship observed?
29*	Are sufficient data available to check the calculation of endpoints and (if applicable) validity criteria (e.g. control data, raw data, dose–response curves)?

*Criteria discussed in the main article.

†Criteria that specifically relate to behavioural ecotoxicity studies, which are additional to the original CRED criteria.

[a]See main text for further explanation of the EthoCRED criteria and explanatory guidance text on how to interpret the criteria. Please note that most criteria are not *per se* critical for the reliability of a study and that this depends strongly on the compound and/or species tested.

[b]These EthoCRED criteria are of minor importance for study reliability but may support study evaluation.

CAS = Chemical Abstracts Service; CRED = Criteria for Reporting and Evaluating Ecotoxicity Data; ISO = International Organisation for Standardisation; K_{OW} = octanol–water partition coefficient; OECD = Organisation for Economic Co-operation and Development; pK_a = dissociation constant.

Explanation of the EthoCRED reliability criteria (numbers correspond with Table S3)

The guidance provided below comprises a combination of material from the original CRED project (Moermond *et al.*, 2016) and guidance that is specific to the evaluation of behavioural ecotoxicity studies, provided by the EthoCRED project (see main paper: Bertram *et al.*, 2024).

1. Is a guideline method (e.g. OECD/ISO) or modified guideline used?

CRED (corresponds with CRED reliability criterion #1): "Use of a guideline method (OECD [Organisation for Economic Co-operation and Development], ISO [International Organisation for Standardisation], USEPA [US Environmental Protection Agency], or comparable) does not necessarily reflect the reliability of a study, and it should therefore never be a critical criterion. A guideline study may be unreliable if there are flaws in the design, conduct, and/or (statistical) interpretation, or if results give rise to doubt. This may occur when, for instance, exposure conditions are not suitable for the substance under investigation, control mortality is too high or other validity criteria are not met, or when presumed outliers are left out of consideration without proper justification. The criterion is included in the CRED evaluation method to aid with transparent evaluation. Studies using non-guideline methods may be equally reliable as guideline studies, provided enough details on the experimental design and results are presented to assess its reliability." (Moermond *et al.*, 2016, p. 1300)

EthoCRED: Currently, behavioural endpoints are, with a few exceptions, not represented in guideline methods (discussed in Ågerstrand *et al.*, 2020; Ford *et al.*, 2021). Until they are, non-standard studies need to be considered in environmental assessments, including chemical hazard and risk assessments, if behavioural endpoints – which are typically more sensitive than conventional ecotoxicological endpoints (reviewed in Melvin & Wilson, 2013) – are to be represented. Given this general lack of standardised methods for behavioural ecotoxicity testing, the reliability of a behavioural study should not be judged based on whether it is a guideline study or not. Instead, an evaluation of the test design, performance, and data analysis should determine its potential for use. Furthermore, the use of guideline tests that are adapted but not specifically developed for behavioural research may even result in reduced reliability when compared with non-standard studies. This is, for example, the case when factors that may be crucial to a species' behaviour and/or ecology are not taken into consideration.

2. Is the test performed under good laboratory practice (GLP) conditions?

CRED (corresponds with CRED reliability criterion #2): "Good laboratory practice is a data quality system that requires adequate documentation of the experimental process. Laboratories working under good laboratory practice often use standardised methods, both for performing the test and for documenting the results. Good laboratory practice does not, however, reflect the actual reliability of a study and should therefore never be a critical criterion. It is included

in the CRED evaluation method to aid with transparent evaluation." (Moermond *et al.*, 2016, p. 1300)

EthoCRED: Good laboratory practice promotes reproducibility and transparency but is not in itself a guarantee of high study reliability. Therefore, good laboratory practice should not be used as an argument to select or deselect non-standard studies investigating behavioural effects (Moermond *et al.*, 2016).

3. If applicable, are validity criteria fulfilled (e.g. control survival, growth, activity)?

CRED (corresponds with CRED reliability criterion #3): "In most test guidelines, validity criteria are provided to determine the validity of the test results. For instance, OECD guideline 201 on algal toxicity requires exponential growth in the controls and specifies criteria for the variation in growth rate within and between control replicates. For the *Daphnia* acute toxicity study, the validity criteria in the OECD 202 guideline include control mortality and oxygen concentrations. Besides this, control organisms should be from the same population as the treatment group(s), variability in the controls should fall within the same range as historical data, and attention should be given to natural fluctuations in results, such as fluctuations attributable to age of the animals or seasonal influences. If a non-guideline test is performed with a guideline species, validity criteria as described in the relevant guideline should be met. If non-guideline species are used, expert judgment is needed to assess whether the test organism resembles the guideline test species enough to apply guideline validity criteria. Otherwise, expert judgment is needed to decide if control survival and/or other parameters are within the range of what is normal for the species and that other confounding (stress) factors can be ruled out. For guideline test species, however, complying with guideline criteria for validity (e.g. control survival, growth) is critical for a study to be reliable." (Moermond *et al.*, 2016, p. 1300)

EthoCRED: This criterion particularly relates to studies that are conducted according to (modified) guidelines that include validity criteria. Behavioural studies are, with a few exceptions, performed in non-standard settings that do not have predefined validity criteria. In the absence of validity criteria for a study, validity criteria from a guideline study may be used for guideline test species – although, such validity criteria may also not be entirely relevant or possible to achieve given the often-specialised experimental design and logistical requirements involved in behavioural ecotoxicity studies. In cases where modified guidelines are used,

resulting in irrelevant or impossible validity criteria, expert judgement is needed to determine the potential impact of confounding factors. Importantly, any study with excess mortality in the control treatment(s) likely indicates an issue with experimental conditions or health of the study organisms. For a general discussion of issues relating to this criterion, see Moermond *et al.* (2016).

4. Are appropriate controls performed (e.g. solvent control, negative and positive controls)?

CRED (corresponds with CRED reliability criterion #4): "The decision of which controls to use depends on the test substance and/or the guideline applied. Next to the 'normal' negative controls (no solvent, no test substance), solvent controls need to be tested in all cases where a solvent is used. The concentration of solvent in the solvent control should be the same as the highest concentration used in the test treatments, and mortality in the solvent controls should preferably not differ significantly from that in the nonsolvent controls. The study is 'not reliable' (R3) if, for instance, a solvent is used but no solvent control is tested or the solvent concentration in the control treatment is too low. If the mortality in the solvent control is higher than that in the nonsolvent control, statistics should be based on the solvent control. Expert judgment is needed to decide when the mortality in the solvent control is too high, especially when it is still within the validity criteria of the test.

In some cases, a positive control (with a reference substance) is tested. The lack of a positive control decreases the reliability of a study only if a positive control is requested in the test guideline. On the other side, use of a positive control might increase confidence regarding the reliability of study results." (Moermond *et al.*, 2016, p. 1300)

EthoCRED: Sufficient, appropriate controls are necessary for a study to be considered reliable. Typically, the control group receives no treatment but otherwise follows the exact same procedures as the vehicle and treatment groups, to enable direct comparisons. This means that, other than the treatment itself, all other procedures should be standardised (or randomised, when appropriate) across conditions to prevent systematic differences in behaviour due to factors other than the treatment under investigation. Examples of these potentially confounding variables include the age and/or life stage of animals (Peterson *et al.*, 2017), the order in which individuals are allocated to experimental groups (Härkönen *et al.*, 2016), the timing of behavioural observations with regard to potential daily fluctuations in behaviour (Thoré,

Brendonck & Pinceel, 2021*a*), and the order of behavioural assays in cases where multiple behaviours are scored (Bell, 2013) (see also EthoCRED reliability criterion #24).

Expert judgement is needed to decide if mortality and behaviour of control animals falls within a range that can reasonably be expected. When relevant, studies should report on the number of mortalities in each treatment and how to interpret excess mortality or unexpected behaviour of control animals. However, behavioural ecotoxicity studies that do not report mortality are not necessarily unreliable, given that behavioural studies often use sublethal exposure concentrations, meaning that mortality has conventionally been reported less often because it is not an expected outcome of exposure. It should also be noted that, in behavioural studies, data points may be deleted from the final data set and considered as missing data in the case of errors during behavioural data collection (e.g. technical issues that may have affected behaviour or rendered recordings unusable). This does not threaten reliability as long as the final sample size is sufficiently large to establish the baseline variability in behaviour (Paull *et al.*, 2008; Harris *et al.*, 2014). However, reasons for such missing data should be reported and justified.

Other controls (e.g. positive controls, placebo controls) may be useful in some cases (e.g. Tanoue *et al.*, 2019) but are not strictly required. For example, when testing known monoamine disruptors, positive controls such as serotonin or dopamine have been used along with their pharmacological agonists (e.g. Bringolf *et al.*, 2010). Studies that make use of a solvent or vehicle to administer the chemical under investigation should include an appropriate solvent/vehicle control – i.e. animals that are treated with the solvent/vehicle alone at a concentration equal to that used in the primary experiment, with all other methods being equal. Statistical analyses should use the solvent/vehicle control as a benchmark of comparison (Harris *et al.*, 2014). Lack of a solvent/vehicle control can be justified under some circumstances, for instance if historical data show no impact of the solvent/vehicle on the species/population under investigation at the administered dose, or if ethical and/or logistical constraints limit the number of test animals.

5. Is the test substance identified by name or CAS number? Are test results reported for the appropriate compound?

CRED (corresponds with CRED reliability criterion #5): "It is essential to know which substance was tested. If a salt was tested, for example, information on the type of salt and how results are reported (e.g. as salt or as its positively or negatively charged ions, including or

excluding waters of hydration as ·H_2O) is needed. The only exception for this is when results are expressed in molarities instead of grams per litre. In that case, it does not matter if results are expressed as salt or base; although it remains necessary to know which salt is tested, because different counter-ions may give different results, or if a salt contains more than one of the same ion. The lack of a CAS number does not decrease the reliability of the study since it can often be retrieved easily from the Internet or other sources if the tested substance is clearly identified. If a formulation is tested, it is necessary to know all the relevant components of the formulation (see also [CRED] criterion #7).

Example 4: A test with metformin hydrochloride is performed. Values for the no-observed-effect concentration (NOEC) and 50% effect concentration (EC_{50}) are tabulated for "metformin," but it is not specifically mentioned if results are reported for the metformin base or the salt. If metformin hydrochloride was the tested substance, the toxicity values for metformin base would be lower. This decreases the reliability of the study, although results may still be used as supporting evidence in risk assessment." (Moermond *et al.*, 2016, pp. 1300–1301)

EthoCRED: No EthoCRED-specific guidance for this criterion.

6. Is the purity of the test substance reported? Or, is the source of the test substance trustworthy?

CRED (corresponds with CRED reliability criterion #6): "The purity of the substance and/or the source of the substance should be reported and reliable (e.g. a known supplier). Generally, a substance should have a purity of 80% or higher, unless it is known that the impurities do not cause toxic effects and do not influence the toxicity of the substance of interest. When the purity of the substance is <90%, the nominal test results should be corrected for purity (European Commission, 2011). Although the OECD guidelines only refer to a 'suitable purity', the overall reliability of a study that uses a low-purity substance should be lowered. However, when the purity is not known but actual test concentrations are measured, this criterion becomes less important." (Moermond *et al.*, 2016, p. 1301)

EthoCRED: No EthoCRED-specific guidance for this criterion.

7. If a formulation is used or if impurities are present: do other ingredients in the

formulation exert an effect? Is the amount of active substance or metabolites in the formulation reported?

CRED (corresponds with CRED reliability criterion #7): "If a formulation is used for testing a specific compound (e.g. when testing plant protection products), the other constituents of the formulation should be known and/or it should be clear that these other constituents have no ecotoxicological effects. For a study to be reliable, the amount of active substance in the formulation should be known, and it should be clear that the results are (or can be) expressed in terms of active substance. It should be taken into account that certain 'inert' ingredients of formulations can exert biological effects, because coformulants and solvents in formulations may significantly increase or decrease the toxicity of the active substance and there is some difficulty in predicting which type of formulations are critical in terms of such interactions (European Commission, 2002). However, this information might be confidential and, for that reason, not known. If this is the case, the reliability should be lowered. In contrast to this, when the formulation itself (and not the active ingredient only) is evaluated for hazard or risk assessment, it may be enough information to have the exact name of the formulation, including formulation strength, and results may be expressed in amounts of formulation." (Moermond *et al.*, 2016, p. 1301)

EthoCRED: No EthoCRED-specific guidance for this criterion.

8. Are the organisms well described (e.g. scientific name, mass, length, growth, age/life stage, strain/clone, sex if appropriate)?

CRED (corresponds with CRED reliability criterion #8): "When assessing reliability, it is essential to know which organisms were used in the test. At a minimum, the name and information on age or life-stage should be known for a study to be reliable. Other information such as weight, length, or strain/clone is in most cases not essential for the reliability assessment, but it may increase the confidence in the study. When examining hormonal substances, the sex of the organisms (e.g. when testing fish) may influence the results, and this information should thus be known.

Example 5: When performing a test with fish, larvae and juveniles are often more sensitive than adults. Thus, when evaluating the test or when comparing results from different tests, knowledge on the life-stages tested may be important." (Moermond *et al.*, 2016, p. 1301)

EthoCRED: As well as the traits specified in the CRED criteria, all of which can be associated with organismal behaviour, additional traits can influence behaviour and should therefore be specified where relevant. For instance, in assays of reproductive behaviour, the reproductive status (e.g. virgin, gravid, or non-virgin) of the organisms under investigation should be described, given that reproductive status can influence reproductive behaviour and mating outcomes (e.g. Guevara-Fiore, Skinner & Watt, 2009; Richardson & Zuk, 2023). Further, in assays involving interactions between multiple species (e.g. competitive or predator–prey interactions), it should be described whether the species under investigation co-occur naturally in the environment (or may have experienced any previous encounters), because organisms are likely to behave differently when presented with a novel *versus* a familiar competitor, predator, or prey species (discussed in Sih *et al.*, 2010; Ehlman, Trimmer & Sih, 2019).

In addition, the sex of experimental organisms should ideally be reported given that, in many species, the sexes exhibit distinctive behavioural repertoires and/or differ in the extent of expression of behaviours. The sexes, and their behavioural profiles, may therefore be differentially vulnerable to exposure to contaminants (e.g. Bertram *et al.*, 2015; Martin *et al.*, 2019*a*; Thoré, Brendonck & Pinceel, 2021*b*; Vossen *et al.*, 2022). Potential sex differences in exposure can be accounted for by testing for potential behavioural changes in each sex separately, or by incorporating sex as a covariate in statistical models. Importantly, not accounting for, or reporting, sex does not automatically make a study unreliable, although justification should be given for why sex was not considered (e.g. the behaviour under investigation is known to be similarly expressed by males and females, or sex cannot be determined at a given life stage).

9. Are the test organisms from a trustworthy source and, if relevant, acclimatised to laboratory conditions? Have the organisms not been pre-exposed to the test compound or other unintended stressors?

CRED (corresponds with CRED reliability criterion #9): "The source of the test organisms should be known and trusted, and the place of origin should be described for field-collected organisms. Test organisms should be healthy and acclimatised to test conditions (e.g. water

type, temperature) to avoid any unintentional stress caused by a change in conditions. In addition, organisms should not be stressed by test conditions (except when this is part of the research question) or by other (unintended) stressors. When such stress is reflected in high control mortality, the study becomes unreliable. Results of toxicity tests with field-collected test organisms may be biased because of community adaptation to toxic stress if pre-exposure to the test substance has occurred (Blanck, Wängberg & Molander, 1988). If this is the case, the study becomes unreliable." (Moermond *et al.*, 2016, p. 1301)

EthoCRED: Studies in behavioural ecotoxicology often use test organisms from a variety of different backgrounds, ranging from laboratory strains to outbred strains (e.g. crosses between laboratory strains and specimens from pet stores), and field-collected animals. Apart from differences in genetic diversity among strains or populations, potential variation in historical exposure or handling means that organisms may already be adapted to some stressor(s) (Almeida *et al.*, 2021) and/or that there could be confounding experiential/maternal effects (Bell, 2013). For instance, the behavioural response of animals adapted to a specific chemical may not reliably reflect that of specimens without such a history of exposure (see Hamilton *et al.*, 2017). Further, long-established laboratory strains that have partially or completely lost their antipredator defence mechanisms may not be appropriate for use in antipredator trials (see Vossen *et al.*, 2020). Still, the availability of different strains or populations with different backgrounds allows for targeted research and may lead to a more robust overall conclusion on the environmental hazards posed by chemicals. Ideally, studies should provide a sufficient background description of the test organisms to facilitate expert judgement on the suitability of a particular strain or population (see EthoCRED reliability criterion #8), although a detailed account may not always be possible, particularly in the case of specimens from pet stores or field-collected animals. Such a background description is therefore not a strict requirement, provided that all experimental groups share the same history and that the natural variability in behaviour is known (e.g. through the use of appropriate controls, see EthoCRED reliability criterion #4). However, providing no information on background conditions, such as a lack of samples taken to ensure the absence of contamination at the collection site(s) of animals from the field, does limit the reliability of a study.

Regardless of their origin, experimental animals should be healthy (e.g. with regard to parasite or pathogen load, unless this is part of the research question) and acclimated to the testing environment (e.g. housing conditions) to avoid stress that is associated with changing environmental conditions other than the treatment under study. Such unintended stress may be

noticeable in mortality or aberrant behaviour of control animals and may render a study unreliable when not controlled for (see EthoCRED reliability criterion #4). Acclimation periods are very important in behavioural toxicity testing, for example, some species have circadian (or circatidal) rhythms that can take time to adjust to a laboratory setting. Experimenters must therefore be mindful of the time it takes for these rhythms to adjust to laboratory conditions, or fix/adjust daily recording times accordingly (see Thoré *et al.*, 2023*a*). Likewise, in some instances, the longer specimens are removed from the wild, the more they may have habituated to laboratory conditions and the less 'natural/normal' they may behave. The consequences of this are endpoint dependent and thus require a good understanding of the species' baseline behaviours.

10. Is the experimental system appropriate for the test substance, taking into account its physicochemical characteristics?

CRED (corresponds with CRED reliability criterion #10): "Most test guidelines prescribe or give recommendations for the experimental system but allow for flexibility with respect to the actual design. Some requirements depend on the substance and/or organism used. The demands of the appropriate test guidelines should be followed as closely as possible, also for nonguideline test organisms. For instance, the test vessel should preferably be made of glass, but this demand is more important for some substances than for others. When hydrophobic substances are tested in paper cups or plastic containers, for example, the study becomes unreliable because of sorption of the substance to the test vessel.

Static systems may be appropriate for short-term tests with stable substances. However, static systems are usually not appropriate for long-term exposure. Open systems may be used for most substances, but volatile substances need to be tested in a closed test system for the study to be reliable, unless chemical analyses show that volatilisation has not occurred during the experiment. Regular analysis of test concentrations can confirm maintenance of exposure concentrations during the test or be used to calculate actual exposure if concentrations declined. If test concentrations have not been stable during the test or no measurements were performed, it should be clear from the study report that all possible measures have been taken to avoid loss. If this is the case, the study can still be reliable. If not, the study is 'not reliable' (R3); or, if not enough details are provided, the study is 'not assignable' (R4). As indicated before [see Moermond *et al*. (2016)], this criterion may not be applicable to substances that are known to be stable in solution.

Example 6: Unfiltered natural water is used for an acute *Daphnia* toxicity study to test a substance with log octanol–water partition coefficient (K_{OW}) of 4.2. Exposure concentrations are measured in unfiltered water. No information is provided on the amount of particulate matter in the water. Because of the high sorption of the substance, it can be assumed that a significant amount of the substance sorbs to the particulate matter. For many substances, it is assumed that the toxicological effect is caused by the dissolved fraction. Thus, the actual exposure concentration is not known, and the study is less reliable. In contrast, if dissolved concentrations are measured and the total organic carbon is below 2 mg/L (OECD 202 requirement), the study is reliable." (Moermond *et al.*, 2016, p. 1301)

EthoCRED: No EthoCRED-specific guidance for this criterion.

11. Is the exposure system appropriate for the test organism (e.g. choice of medium or test water, feeding, medium characteristics, temperature, light/dark conditions, pH, ammonia, dissolved oxygen)? Have conditions been kept stable throughout the exposure period?

† Criterion specifically relates to behavioural ecotoxicity studies.

EthoCRED: The exposure system must be appropriate for the test organism for a study to be considered reliable. This means that, other than the chemical treatment under study, animals should be kept under optimal conditions throughout the experiment (unless this is part of the research question). Optimal conditions are often species- and life-stage specific (Näslund & Johnsson, 2014; Thoré *et al.*, 2020), so sufficient description of the test environment should be provided to facilitate expert judgement. Factors of interest include, but are not restricted to, dimensions of the housing environment (see EthoCRED reliability criterion #16), temperature, light/dark conditions (e.g. photoperiod, spectrum, light intensity) and, for aquatic organisms, water chemistry (e.g. electrical conductivity, pH, oxygen level), all of which may affect how animals behave and respond to chemical exposure. Furthermore, for tests on aquatic species, water quality measures (e.g. ammonia and nitrite levels) should be kept within appropriate ranges. Studies making use of physical enrichment – i.e. any physical complexity, such as substrate or refuges added to housing containers – should report sufficient characteristics (such as dimensions, ecological rationale, timing of enrichment, amount, inputs, and lighting; see Jones, Webster & Salvanes, 2021), and the social environment in which the animals are housed should be detailed, and justified, with regard to the number/density of conspecifics and the

composition of groups (e.g. sex, age classes; see Martin & McCallum, 2021). Apart from being optimal, all of these conditions should be stable throughout the experiment with the notable exception of studies that deliberately use fluctuating environmental conditions to mimic natural conditions, such as daily temperature fluctuations (Verheyen, Delnat & Stoks, 2019). Stress related to suboptimal conditions of the exposure system, other than the treatment under study, may be noticeable in mortality or aberrant behaviour of control animals, and may render a study unreliable, especially if appropriate controls are not used (see EthoCRED reliability criterion #4).

It is also worth noting that in studies conducted under semi-natural (e.g. mesocosms, enclosures) or natural conditions (e.g. whole-lake exposures, field exposures), environmental conditions often cannot be strictly controlled, if at all. Given that ecotoxicology seeks to understand the effects of contaminants in the real world, this variability certainly does not detract from a study's reliability, although ideally it should be accounted for in the experimental design and statistical analysis where appropriate (e.g. temperature and/or light conditions may be included as covariates when modelling behavioural changes over time). These experimental design decisions include, where relevant, the selection of appropriate 'control' sites for field studies to minimise differences in biotic and abiotic conditions with regard to 'experimental' sites.

In contrast to acute toxicity studies in which animals are typically not fed, behavioural ecotoxicology studies often make use of chronic exposure to sub-lethal concentrations, during which feeding is necessary. Feeding (including the type, amount, and frequency of food provided) should be appropriate for the species and life stage under investigation, and any excess food should be removed after feeding to avoid decreased bioavailability of the test substance (due to sorption), and to maintain good quality of the medium. Likewise, the frequency and method of cleaning the housing environment(s) should be appropriate and reported (e.g. frequency and proportion of water and treatment renewals in aquatic studies). In this regard, cleaning should be sufficient to maintain good quality of the medium and substance concentration, while not imposing any more stress on the animals than is absolutely necessary, and cleaning should be consistent across all (exposed and unexposed) treatment groups.

12. Were exposure concentrations below the limit of water solubility (taking the use of a solvent into account)? If a solvent is used, is the solvent within the appropriate range and is a solvent control included?

CRED (corresponds with CRED reliability criterion #12): "If substances are tested at concentrations below the water solubility, the test can be assumed to be reliable. Depending on the uncertainty in the estimate of the water solubility, results of tests performed above the estimated water solubility may be reliable as well. Expert judgment should be used here. Reports of precipitates may indicate that the solubility was exceeded. In this case, test results are less reliable since actual concentrations do not equal nominal concentrations.

Results from a test in which a substance was tested at nominal concentrations 10 times higher than the solubility should normally be regarded as 'not reliable' (R3) (Mensink, Smith & Montforts, 2008). For tests where this is not the case, the reliability should be subject to expert judgment.

Solvents may be used to prepare stock solutions. It is, however, usually not advised that solvents be added directly to the test vessels to enhance solubility. Solvents should not be toxic to the tested species at the tested concentrations (European Chemicals Agency, 2008). According to several OECD guidelines, the concentration of solvents should not exceed 0.01%. The highest solvent concentration used should be reflected in a solvent control (see also [CRED] criterion #5)." (Moermond *et al.*, 2016, p. 1302)

EthoCRED: No EthoCRED-specific guidance for this criterion.

13. Is appropriate spacing between exposure concentrations applied?

CRED (corresponds with CRED reliability criterion #13): "When spacing between test concentrations is too large, the results are not reliable, especially when deriving a NOEC value. A scaling factor of 3.2 (=√10) is often recommended. As a rule of thumb, a maximum scaling factor of 10 should be applied. Performing a range-finding test may help in determining the right exposure concentrations, and as a result the necessary spacing between exposure concentrations may be reduced.

Example 8: A limit study is performed at 2 concentrations: 0.01 mg/L and 1 mg/L. No effect was observed at 0.01 mg/L, and all organisms were dead at 1 mg/L. The NOEC value could thus have been 0.01 mg/L but also 0.1 mg/L or 0.25 mg/L. Because of this large spacing between the 2 concentrations and the low number of concentrations tested, it is not known what the actual effect concentration is. Thus, no reliable NOEC, EC_{50}, or lowest-observed-effect concentration values can be derived from this study. However, the study may be used as

supportive evidence, for example, to demonstrate the (in)sensitivity of the particular species in relation to other data." (Moermond *et al.*, 2016, p. 1302)

EthoCRED: In ecotoxicology, it is common practice to characterise the dose–response of a substance, which requires a minimum of three to five exposure concentrations. However, it is always advisable to include more experimental treatment levels (OECD, 2006). When a sigmoidal (monotonic) curve emerges, a range of toxicity parameters [e.g. 50% lethal concentration/50% effective concentration (LC_{50}/EC_{50}), No Observable Effect Concentration (NOEC), Lowest Observable Effect Concentration (LOEC), benchmark dose calculations] can be calculated for use in environmental risk assessments (Harris *et al.*, 2014). These parameters cannot be accurately calculated when the spacing between test concentrations is too small or too large, and it may therefore be necessary to perform an *a priori* range-finding test to determine the necessary number of, and spacing between, exposure concentrations. In this regard, a scaling factor of 3.2 is typically recommended (with an upper bound of 10 as a rule of thumb; Moermond *et al.*, 2016).

Importantly, while it is recommended to include such an exposure gradient when designing ecotoxicological studies, experiments in behavioural ecotoxicology are often more logistically complex than conventional ecotoxicity studies and may also face ethical constraints that limit the number of test animals and/or experimental treatments. It is therefore common, and acceptable, for studies in behavioural ecotoxicology to comprise just one or two exposure treatments, in addition to appropriate controls. This is acceptable provided that the exposure concentrations are relevant and justified – i.e. to demonstrate the absence or presence of effects at a certain concentration, or to characterise potential behavioural effects at an environmentally relevant dose. However, it is key for studies that are not designed to establish a dose–response relationship to abstain from making dose–response claims. When a non-monotonic dose–response relationship emerges but the number of tested concentrations is limited due to logistical constraints, it becomes more important that those fewer concentrations producing non-monotonic curves are repeatable and not spurious artefacts. More broadly, considering that changes to animal behaviour after sub-lethal exposure to contaminants can elicit lethal outcomes – e.g. animals exposed to anxiolytic drugs that exhibit impaired anti-predator behaviour and are therefore more likely to be consumed (e.g. Brodin *et al.*, 2013; Martin *et al.*, 2017) – it is essential for hormesis and other non-linear dose–response relationships to be given proper credence when assessing behavioural studies. For a recent discussion of the importance of subthreshold effects in regulatory risk assessment, see Agathokleous *et al.* (2022). Also see

EthoCRED reliability criterion #28 for further discussion of dose–response relationships.

14. Is the exposure duration defined and appropriate?

CRED (corresponds with CRED reliability criterion #14): "The exposure duration should be defined for a study to be reliable. Especially when results from different studies with the same test species are compared, it is necessary to know if the exposure durations were similar. The ideal test duration depends on the test organism used and the endpoint under investigation. Sometimes the exposure is very short (e.g. 1 d), whereas effects are not observed until days or weeks after exposure (delayed effects). Results should then be expressed in terms of the actual exposure duration and not in terms of the duration of the entire experiment, although the delay of the effects should be clearly mentioned when summarising the results to enable comparison with other studies in which the observation of effects was stopped immediately after exposure." (Moermond *et al.*, 2016, p. 1302)

EthoCRED: No EthoCRED-specific guidance for this criterion.

15. Are chemical analyses adequate to verify concentrations of the test substance over the duration of the study?

CRED (corresponds with CRED reliability criterion #15): "It is important to know the actual exposure concentrations, and it should be clear if the reported concentrations are initial or final concentrations, whether they are mean or geometric mean concentrations, and which of these concentrations are used to calculate the effect concentrations. In some cases, such as acute toxicity tests or semistatic (renewal) chronic tests with a stable substance, nominal concentrations without measurements can be acceptable. A static or semistatic acute study with a stable substance (information on stability should then be available from other experimental work or from physicochemical characteristics) may be reliable if no measurements are performed; but in all other cases, the exposure concentrations should be verified by analytical measurements. Analysis of the concentration at the beginning of the test may be enough, depending on the substance and the test system, but measurements usually should be performed at least at the beginning and the end of the experiment. There should be no major loss as a result of degradation, photolysis, volatilisation, hydrolysis, or adsorption to glass or other equipment. During the experiment, the test concentration should be close to nominal (80–120%), especially

when possible loss mechanisms are unknown, and the test design should be adequate to maintain this concentration. If the loss of test substance is higher than 20%, it should be investigated whether this is caused by insufficient performance of the test (in which case the reliability is reduced) or by other loss processes. Since these loss processes may be caused by the intrinsic properties of a substance, it may be impossible to avoid them. In case hydrolysis may have occurred, calculations could be performed by a quantitative structure–activity relationship specialist if experimental data on hydrolysis are missing. If test concentrations deviate more than 20% from nominal, it should be clear that all possible measures have been taken to maintain test concentrations (e.g. renewal, flow-through system). In this case, when the experiment is assessed to have been performed in a technically adequate way, the test could be considered "reliable with restrictions" (R2) (Mensink, Smith & Montforts, 2008). Reported test results should then be based on measured concentrations, preferably as time-weighted averages. However, it should be noted if metabolites were present, and expert judgment is needed to decide whether or not it is most suitable to express effects using the concentrations of the parent substance.

The method used to perform chemical analyses should preferably be reported. If the limit of detection and recovery efficiency of the method used are reported, the reliability of the study increases. However, lack of information on recovery and limit of detection does not make the study unreliable." (Moermond *et al.*, 2016, pp. 1302–1303)

EthoCRED: It should be clearly described whether exposure to chemicals occurs before or during the behavioural evaluation, or both. In all cases, the original CRED criterion applies to the exposure medium. The reliability of a study may further improve when the uptake of the chemical(s) into the tissues of the exposed organisms is measured, in particular when the compound is known to bioaccumulate and if its accumulation in a specific tissue is expected to result in a behavioural effect (e.g. psychoactive or neurotoxic compounds accumulating in the brain/nervous system). For small organisms, where it may not be possible or practical to analyse accumulation of the compound in specific target tissues, whole-body or pooled samples are acceptable in order to meet minimum biomass requirements for the analytical method that is employed.

Behavioural ecotoxicity experiments are often conducted at a much larger scale than typical ecotoxicity tests, in terms of the size and/or volume of the exposure arena(s) and the number of animals under investigation, in order to emulate natural conditions more closely.

These experiments may also include natural substrates or environmental enrichment features that are not present in traditional ecotoxicity experiments. In order to achieve a higher degree of ecological realism of the exposure, there is a trade-off with precise knowledge of exposure concentrations. For example, some exposure substances can partition between two or more compartments, such as water and sediment, affecting their bioavailability such that benthic animals may have a different exposure scenario than pelagic animals. Details about the exposure conditions and partitioning coefficients that could influence the test substance's bioavailability, bioaccessibility, or both should be provided, including for ionisable compounds tested across environmentally relevant pH gradients. Expert judgement should be used to determine the sampling strategy that best accounts for the life habits of the animal as it pertains to the bioavailability and/or bioaccessibility of the test substance, as well as the environmental compartments to be analysed.

16. Is the biomass loading of the organisms in the test system within the appropriate range (e.g. <1 g/l)?

CRED (corresponds with CRED reliability criterion #16): "Especially for hydrophobic substances, organism loading should be taken into account to avoid loss of the test substance by sorption to biota. This is mainly relevant for studies with larger organisms, like fish and macrophytes. Sorption to biota may become relevant when testing substances with log K_{OW} values >3. In addition, density stress may interfere with the effects of the chemical substance. The OECD guideline for acute fish toxicity tests recommends a maximum loading of 1.0 g fish/L for static and semistatic tests. For flow-through systems, higher biomass loadings may be acceptable if this does not cause a decrease in concentration of the test substance due to sorption to biomass.

Example 9: An experiment is performed using 30-L aquaria containing 40 fish of 1.5 g each (biomass loading of 2 g/L). If the experiment is static, the biomass loading would be too high. However, when a flow-through study is performed with a renewal rate of, for example, 90 L/d, the biomass loading would be acceptable." (Moermond *et al.*, 2016, p. 1303)

EthoCRED: Biomass loading (i.e. the size and density of groups of conspecifics) is important in behavioural testing as it may affect the behaviours of the test organisms and their response to chemical exposure (see EthoCRED reliability criteria #11 and #17), and loadings may also

impact the exposure concentration (due to potential uptake or sorption of the chemical). The CRED biomass loading criterion is relevant to both the exposure and the behavioural testing phases of behavioural ecotoxicity studies.

17. Is the behaviour-testing environment appropriate for the experimental organism and research question(s) (e.g. size and shape of trial arenas, time window for testing, avoidance of chemical, visual, and auditory interference)?
† Criterion specifically relates to behavioural ecotoxicity studies.

EthoCRED: To facilitate behavioural observation, animals are often transferred from an exposure system to one or more observational arenas. Many different assays and setups exist to score behaviour, which typically vary widely among studies and depend on the tested species and/or life stage. Because behaviour can be affected by many factors, and may reflect various underlying motivational and cognitive mechanisms, tests should ideally be validated for the life stage and species under investigation (Thoré *et al*., 2020). For instance, mirror-tests are often used to assess fish aggressiveness (Balzarini *et al*., 2014) but may instead reflect sociability (Cattelan *et al*., 2017) or even self-recognition (Kohda *et al*., 2022). When tests are not yet validated, they should at least be tailored to the biology of the test organism (e.g. open-field arenas should be large enough for animals to be active and display exploratory behaviour without experiencing confinement stress; see also EthoCRED relevance criterion #5), and care should be taken when interpreting the results. To facilitate expert judgement, studies should provide full methodological details on the experimental setup, similar to the factors mentioned for the exposure system (see EthoCRED reliability criterion #11). For instance, the shape and dimensions of the observational arena(s), including the characteristics of the potential environmental enrichment, and social context (e.g. presence or absence of conspecifics and/or heterospecifics, density, group composition), should be reported given that this may affect how animals behave and respond to chemicals (Kohler *et al*., 2018; Henry *et al*., 2022; Michelangeli *et al*., 2022).

Likewise, abiotic environmental parameters, including temperature, light/dark conditions, and water chemistry (for aquatic studies) should be reported and kept constant throughout the assay and across trials. Ideally, these conditions should be similar to those of the exposure system to avoid stress that is associated with changes in environmental conditions other than the treatment under investigation, unless such changes are functional to the behavioural assay (e.g. testing the behavioural response to a change in temperature). In cases

where wind tunnels or choice flumes are used to test attraction or avoidance in animals, it is critical that flows are not turbulent to prevent mixing of cues over the entire duration of the assay (Jutfelt *et al*., 2016). For tests on aquatic species, water quality measures (e.g. ammonia and nitrite levels) should be kept within appropriate ranges. Testing arenas for aquatic species should ideally also have their water partially or fully changed, and potentially be cleaned, between trials to avoid decreased bioavailability of the test substance and to maintain good quality of the medium (e.g. no build-up of animal waste or cross-contamination of chemical cues, maintenance of oxygen levels) (see EthoCRED reliability criteria #11 and #16). Importantly, water changes and/or cleaning should be consistent across all (exposed and unexposed) experimental treatments, to ensure comparability across treatments. Furthermore, arenas should be protected from potential unwanted disturbances (e.g. visual, auditory, vibrational), with the notable exception of multi-stressor studies that may deliberately manipulate such additional stressors. Because behaviour can fluctuate diurnally (Melvin, 2017; Thoré *et al*., 2019*a*), trials should be conducted within a restricted time window to limit potentially confounding behavioural variation, and the timing of observations should be standardised (or randomised) across experimental conditions (Thoré *et al*., 2021*a*) unless doing otherwise can be justified (e.g. during a timeframe when it is known that there is no diurnal change). When the behaviour-testing environment is not appropriate, this may be noticeable in aberrant behaviour of control animals and could render a study unreliable, although this may not always be noticeable and expert judgement is needed to make this evaluation.

18. If relevant, was an acclimation period employed before behavioural trials?

† Criterion specifically relates to behavioural ecotoxicity studies.

EthoCRED: The timeframe over which experimental animals are allowed to acclimate to behavioural arenas (i.e. the behaviour-testing environment) can influence the quality of behavioural data. At least two studies have explored the timeframe required for fish to reach a baseline behavioural status after being transferred into a new test environment, and these showed that optimising acclimation time led to improved baseline data for five different species (Melvin *et al*., 2017; Makaras *et al*., 2021). The consequence of insufficient acclimation is that response data (effect size) may reflect the combined impact of both the experimental treatment and general stress, leading to interpretations that cannot be extrapolated to natural environments with confidence. If exposure and data collection are performed in different systems (i.e. exposure system and behaviour-testing environment), experimenters should report the

acclimation time prior to the start of data acquisition. Species-specific acclimation data should ideally be collected whenever possible to demonstrate that animals are exhibiting baseline behaviours and not experiencing stress associated with transfer to a new environment.

There exists a large diversity of behavioural endpoints and experimental systems (e.g. various testing apparatuses), and while these recommendations may be crucial for many standard study designs, there are also scenarios where short acclimation durations are highly relevant (and perhaps even critical to the study goal). For example, short acclimation times are inherently necessary for tests of anxiety or exploratory behaviour in a novel environment, such as with the well-established novel tank diving test (Levin, Bencan & Cerutti, 2007). Similarly, it is also important to ensure that the duration of acclimation is not so long as to result in habituation effects (i.e. reduction in responsiveness), which can also lead to unreliable data (Blumstein, 2016; see EthoCRED reliability criterion #25). Hence, studies should ideally report the appropriateness of the acclimation period for the test organism (e.g. species, life stage) and the goal of the experiment.

19. Is the duration of behavioural trials reported?

† Criterion specifically relates to behavioural ecotoxicity studies.

EthoCRED: A wide range of study designs and test methodologies exists in behavioural ecotoxicology. Particularly the duration of behavioural trials (i.e. the length of time for data acquisition) is a factor that may vary widely and can influence the quality and validity of the measurements (Melvin *et al.*, 2017). First, it is important for all individual behavioural measures to be compared among trials of a study that are of equal duration (i.e. standardised trial duration) to avoid unwanted variation and/or to prevent systematic differences in behaviour due to methodological differences (also see EthoCRED reliability criteria #4 and #17). Second, the duration of the behavioural trials should be appropriate for the behaviour under study and tailored to the biology of the test organism. For instance, fish activity level is often measured as travelled distance during a particular timeframe, commonly through assays of 10–20 min (e.g. Ansai *et al.*, 2016; Thoré *et al.*, 2019*b*; Tan *et al.*, 2020). While behavioural measurement over a period of 10–20 min may yield a reasonably good approximation of the general activity level for many fish species, this may not be the case for all organisms (e.g. organisms that are more passive and/or show extended periods of inactivity) or behaviours (e.g. behaviours that may not be frequently expressed such as mating or displays of territoriality, or more complex behaviours such as nest building, may require longer study durations). Moreover, behavioural

data collected over a short duration (e.g. <10 min) can potentially be statistically less powerful (and thus more prone to error) than data collected over longer durations (Melvin *et al.*, 2017), which is particularly the case when short observation times lead to a low resolution of the data (e.g. zero-inflated data sets). Because different observational timeframes may influence the overall conclusions of behavioural analysis (Melvin, 2017; Melvin *et al.*, 2017), test durations should ideally be validated (i.e. robust and repeatable protocols) or justified in light of the species and life stage used, type, and overall context of the experiments that are conducted.

20. For feeding and foraging trials, were animals fed an appropriate amount and at an appropriate time relative to the commencement of behavioural trials? Is the kind and quantity of feed/prey used reported and appropriate?

† Criterion specifically relates to behavioural ecotoxicity studies.

EthoCRED: Feeding and foraging trials are commonplace in behavioural ecotoxicology. At their simplest, these trials measure the quantity of food consumed and/or the time taken to consume food (e.g. Bertram *et al.*, 2018*b*; Martin *et al.*, 2019*b*; Bose *et al.*, 2022*a*). The outcomes of such trials indicate both an individual's ability to feed and their motivation to feed. Thus, a critical consideration for feeding/foraging trials is standardising the hunger levels of animals before the trial. The experimenters should standardise the time since the animals were last fed, or were last given the opportunity to feed, prior to the beginning of the trial. Time since an individual last fed could alter its motivation to feed/forage (McNamara & Houston, 1986), and if not standardised, could introduce unwanted variability in the data. Thus, experimenters should report the food source, food quantity, and frequency of feeding in the lead up to the foraging/feeding trials. In standardising hunger levels, it is also important to consider what the motivational or energy homeostasis state of the animal should be during the time of the trial (Liu & Kanoski, 2018). For example, if animals are fed until satiation immediately prior to trials, there would be little to no motivation to feed during a trial. Therefore, there may be no observable differences across treatment groups simply because all animals were satiated. Therefore, it is ideal if the experiments withhold food for some amount of time before the feeding/foraging trial (the exact timing should be based on the biology of the model species). Another important consideration is the type and amount of food given to the animals during the trial. The amount and type of food provided to each animal should be consistent, or ideally normalised to the size of each individual (i.e. grams of food per gram body mass), and this information should always be reported.

21. In behavioural trials involving a predator, was an appropriate predatory stimulus used (e.g. was an anti-predator response observed in controls?)?
† Criterion specifically relates to behavioural ecotoxicity studies.

EthoCRED: Predator avoidance is a major aspect of prey decision-making and behaviour (e.g. where, when, and for how long to forage), and can influence a range of important life-history outcomes (e.g. reproduction, energy acquisition) (Lima, 1998). For this reason, studies in behavioural ecotoxicology are often interested in the effects of contaminant exposure on predator avoidance behaviours (e.g. freezing/inactivity, sheltering/hiding, threat/warning displays) as they are directly linked to survival probability. To elicit a typical fear or antipredator response in behavioural trials, researchers will often expose test subjects to a predatory stimulus. These stimuli can be visual, such as a predator model (e.g. Aimon *et al.*, 2022) or a live predator placed behind a transparent barrier (e.g. Mason *et al.*, 2021), tactile, through physical contact with a predatory stimulus (e.g. Orford *et al.*, 2023), auditory, by playing back real or simulated predator sounds (e.g. Tai *et al.*, 2023), or olfactory, such as predator chemical cues being added to the testing chamber (e.g. Saaristo *et al.*, 2019). These can also be combined, such as predator visual and olfactory stimuli, which typically elicits a more pronounced anti-predator reaction (e.g. Fursdon *et al.*, 2019; Cerveny *et al.*, 2020). A range of other anxiety-inducing stimuli are also commonly used in assays testing anti-predator or fear responses, including for example objects that are dropped into a testing chamber to elicit an escape response (e.g. Martin *et al.*, 2017). Importantly, predatory stimuli should be ecologically relevant and replicate a threat that the prey species has experienced, either through a recent encounter with the actual predator, or in its evolutionary past. If test subjects are naïve to the predator stimuli, this will likely not elicit a relevant or typical antipredator response (Sih *et al.*, 2010). Lastly, studies can also allow prey and predators to interact freely in behavioural trials, in which case predation may be directly observed (e.g. Lagesson *et al.*, 2018). This is relatively rare due to ethical constraints, and so is more often seen with, for example, invertebrate prey (e.g. Bose *et al.*, 2022*b*).

There are several key considerations when assessing the appropriateness of the predatory stimulus used in a behavioural trial. These include the ecological relevance of the chosen predator stimuli (e.g. does the prey species encounter the predator in its natural environment?), how well the predator stimulus replicates a natural predator cue (e.g. is the model predator a realistic size, shape, and colour?), and the signal capacity of the stimuli for

prey detection (e.g. can the prey species adequately detect and recognise the stimuli?). At a minimum, a description of the predator stimulus should be included in the methodology of the research paper, and ideally, the above considerations should also be adequately addressed.

22. Were behavioural trials recorded (e.g. video and/or audio recordings)?
† Criterion specifically relates to behavioural ecotoxicity studies.

EthoCRED: Experimental trials in research fields such as behavioural ecology have conventionally been scored live by one or more observers. Although manual scoring does not necessarily render a study unreliable, it may be prone to bias and have a comparatively low reproducibility (see Henry & Wlodkowic, 2020; Bownik & Wlodkowic, 2021*b*). Manual scoring can also present a heightened risk of external interference with animal behaviour during trials, for example due to visual or auditory disruptions produced by the observer(s). Researchers using manual scoring should therefore take appropriate measures to counter such pitfalls as much as possible, for instance by adopting blind scoring (see EthoCRED reliability criterion #23) and appropriate randomisation (see EthoCRED reliability criterion #24), as well as reducing any potential for disturbances (e.g. by scoring behaviour from behind a screen). As an alternative to manual scoring, digital data acquisition is increasingly adopted to obtain behavioural measures (Henry, Rodriguez & Wlodkowic, 2019; Simão *et al.*, 2019; Henry & Wlodkowic, 2020), reducing data collection errors and bias, and therefore increasing the reliability and reproducibility of results (Henry & Wlodkowic, 2020; Bownik & Wlodkowic, 2021*b*). Electronic data recording can be performed using diverse opto-electronic and digital video-recording systems (reviewed in Bownik & Wlodkowic, 2021*a*,*b*), as well as using remote-sensing systems such as acoustic telemetry (Bertram *et al.*, 2022; Hellström *et al.*, 2022) or sound-triggered recordings of vocalisations (Hoffmann & Kloas, 2010). Video recording using digital cameras is, in this context, applicable for most behavioural ecotoxicity experiments conducted in laboratory conditions. Moreover, coupling video recording of behavioural trials with subsequent analysis of trial videos using animal-tracking software provides not only digitally recorded data archives but also relatively unbiased analytical workflows (Henry & Wlodkowic, 2020).

From the perspective of behavioural ecotoxicity trial reporting, authors should ideally provide information on how the recording was conducted, although absence of such information does not necessarily render a study unreliable. For instance, authors should preferably report the make and model of camera and the settings used (e.g. resolution of the camera sensor, frame

rate of the video, sensitivity of the sensor), what illumination sources were used and their parameters (e.g. light intensity, spectral profile of the light source), and what kind and version of animal-tracking software was used (if any), including key settings (e.g. whether smoothing filters were applied, how missing tracks were dealt with).

23. Was/were the experimenter(s) blind to experimental treatment when conducting and analysing behavioural trials?

† Criterion specifically relates to behavioural ecotoxicity studies.

EthoCRED: Studies in behavioural ecotoxicology typically involve one or more researchers conducting experimental trials that comprise observing animals and scoring the frequency and/or duration of behaviours of interest. Trials may be observed live and/or video recorded, with behaviours being scored by hand or, more commonly, using behaviour-analysis software – often including programs that log key presses (e.g. BORIS; Friard & Gamba, 2016). In general, the experimenter(s) should be blinded to the experimental treatments (e.g. chemical exposure) when conducting and analysing behavioural trials in order to avoid potential bias (Holman *et al.*, 2015; Parker *et al.*, 2016). Blinding removes both conscious and unconscious biases, including confirmation bias, where an experimenter may preferentially detect and focus on outcomes that confirm prior beliefs (Nickerson, 1998). In practical terms, blinding can be accomplished by, for example, codifying experimental animals, exposure containers, trial arenas, and/or video recordings.

Non-blinding (or incomplete blinding), however, does not automatically make a study unreliable. For instance, although rare in behavioural ecotoxicity studies, blinding may not have been possible. This is the case when the experimental design involves treatments that are perceptible by the observer (e.g. visible chemical exposures such as wastewater effluent, male *versus* female in sexually dimorphic species, single animal *versus* group, small *versus* large animal), in which case the observer(s) should be blind to the hypothesis of the experiment. Alternatively, blind observation may have been used but not reported (discussed in Kardish *et al.*, 2015). This is difficult or impossible to disentangle from not having used blinding and should therefore be avoided by researchers. Furthermore, increasingly advanced behavioural analysis software options for quantifying animal behaviour (reviewed in Bertram *et al.*, 2022) may limit or remove the potential for experimenter effects during the behaviour-scoring process, therefore partially or fully removing the need for blinding at this step. For example, software designed to track animals individually or in groups is increasingly being used in

behavioural ecotoxicology research (e.g. ToxTrac; Rodriguez *et al.*, 2018). In the case that analysis of video files of recorded behavioural trials is fully automated, blinding of this experimental stage is not necessary. However, if the analysis is only partially automated, including if experimenters manually 'corrected' inaccurate software-generated animal tracks, any manual interaction with data collection and/or analysis should be blinded. Moreover, given the wide variety of video-tracking software options available, it is important that the name of the tracking software used is reported, as well as the version number where relevant.

24. If relevant, were experimental design elements appropriately randomised (e.g. assignment of animals to treatment groups, treatment type in behavioural trials, behavioural trial type in repeated testing, treatments across arenas in simultaneous testing, potential edge effects)?

† Criterion specifically relates to behavioural ecotoxicity studies.

EthoCRED: Animal behaviour, including behavioural responses to chemical exposure, is typically sensitive to a wide range of environmental and experiential factors (Bell, 2013). For instance, juvenile Nile tilapia (*Oreochromis niloticus*) reared at a high density were more neophobic and less aggressive compared to conspecifics that were reared at a low density, and these effects were more pronounced when tested in an arena without shelter compared to that with shelter (Champneys *et al.*, 2018). It is therefore necessary that, where at all possible, all procedures and conditions (other than the experimental treatment itself) are standardised for a study to be considered reliable (see also EthoCRED reliability criterion #4). To control further for the effect of extraneous/confounding variables and to avoid systematic errors (e.g. related to the order and/or timing of behavioural trials), experimental design elements should be appropriately randomised, as much as possible. For instance, the position of exposure and behavioural trial arenas should be randomised by treatment, wherever possible, to ensure that lighting, noise, and any other potential stimuli are as consistent as possible across the treatments – although potential extraneous stimuli should also be reduced as much as possible. Further, behavioural trials cannot be performed for all animals simultaneously and several rounds of observation (spread out over time) are necessary, meaning that trials should be performed in a random order with relation to the experimental condition of the animals (rather than first scoring all control animals, then scoring animals that were exposed to compound concentration 1, and so on). This randomisation over time is also important to control for potential diurnal changes in behaviour (Thoré *et al.*, 2021*a*; see also EthoCRED reliability criterion #17). In cases where

multiple behaviours are scored (i.e. when animals are successively subjected to different behavioural assays), researchers can either randomise the order of the assays or adopt a fixed order (the advantages and disadvantages of which are discussed in detail by Bell, 2013). However, systematic differences in the ordering of assays with respect to the experimental treatment render a study unreliable (unless sufficient justification is given, which may require expert judgement).

25. If animals were repeatedly tested using the same behavioural assay, were habituation effects accounted for?

† Criterion specifically relates to behavioural ecotoxicity studies.

EthoCRED: Because behaviour is a labile trait that naturally varies across time and context, researchers may adopt repeated-measures designs in which individuals are repeatedly tested using the same behavioural assay to increase statistical rigour and/or to account for intraspecific variation that extends beyond mean behavioural change (e.g. Polverino *et al.*, 2021; Thoré *et al.*, 2021*c*). However, animal responsiveness to repeated stimulation (such as during a series of behavioural assays) can progressively decrease as animals may acclimate/habituate to the setup and/or lose sensitivity to stimuli after continual exposure (Raderschall, Magrath & Hemmi, 2011; Bell & Peeke, 2012). For instance, the novel object test relies on avoidance/inspection behaviour exhibited towards an unfamiliar object in a familiar environment and is a standard paradigm to score neophobia/boldness in various animals (Frost *et al.*, 2007; Brunet *et al.*, 2022). When animals are repeatedly presented with the same stimulus (i.e. the same object), they may over time learn to recognise the object and adjust their response accordingly. This means that the scored behaviour may no longer reflect the same underlying motivational mechanisms as during the original trial, which could complicate interpretation of the results. Furthermore, individuals can vary in their habituation rates (Bell & Peeke, 2012), and habituation speed could conceivably vary with chemical exposure, either of which could further confound the results of the test. Habituation effects do not necessarily mean that a study is unreliable, but they should be accounted for statistically (e.g. testing how behaviour changes across trials with relation to the experimental treatment) and/or factored into the interpretation of the results.

Apart from habituation, acclimation, and/or sensory adaptation, repeated behavioural trials may also burden or fatigue animals, in particular when stimuli are used that are intended to startle, disturb, or otherwise stress animals (e.g. repeated trials with a predator stimulus

absent and then present), and cause them to behave differently compared to earlier trials or to animals that were not subjected to the same stimuli. For this reason, studies can only be considered reliable when animals were given enough time to recover between trials, unless rapid retesting is appropriately justified and instrumental to the research goal. What is appropriate timing between trials depends on several factors, including the species, life stage, and type of behavioural test, and expert judgement is needed to make this evaluation.

26. Is a sufficient number of replicates used? Is a sufficient number of organisms per replicate used for all controls and test concentrations?

CRED (corresponds with CRED reliability criterion #17): "In general, the guideline requirements for number of replicates should be used. When a nonguideline study is evaluated, expert judgment is needed to assess whether the study design is appropriate to obtain statistically reliable results. Statistically significant results do not automatically mean that the study is reliable, especially when there have been flaws in the study setup or in the performance of the study. For example, the use of pseudoreplicates (see example 10) lowers the reliability.

Example 10: An experiment is performed with 4 different concentrations and a control. For this experiment, 5 aquaria are used (1 per concentration). Within the aquaria, 3 compartments are made to separate groups of animals. These 3 groups of animals are considered to be pseudoreplicates (they have the same container, water, light, and temperature conditions and could experience the same stress). Thus, because the test is performed with pseudoreplicates only, the reliability is lowered." (Moermond *et al.*, 2016, p. 1303)

EthoCRED: Since individual organisms can differ substantially in their behavioural responses, behavioural data are often characterised by high levels of variability, meaning that large sample sizes may be required to ensure sufficient statistical power and to avoid generating spurious effects (Jennions & Møller, 2003; but see Melvin & Wilson, 2013). In statistics, power refers to the probability that a hypothesis test can detect the existence of a true effect. However, research has shown that the power of behavioural studies can be very low (Jennions & Møller, 2003). This means that if sample sizes are small, a non-significant result does not necessarily mean that there is no effect; it could simply reflect low statistical power due to insufficient replication. Issues of statistical power are especially pertinent when evaluating the reliability of non-significant results, as there is a risk of committing type-II error (i.e. erroneously concluding that there is no effect). Such a possibility can be influenced by the kinds of statistical test(s)

employed (Jennions & Møller, 2003), as well as whether and how statistical corrections are applied for multiple tests or comparisons (which can further exacerbate the likelihood of type-II errors; Nakagawa, 2004). Of course, study design is also important, and, from a replication standpoint, even a statistically significant result may be unreliable if the results are based on a study design where the wrong entity has been replicated (i.e. pseudoreplication; *sensu* Hurlbert, 1984). Therefore, when considering whether there is a sufficient number of replicates, it may be prudent also to consider what, exactly, the unit of replication in the experiment is (e.g. the number of individual animals *versus* the number of enclosures in which the animals are being housed or exposed; see Marshall, 2024). The latter may be especially pertinent in the case of social species, where the experimental unit of replication will be at the group (rather than individual) level if animals are being tested collectively as a group. In this regard, the level of replication on which statistical analysis is based (e.g. the number of individuals or groups) is determined by the study design and the type of statistical test being performed. Expert judgment is sometimes required to determine if the replication level is appropriate.

27. Are appropriate statistical methods used?

CRED (corresponds with CRED reliability criterion #18): "In general, the guideline requirements for statistics should be followed and a description of the statistics is needed to assess the reliability of an endpoint. When a nonguideline study is evaluated, expert judgment may be needed.

If effect values are missing, concentration–response data reported in tables or graphs can be used to calculate them. For example, the concentration at which 10% effect is observed (EC_{10}) can usually be calculated when a concentration–response curve is available, and computer programs are available to translate graphs into individual data points (e.g. Techdig). However, effect values should be determined by interpolation and not by extrapolation, and they should preferably not exceed or be lower than the tested concentrations. A calculated EC_{10} value that is more than 3 times lower than the lowest tested concentration is less reliable. An NOEC value should be determined using an appropriate statistical method and should not be determined by visual inspection of the graphs or other estimation without statistical significance being determined." (Moermond *et al.*, 2016, p. 1303)

EthoCRED: The use of appropriate and accepted statistical tests is critical for the robust evaluation of behavioural toxicity data. In any study, the choice of statistical methods should reflect the nature of the data (e.g. categorical, binomial, count), any underlying assumptions of the statistical test have to be met, and any potential biases or interpretive errors should not be introduced through the analysis. At the broadest level, statistical analyses typically involve a choice between parametric and non-parametric methods. Parametric methods are commonly applied to hypothesis testing [e.g. *t*-test, analysis of variance (ANOVA)] but rely on underlying assumptions about the characteristics of the data (e.g. error distribution, homogeneity of variance, minimum number of replicates) which must be met for the test to be valid. Should data fail to meet these assumptions, an equivalent and appropriate non-parametric method should be used. The experimenter must demonstrate that the data have been carefully considered such that the choice of analysis is suitable, and any transformation or normalisation steps applied to the data should be reported in full.

For studies that aim to make dose–response claims (see also EthoCRED reliability criteria #13 and #28), the study design and subsequent statistical analysis should allow for the determination of a reliable concentration that produces a given level of effect (EC, effective concentration), or alternatively, a concentration below which the effect is not distinguishable from background noise. Such values should be derived from interpolation rather than extrapolation, which implies that the EC falls between the lowest and highest concentrations tested in the study. Concentration–response modelling methods (e.g. regression) are generally preferable over hypothesis-testing methods (e.g. *t*-test, ANOVA) for determining a reliable EC, but these have their own sets of assumptions, including that the response follows a monotonic concentration–response pattern [i.e. there is no change in the sign (positive/negative) of the slope over the range of concentrations tested]. It is also common for studies to test just a few concentrations because of logistical (hence power) constraints, in which case ANOVA or mixed modelling approaches may be used, with concentration as a fixed factor rather than as a continuously distributed variable. When using such an approach, *post-hoc* tests among concentrations can be very informative.

Data in behavioural ecotoxicology – and ecotoxicology more generally – are typically hierarchically structured. This means that multiple exposure containers are usually assigned to each treatment group, and multiple individuals are usually assigned to each of those exposure containers, and (where applicable) multiple measures may be taken for each individual. Statistical analyses should account for this hierarchical structuring in order to make accurate inferences about the effects of contaminants on endpoints of interest. For instance, in cases

where the behaviour of individual organisms is tested repeatedly, care should be taken to make sure that this is accounted for in statistical analysis – e.g. through the use of mixed-effects models, which can also be called ‘hierarchical’ models or ‘random-effects’ models (reviewed in Arnqvist, 2020). This approach is necessary if data are collected repeatedly from the same individuals and used in the statistical analyses because these data are non-independent. However, mixed-effects models may not always be necessary in cases where data-reduction approaches have been used (e.g. if all observations of each animal have been averaged).

Numerous resources provide discussion of statistical principles and commonly used techniques in ecotoxicology (OECD, 2006; Green, Springer & Holbech, 2018). When missing values or problematic data are encountered, consultation with an experienced and qualified statistician is recommended to ensure robust and reliable interpretation of behavioural data.

28. Is a concentration–response relationship observed?

CRED (corresponds with CRED reliability criterion #19): “The requirement for a concentration–response relationship depends on the objective of the study. If an effect needs to be demonstrated, a concentration–response relationship is needed. However, when the study has been performed to verify that there is no effect at a certain dose, a concentration–response relationship is not necessarily needed to derive a NOEC value. Generally, if no monotonic concentration–response curve is observed, it is difficult to obtain reliable endpoints. Exceptions occur, for instance when increased growth is observed at low concentrations and a toxic effect is observed at high concentrations. The concentration–response curve can then be difficult to calculate, and a calculation model that allows for these effects is needed. When limit tests (with just one or two concentrations) are performed, no concentration–response curve can be observed. However, a properly designed limit test based on range-finding data and conducted at the limit of solubility is reliable, as long as no adverse effects are observed. If adverse effects do occur, then the study alone cannot be used to calculate a safe concentration.

If tables or graphs (which can be transformed back into numbers) with concentration–response data are available, effect values may be calculated. However, calculating a statistically significant NOEC value will not be possible if raw data or standard errors are missing. Nevertheless, it may then be possible to calculate an EC_{10} value, with statistical significance values, using programs such as GraphPad Prism. If endpoints with their statistical method are provided but no concentration–response graph or table is reported, the study can still be assigned to be “reliable with restrictions” (R2).” (Moermond *et al.*, 2016, p. 1303)

EthoCRED: The concentration–response (dose–response) relationship is a key principle in toxicology (see also EthoCRED reliability criterion #13), and a critical component of regulatory toxicology since it helps to demonstrate causality between chemical exposure and a biological effect. A quality concentration–response curve should comprise a broad range of test concentrations, including a dose below which there is no effect (NOEC). With no concentration–response curve, or a very limited curve (few concentrations tested and not spanning a NOEC), it is not possible to establish a meaningful EC from behavioural toxicity data. Difficulties also arise when the concentration–response relationship is non-monotonic; in other words, when the slope of the curve changes sign (positive/negative) along the concentration gradient tested – as is, for example, often the case when studying endocrine-disrupting chemicals. In such instances, there may be uncertainty regarding the potential for effects at very low concentrations and it is not possible to establish a reliable EC. Contrarily, non-monotonicity at high concentrations, but a linear relationship at lower concentrations, should not influence the ability to characterise risk and, thus, to establish an EC.

Behavioural toxicology studies are applied very broadly and are not always intended to establish absolute dose–response relationships. In many cases, behavioural endpoints may be explored when such relationships exist for other traditional endpoints. In these scenarios, a researcher may be interested in characterising potential behavioural effects towards the lower end of an existing dose–response curve and/or at an environmentally relevant dose, and therefore a wide concentration gradient is not required for behavioural data to offer additional evidence of risk. Similarly, if the goal of a study is to verify a lack of response at a specific concentration (e.g. with increased replication), a concentration–response relationship may not be necessary for a study to provide meaningful information. This may be particularly relevant for behavioural data since it can be logistically difficult to achieve adequate replication for robust analysis while also including a wide range of concentrations, and since behavioural responses are often more sensitive than other endpoints (e.g. developmental or reproductive traits; Melvin & Wilson, 2013). Thus, behavioural data without concentration–response relationships can provide meaningful evaluation of an EC calculated from non-behavioural data, but the identification of a response at or below an existing NOEC would signify the need for further behavioural testing with an acceptable concentration gradient to establish a valid behavioural EC. As mentioned above (see EthoCRED reliability criterion #13), it is also important not to discount sub-threshold effects in chemical risk assessments just because a non-standard dose–response is observed (Agathokleous *et al.*, 2022).

29. Are sufficient data available to check the calculation of endpoints and (if applicable) validity criteria (e.g. control data, raw data, dose–response curves)?

CRED (corresponds with CRED reliability criterion #20): "The availability of raw data is not a prerequisite for a study to be reliable. However, where "reliable with restrictions" (R2) can be assigned if raw data are not reported, "reliable without restrictions" (R1) can only be assigned when raw data are provided. By "raw data" we mean the data needed to assess the statistics and variability in the controls, recalculate the reported endpoints, and calculate alternative endpoints. These data may be presented in the form of tables or graphs, in the publication itself, or, in case of peer-reviewed papers, in the supplemental data." (Moermond *et al.*, 2016, p. 1303)

EthoCRED: Journals and funding agencies are increasingly mandating that authors make the raw data and statistical code used to obtain their results publicly available (e.g. in archived data repositories; Bertram *et al.*, 2023). Data sharing allows for greater transparency and replication of experiments, which can increase trust in published findings and promote collaboration and further advances (Bertram *et al.*, 2023). It is important to realise, however, that the absence of raw data does not, in itself, mean that a study is unreliable, especially when considering that, historically, the availability of such data was not a prerequisite for study publication and, in some fields, may still be a relatively uncommon practice. Rather, the availability of raw data can contribute to an assessment of a study's reliability by allowing readers to understand, evaluate, and reproduce a study's findings and conclusions (Gomes *et al.*, 2022). Beyond transparency in sharing raw data for all response variables measured in a test, it is also valuable to include data relevant to quality assurance and control (e.g. analytical verification of exposure concentrations, see EthoCRED reliability criterion #15; use of appropriate positive and/or negative controls, see EthoCRED reliability criterion #4).

It is important also to emphasise that the mere availability of raw data is not a guarantee of a study's reliability. Here, it is critical to consider the quality of the provided data. In this regard, the current onus on data archiving, along with creating clear and complete archives, typically rests with authors. As a result, the quality of raw data that are shared can be highly variable and may not always be sufficient to enable the study's reliability to be properly evaluated (e.g. authors sharing incomplete and/or indecipherable data sets, or providing summary statistics instead of actual raw data).

Consequently, in line with the original CRED reliability criterion #20 (Moermond *et al.*, 2016), the availability of raw data is not a prerequisite for a study to be reliable, although it is certainly beneficial for researchers to make their data publicly available for the aforementioned reasons.

Step 4: Assign reliability category

Based on the overall conclusion from the reliability evaluation, the appropriate reliability category (as described in Table S4) is assigned to the study.

Table S4. EthoCRED reliability categories. Note that these categories correspond with the original Criteria for Reporting and Evaluating Ecotoxicity Data (CRED) reliability categories outlined by Moermond *et al.* (2016), which were adapted from Klimisch *et al*. (1997).

**Reliability category**	**Description**
Reliable without restrictions	All critical reliability criteria for this study are fulfilled. The study is well designed and performed, and it does not contain flaws that affect the reliability of the study.
Reliable with restrictions	The study is generally well designed and performed, but some minor flaws in the documentation or setup may be present.
Not reliable	Not all critical reliability criteria for this study are fulfilled. The study has clear flaws in study design and/or how it was performed.
Not assignable	Information needed to make an assessment of the study is missing. This concerns studies that do not give sufficient experimental details and that are only listed in abstracts or secondary literature (books, reviews, etc.) or studies for which the documentation is not sufficient for assessment of reliability for one or more vital parameters.

Step 5: Combine relevance and reliability

Upon evaluating the relevance and reliability of a study, its overall adequacy for assessment or regulatory purposes can be assessed according to the approach summarised in Fig. S2.

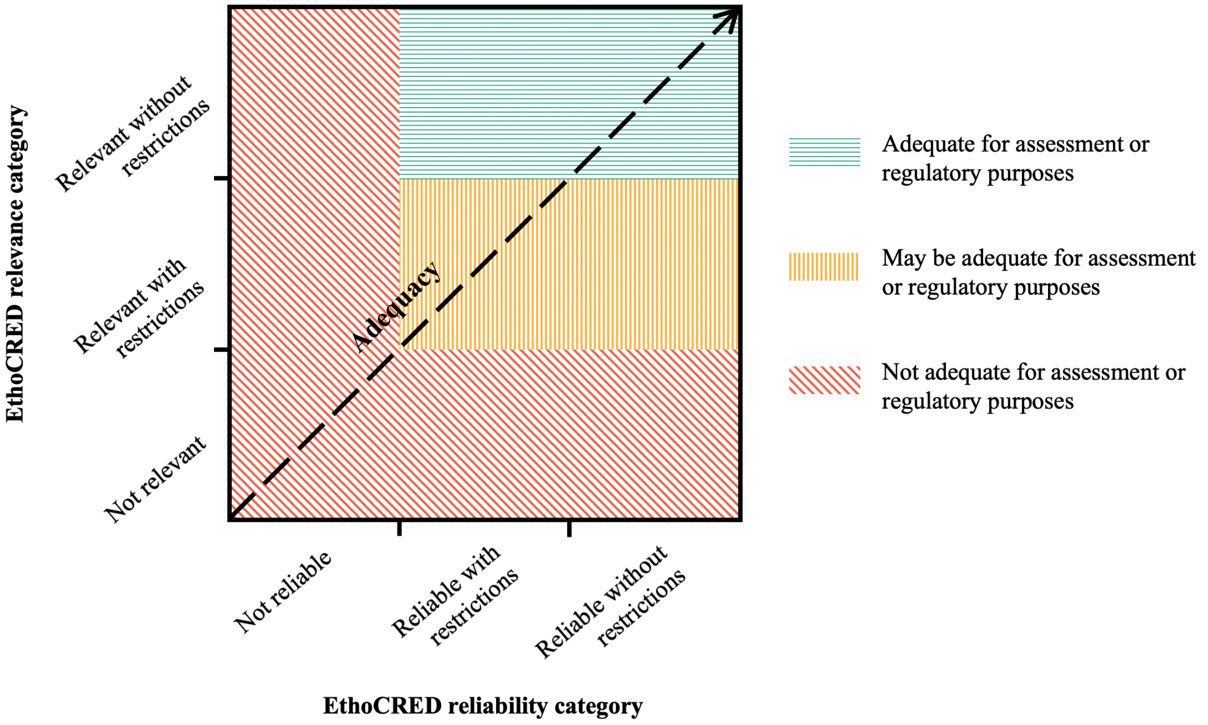

Fig. S2. Diagram illustrating an approach for summarising the adequacy of behavioural ecotoxicity data for assessment or regulatory purposes using the EthoCRED relevance and reliability categories. Adapted from Ågerstrand *et al.* (2011), Moermond *et al.* (2016), and Hartmann *et al.* (2017).

Using this approach, a study in behavioural ecotoxicology is considered 'adequate for assessment or regulatory purposes' if it is reliable without restrictions *or* reliable with restrictions *and* relevant without restrictions. Studies considered adequate for assessment or regulatory purposes would, for example, be used as key studies to derive PNEC values for risk assessment purposes. Further, a study 'may be adequate for assessment or regulatory purposes' if it is reliable without restrictions *or* reliable with restrictions *and* relevant with restrictions. Studies that may be adequate for assessment or regulatory purposes could, for example, be used as supporting evidence in a risk assessment, or could be used for PNEC derivations in cases where limited data are available. Behavioural ecotoxicology studies that are considered not relevant *and/or* not reliable are considered 'not adequate for assessment or regulatory purposes'.

EthoCRED reporting recommendations

The EthoCRED reporting recommendations have been formulated as an extension of the original CRED reporting recommendations (Moermond *et al.*, 2016) and encompass 72 criteria distributed across seven categories: general information, test design, test compound, test organism, exposure conditions, assessing biological responses, and statistical design and analysis (Table S5). These reporting recommendations have been designed to align with the reliability criteria from the EthoCRED evaluation method, meaning that the guidance material associated with the reliability evaluation method is also a useful resource for researchers in behavioural ecotoxicology when designing their studies. In this regard, researchers undertaking behavioural ecotoxicity studies are advised to familiarise themselves with the EthoCRED reporting recommendations during the early phases of experiment design to ensure that all factors contributing to reliability are considered.

Table S5. The EthoCRED reporting recommendations, containing 72 specific aspects to consider when reporting behavioural ecotoxicity studies. Importantly, not all recommendations are relevant for every study, but it is good practice to address the relevant criteria in a clear and transparent fashion. In cases where certain relevant reporting recommendations cannot be addressed, EthoCRED recommends that authors transparently explain the reason(s) behind the omission of this information.

**EthoCRED reporting recommendations**	
**1**	**General information**
	a. Purpose of study
	b. Description of endpoints
	c. Biological/ecological basis for the behavioural endpoint(s) investigated, with supporting evidence
	d. Population-level relevance of the behavioural endpoint(s) investigated, with supporting evidence
**2**	**Test design**
	a. Performed according to standard/modified standard (e.g. OECD, USEPA)
	b. Performed according to good laboratory practice (GLP)
	c. Description of control(s): negative control, solvent control, positive control
	d. Control(s) mortality, growth, morbidity, and other observed non-standard effects such as changes to colouration
	e. Comparison to validity criteria (e.g. control survival, growth) from appropriate guideline test method

3 Test compound

a. Identification (e.g. name, CAS number, specify if the salt or the base is tested)

b. Physicochemical characteristics that may influence the behaviour of the compound during the study [e.g. solubility, volatility, stability (hydrolysis, photolysis, degradation), solubility, log K_{OW}, degradability, adsorption]

c. Source (e.g. manufacturer, product code)

d. Purity percentage

e. Composition of product formulation and presence of impurities

4 Test organism

a. Scientific name

b. Relevant morphological characteristics (e.g. body mass, length)

c. Age/life stage

d. Growth/reproductive condition

e. Sex

f. Strain, clone

g. Source (e.g. wild-collected, laboratory stock, commercial supplier), ideally including analytical chemistry verification ruling out or characterising potential pre-exposure of organisms to the test compound/other contaminants

h. Acclimation to laboratory conditions (e.g. duration, feeding, housing conditions)

5 Exposure conditions

a. Exposure route (e.g. waterborne, soilborne, airborne, dietary and/or injection)

b. Exposure schedule (static, semistatic, flow-through system, other) and flow rate (flow-through systems) or renewal time (semistatic systems)

c. Open or closed system

d. Test medium composition (e.g. source of test water: well water, deionised water, tap water)

e. Temperature and time points for measuring

f. pH and time points for measuring

g. Hardness of water and time points for measuring

h. Conductivity/salinity and time points for measuring

i. Dissolved oxygen content and time points for measuring

j. Light intensity and quality (e.g. source, light spectrum, homogeneity), light/dark conditions

k. Feeding protocols, food composition

l. Material and volume of aquarium/container and other equipment in contact with test organisms and test substance

m. Use of sand or sediment and its characteristics (e.g. total organic carbon, particle size)

n. Preparation of stock solutions, including solvent concentrations in test water and controls for aquatic studies

o. Nominal concentrations of test substance

p. Measured concentrations of test substance and time points for measuring, including exposure media (e.g. water, soil, air) and organism tissues (e.g. brain, muscle, liver)

q. Analytical method: description of method, including limit of detection and limit of quantification

r. Exposure duration and total test duration

s. Time points of observations for endpoints (behavioural and non-behavioural)

t. Results based on nominal or measured concentrations

u. Biomass loading (biomass per litre)

v. Exposed individually or in a group within each exposure container, including number of individuals per exposure container

w. Composition of groups when exposed with multiple individuals per container (e.g. single *versus* multiple species and/or sexes)

6 Assessing biological responses

a. Size and shape of behaviour trial arenas

b. Time window of behavioural testing (e.g. daily start and stop times of observation)

c. Measures taken to avoid chemical, visual, and/or auditory interference

d. Use and duration of acclimation period to behavioural trial arenas before the commencement of trials

e. Duration of behavioural trials

f. Description of feeding regime before – and, if relevant, during – behavioural trials, particularly important for feeding and foraging behaviour trials

g. Whether organisms continued to be exposed, or not, to the treatment(s) throughout some or all of the behavioural assay(s)

h. Behavioural trials carried out on individuals or groups, including number of individuals per trial

i. For behavioural trials on groups, composition of groups (e.g. single *versus* multiple species and/or sexes)

j. For trials involving a predator stimulus, the species, size, and type of stimulus used (e.g. live predator, model predator, predator animation)

k. For trials involving a predator, use of visual, chemical, physical, or auditory predator cues, or a combination

l. For trials involving a predator, ecological relevance of the predator species. Are they sympatric with the prey model species in the wild? Was an anti-predator response seen in controls?

m. Details of recording of behavioural trials (e.g. video and/or auditory recording) and/or live observation

n. Details of analysis of behavioural trials [e.g. software name and version, software parameterisation, manual or human scoring from videos (e.g. using JWatcher or BORIS) or supervised automated approaches (e.g. EthoVision XT, ZebraBox, ToxTrac)], and/or live observation

o. Biological response for each concentration and assay

p. Blinding of experimenter(s) conducting and analysing behavioural trials (e.g. partial or full blinding, non-blinded)

q. Randomisation of experimental design elements, if relevant (e.g. assignment of animals to treatment groups, treatment type in behavioural trials, behavioural trial type in repeated testing, treatments across arenas in simultaneous testing, potential edge effects)

r. Accounting for potential habituation effects (if animals were repeatedly tested using the same behavioural assay)

7 Statistical design and analysis

a. Number of replicates for control(s) and test concentrations; setup of replicates (avoid pseudoreplication)

b. Number of organisms per replicate

c. Treatment design (e.g. block, randomised)

d. Statistical methods used

e. Dose–response observed

f. Statistically significant responses noted (e.g. EC*x*)

g. Significance level for NOEC and LOEC data ($\alpha = 0.05$, or less)

h. Estimation of variability for LC*x* and EC*x* data

i. Availability of raw data: through supporting information, a website, or upon request (statements of data availability upon request should be avoided wherever possible)

CAS = Chemical Abstracts Service; CRED = Criteria for Reporting and Evaluating Ecotoxicity Data; EC*x* = *x*% effective concentration; K_{OW} = octanol–water partition coefficient; LC*x* = *x*% lethal concentration; LOEC = Lowest Observable Effect Concentration; NOEC = No Observable Effect Concentration; OECD = Organisation for Economic Co-operation and Development; USEPA = US Environmental Protection Agency.

Certain EthoCRED reporting recommendations are crucial for the reliability of a given study, while others carry relatively less weight. The significance of certain reporting recommendations often depends on experimental design variables such as the test organism, test duration, or test substance. For instance, specifying the sex and life stage of organisms tested for the effects of exposure to endocrine-disrupting chemicals is of high significance given that various endocrine disruptors are known to have effects that are dependent on these parameters, while reporting the parameters of behavioural software would be irrelevant in a study that used a manual,

software-free behaviour-scoring approach. Authors reporting behavioural ecotoxicity studies are strongly encouraged to incorporate a comprehensive and well-structured description of their experiments, supplemented with additional data if necessary. In cases where certain relevant reporting recommendations cannot be addressed, it is recommended that authors transparently explain the reason(s) behind the omission of this information. By doing so, evaluators of the study – such as peer reviewers, editors, fellow researchers, and risk assessors – can more easily assess the experimental design, outcomes, and potential limitations of the study. Adherence to the EthoCRED reporting recommendations is expected to reduce the potential for underreporting and information gaps within a published study. Furthermore, it is likely that a study following the EthoCRED reporting recommendations will undergo the peer-review process more efficiently.

Disclaimer

The views expressed in this paper are those of the authors and do not necessarily reflect the view or opinions of their institutions. The mention of trade names or commercial products does not constitute endorsement or recommendation for use. Any use of trade, firm, or product names is for descriptive purposes only and does not imply endorsement by the U.S. Government. This product (article, paper, etc.) has been peer reviewed and approved for publication consistent with USGS Fundamental Science Practices (https://pubs.usgs.gov/circ/1367/). The content is solely the responsibility of the authors and does not necessarily represent the official views of the National Institutes of Health.

References

AGATHOKLEOUS, E., BARCELÓ, D., ASCHNER, M., AZEVEDO, R. A., BHATTACHARYA, P., COSTANTINI, D., CUTLER, G. C., DE MARCO, A., DOCEA, A. O., DÓREA, J. G., DUKE, S. O., EFFERTH, T., FATTA-KASSINOS, D., FOTOPOULOS, V., GINEBREDA, A., *ET AL*. (2022). Rethinking subthreshold effects in regulatory chemical risk assessments. *Environmental Science & Technology* **56**, 11095–11099.

ÅGERSTRAND, M., ARNOLD, K., BALSHINE, S., BRODIN, T., BROOKS, B. W., MAACK, G., MCCALLUM, E. S., PYLE, G., SAARISTO, M. & FORD, A. T. (2020). Emerging investigator series: use of behavioural endpoints in the regulation of chemicals. *Environmental Science: Processes & Impacts* **22**, 49–65.

ÅGERSTRAND, M., BREITHOLTZ, M. & RUDÉN, C. (2011). Comparison of four different methods for reliability evaluation of ecotoxicity data: a case study of non-standard test data used in environmental risk assessments of pharmaceutical substances. *Environmental Sciences Europe* **23**, 1–15.

AIMON, C., LEBIGRE, C., LE FLOCH, S. & CLAIREAUX, G. (2022). Effects of dispersant-treated oil upon behavioural and metabolic parameters of the anti-predator response in juvenile European sea bass (*Dicentrarchus labrax*). *Science of The Total Environment* **834**, 155430.

ALMEIDA, R., LEMMENS, P., DE MEESTER, L. & BRANS, K. I. (2021). Differential local genetic adaptation to pesticide use in organic and conventional agriculture in an aquatic non-target species. *Proceedings of the Royal Society B: Biological Sciences* **288**, 20211903.

ANDREWS, P. W., BHARWANI, A., LEE, K. R., FOX, M. & ANDERSON THOMSON JR, J. (2015). Is serotonin an upper or a downer? The evolution of the serotonergic system and its role in depression and the antidepressant response. *Neuroscience and Biobehavioral Reviews* **51**, 164–188.

ANSAI, S., HOSOKAWA, H., MAEGAWA, S. & KINOSHITA, M. (2016). Chronic fluoxetine treatment induces anxiolytic responses and altered social behaviors in medaka, *Oryzias latipes*. *Behavioural Brain Research* **303**, 126–136.

ARNQVIST, G. (2020). Mixed models offer no freedom from degrees of freedom. *Trends in Ecology & Evolution* **35**, 329–335.

AULSEBROOK, L. C., BERTRAM, M. G., MARTIN, J. M., AULSEBROOK, A. E., BRODIN, T., EVANS, J. P., HALL, M. D., O'BRYAN, M. K., PASK, A. J., TYLER, C. R. & WONG, B. B. M. (2020). Reproduction in a polluted world: implications for wildlife. *Reproduction* **160**, R13–R23.

AULSEBROOK, L. C., WONG, B. B. M. & HALL, M. D. (2022). Warmer temperatures limit the effects of antidepressant pollution on life-history traits. *Proceedings of the Royal Society B: Biological Sciences* **289**, 20212701.

BALZARINI, V., TABORSKY, M., WANNER, S., KOCH, F. & FROMMEN, J. G. (2014). Mirror, mirror on the wall: the predictive value of mirror tests for measuring aggression in fish. *Behavioral Ecology and Sociobiology* **68**, 871–878.

BELL, A. M. (2013). Randomized or fixed order for studies of behavioral syndromes? *Behavioral Ecology* **24**, 16–20.

BELL, A. M. & PEEKE, H. V. S. (2012). Individual variation in habituation: behaviour over time toward different stimuli in threespine sticklebacks (*Gasterosteus aculeatus*). *Behaviour* **149**, 1339–1365.

BERTRAM, M. G., ÅGERSTRAND, M., THORÉ, E. S. J., ALLEN, J., BALSHINE, S., BRAND, J. A., BROOKS, B. W., DANG, Z., DUQUESNE, S., FORD, A. T., HOFFMANN, F., HOLLERT, H., JACOB, S., KLOAS, W., KLÜVER, N., *ET AL*. (2024). EthoCRED: a framework to guide reporting and evaluation of the relevance and reliability of behavioural ecotoxicity studies. *Biological Reviews*.

BERTRAM, M. G., ECKER, T. E., WONG, B. B., O'BRYAN, M. K., BAUMGARTNER, J. B., MARTIN, J. M. & SAARISTO, M. (2018*a*). The antidepressant fluoxetine alters mechanisms of pre- and post-copulatory sexual selection in the eastern mosquitofish (*Gambusia holbrooki*). *Environmental Pollution* **238**, 238–247.

BERTRAM, M. G., MARTIN, J. M., MCCALLUM, E. S., ALTON, L. A., BRAND, J. A., BROOKS, B. W., CERVENY, D., FICK, J., FORD, A. T., HELLSTRÖM, G., MICHELANGELI, M., NAKAGAWA, S., POLVERINO, G., SAARISTO, M., SIH, A., *ET AL*. (2022). Frontiers in quantifying wildlife behavioural responses to chemical pollution. *Biological Reviews* **97**, 1346–1364.

BERTRAM, M. G., SAARISTO, M., BAUMGARTNER, J. B., JOHNSTONE, C. P., ALLINSON, M., ALLINSON, G. & WONG, B. B. M. (2015). Sex in troubled waters: widespread agricultural contaminant disrupts reproductive behaviour in fish. *Hormones and Behavior* **70**, 85–91.

BERTRAM, M. G., SAARISTO, M., MARTIN, J. M., ECKER, T. E., MICHELANGELI, M., JOHNSTONE, C. P. & WONG, B. B. M. (2018*b*). Field-realistic exposure to the androgenic endocrine disruptor 17β-trenbolone alters ecologically important behaviours in female fish across multiple contexts. *Environmental Pollution* **243**, 900–911.

BERTRAM, M. G., SUNDIN, J., ROCHE, D. G., SÁNCHEZ-TÓJAR, A., THORÉ, E. S. J. & BRODIN, T. (2023). Open science. *Current Biology* **33**, R792–R797.

BLANCK, H., WÄNGBERG, S-Å. & MOLANDER, S. (1988). Pollution-induced community tolerance (PICT)—A new ecotoxicological tool. In Cairns, J. Jr. & Pratt, J.R. (Eds.), Functional Testing of Aquatic Biota for Estimating Hazards of Chemicals ASTM STP 988 (pp. 219–230). Philadelphia, PA: American Society for Testing and Materials. Available at: http://publications.lib.chalmers.se/publication/139772-pollution-induced-community-tolerance-pict-a-new-ecotoxicologicaltool-in-cairns-j-jr-pratt-jr-eds-f

BLUMSTEIN, D. (2016). Habituation and sensitization: new thoughts about old ideas. *Animal Behaviour* **120,** 255–262.

BOSE, A. P. H., BRODIN, T., CERVENY, D. & MCCALLUM, E. S. (2022*a*). Uptake, depuration, and behavioural effects of oxazepam on activity and foraging in a tropical snail (*Melanoides tuberculata*). *Environmental Advances* **8**, 100187.

BOSE, A. P. H., MCCALLUM, E. S., AVRAMOVIĆ, M., BERTRAM, M. G., BLOM, E. -L., CERVENY, D., GRØNLUND, S. N., LEANDER, J., LUNDBERG, P., MARTIN, J. M., MICHELANGELI, M., PERSSON, L. & BRODIN, T. (2022*b*). Pharmaceutical pollution disrupts the behaviour and predator–prey interactions of two widespread aquatic insects. *iScience* **25**, 105672.

BOWNIK, A. & WLODKOWIC, D. (2021*a*). Advances in real-time monitoring of water quality using automated analysis of animal behaviour. *Science of the Total Environment* **789**, 147796.

BOWNIK, A. & WLODKOWIC, D. (2021*b*). Applications of advanced neuro-behavioral analysis strategies in aquatic ecotoxicology. *Science of the Total Environment* **772**, 145577.

BRANS, K. I., ALMEIDA, R. A. & FAJGENBLAT, M. (2021). Genetic differentiation in pesticide resistance between urban and rural populations of a nontarget freshwater keystone interactor, *Daphnia magna*. *Evolutionary Applications* **14**, 2541–2552.

BRINGOLF, R. B., HELTSLEY, R. M., NEWTON, T. J., EADS, C. B., FRALEY S. J., SHEA, D. & COPE, W. G. (2010). Environmental occurrence and reproductive effects of the pharmaceutical fluoxetine in native freshwater mussels. *Environmental Toxicology and Chemistry* **29**, 1311–1318.

BRODIN, T., FICK, J., JONSSON, M. & KLAMINDER, J. (2013). Dilute concentrations of a psychiatric drug alter behavior of fish from natural populations. *Science* **339**, 814–815.

BRODIN, T., PIOVANO, S., FICK, J., KLAMINDER, J., HEYNEN, M. & JONSSON, M. (2014). Ecological effects of pharmaceuticals in aquatic systems—impacts through behavioural alterations. *Philosophical Transactions of the Royal Society B: Biological Sciences* **369**, 20130580.

BRUNET, V., KLEIBER, A., PATINOTE, A., SUDAN, P., DURET, C., GOURMELEN, G., MOREAU, E., FOURNEL, C., PINEAU, L., CALVEZ, S., MILLA, S. & COLSON, V. (2022). Positive welfare effects of physical enrichments from the nature-, functions- and feeling-based approaches in farmed rainbow trout (*Onchorhynchus mykiss*). *Aquaculture* **550**, 737825.

BUDAEV, S. & BROWN, C. (2011). Personality traits and behaviour. In Brown, C. Laland, K. N. & Krause, J. (Eds.), *Fish cognition and behaviour* (pp. 135–165). Oxford, UK: Wiley-Blackwell.

CANDOLIN, U. & WONG, B. B. M. (2019). Mate choice in a polluted world: consequences for individuals, populations and communities. *Philosophical Transactions of the Royal Society of London B* **374**, 20180055.

CATTELAN, S., LUCON-XICCATO, T., PILASTRO, A. & GRIGGIO, M. (2017). Is the mirror test a valid measure of fish sociability? *Animal Behaviour* **127**, 109–116.

CERVENY, D., BRODIN, T., CISAR, P., MCCALLUM, E. S. & FICK, J. (2020). Bioconcentration and behavioral effects of four benzodiazepines and their environmentally relevant mixture in wild fish. *Science of the Total Environment* **702**, 134780.

CHAMPNEYS, T., CASTALDO, G., CONSUEGRA, S. & DE LEANIZ, G. (2018). Density-dependent changes in neophobia and stress-coping styles in the world's oldest farmed fish. *Royal Society Open Science* **5**, 181473.

EHLMAN, S. M., TRIMMER, P. C. & SIH, A. (2019). Prey responses to exotic predators: effects of old risks and new cues. *The American Naturalist* **193**, 575–587.

ERICKSON, R. A. & RATTNER, B. A. (2020). Moving beyond $p < 0.05$ in ecotoxicology: a guide for practitioners. *Environmental Toxicology and Chemistry* **39**, 1657–1669.

EUROPEAN CHEMICALS AGENCY (2008). REACH guidance documents. Helsinki, Finland.

EUROPEAN CHEMICALS AGENCY (2011*a*). Guidance on information requirements and chemical safety assessment Chapter R.4: evaluation of available information. Available at: https://echa.europa.eu/documents/10162/17235/information_requirements_r4_en.pdf/d6395ad2-1596-4708-ba86-0136686d205e?t=1323782558175

EUROPEAN COMMISSION (2002). Guidance document on aquatic ecotoxicology in the context of the Directive 91/414/EEC. Working Document. Brussels, Belgium.

EUROPEAN COMMISSION (2011). Common implementation strategy for the Water Framework Directive (2000/60/EC). Guidance document No. 27. Technical guidance for deriving environmental quality standards. Available at: https://circabc.europa.eu/sd/a/0cc3581b-5f65-4b6f-91c6-433a1e947838/TGD-environmental quality standard%20CIS-WFD%2027%20EC%202011.pdf

FORD, A. T., ÅGERSTRAND, M., BROOKS, B. W., ALLEN, J., BERTRAM, M. G., BRODIN, T., DANG, Z., DUQUESNE, S., SAHM, R., HOFFMANN, F., HOLLERT, H., JACOB, S., KLÜVER, N., LAZORCHAK, J., LEDESMA, M., *ET AL*. (2021). The role of behavioral ecotoxicology in environmental protection. *Environmental Science & Technology* **55**, 5620–5628.

FRIARD, O. & GAMBA, M. (2016). BORIS: a free, versatile open-source event-logging software for video/audio coding and live observations. *Methods in Ecology and Evolution* **7**, 1325–1330.

FROST, A. J., WINROW-GIFFEN, A., ASHLEY, P. J. & SNEDDON, L. U. (2007). Plasticity in animal personality traits: does prior experience alter the degree of boldness? *Proceedings of the Royal Society B: Biological Sciences* **274**, 333–339.

FURSDON, J. B., MARTIN, J. M., BERTRAM, M. G., LEHTONEN, T. K. & WONG, B. B. M. (2019). The pharmaceutical pollutant fluoxetine alters reproductive behaviour in a fish independent of predation risk. *Science of the Total Environment* **650**, 642–652.

GAWORECKI, K. M. & KLAINE, S. J. (2008). Behavioral and biochemical responses of hybrid striped bass during and after fluoxetine exposure. *Aquatic Toxicology* **88**, 207–213.

GOMES, D. G. E., POTTIER, P., CRYSTAL-ORNELAS, R., HUDGINS, E., FOROUGHIRAD, V., SÁNCHEZ-REYES, L. L., TURBA, R., MARTINEZ, P. A., MOREAU, D., BERTRAM, M. G., SMOUT, C. A. & GAYNOR, K. M. (2022). Why don't we share data and code? Perceived barriers and benefits to public archiving practices. *Proceedings of the Royal Society B: Biological Sciences* **289**, 20221113.

GORE, A. C., HOLLEY, A. M. & CREWS, D. (2018). Mate choice, sexual selection, and endocrine-disrupting chemicals. *Hormones and Behavior* **101**, 3–12.

GREEN, J. W., SPRINGER T. A. & HOLBECH, H. (2018). *Statistical analysis of ecotoxicity studies*. Hoboken, NJ, USA: John Wiley & Sons.

GUEVARA-FIORE, P., SKINNER, A. & WATT, P. J. (2009). Do male guppies distinguish virgin females from recently mated ones? *Animal Behaviour* **77**, 425–431.

HAMILTON, P. B., ROLSHAUSEN, G., UREN WEBSTER, T. M. & TYLER, C. R. (2017). Adaptive capabilities and fitness consequences associated with pollution exposure in fish. *Philosophical Transactions of the Royal Society B: Biological Sciences* **372**, 20160042.

HÄRKÖNEN, L., HYVÄRINEN, P., NIEMELÄ, P. T. & VAINIKKA, A. (2016). Behavioural variation in Eurasian perch populations with respect to relative catchability. *Acta Ethologica* **19**, 21–31.

HARRIS, C. A., SCOTT, A. P., JOHNSON, A. C., PANTER, G. H., SHEAHAN, D., ROBERTS, M. & SUMPTER, J. P. (2014). Principles of sound ecotoxicology. *Environmental Science & Technology* **48**, 3100–3111.

HARTMANN, N. B., ÅGERSTRAND, M., LÜTZHØFT, H. C. H. & BAUN, A. (2017). NanoCRED: A transparent framework to assess the regulatory adequacy of ecotoxicity data for nanomaterials–relevance and reliability revisited. *NanoImpact* **6**, 81–89.

HELLSTRÖM, G., KLAMINDER, J., FINN, F., PERSSON, L., ALANÄRÄ, A., JONSSON, M., FICK, J. & BRODIN, T. (2016). GABAergic anxiolytic drug in water increases migration behaviour in salmon. *Nature Communications* **7**, 13460.

HELLSTRÖM, G., LENNOX, R. J., BERTRAM, M. G. & BRODIN, T. (2022). Acoustic telemetry. *Current Biology* **32**, R863–R865.

HENRY, J., BAI, Y., WILLIAMS, D., LOGOZZO, A., FORD, A.T. & WLODKOWIC, D. (2022). Impact of test chamber design on spontaneous behavioral responses of model crustacean zooplankton *Artemia franciscana*. *Lab Animal* **51**, 81–88.

HENRY, J., RODRIGUEZ, A. & WLODKOWIC, D. (2019). Impact of digital video analytics on accuracy of chemobehavioural phenotyping in aquatic toxicology. *Peerj* **7**, e7367.

HENRY, J. & WLODKOWIC, D. (2020). High-throughput animal tracking in chemobehavioral phenotyping: current limitations and future perspectives. *Behavioural Processes* **180**, 104226.

HOFFMANN, F. & KLOAS, W. (2010). An environmentally relevant endocrine-disrupting antiandrogen, vinclozolin, affects calling behavior of male *Xenopus laevis*. *Hormones and Behavior* **58**, 653–659.

HOLMAN, L., HEAD, M. L., LANFEAR, R. & JENNIONS, M. D. (2015). Evidence of experimental bias in the life sciences: why we need blind data recording. *PLoS Biology* **13**, e1002190.

HURLBERT, S. H. (1984). Pseudoreplication and the design of ecological field experiments. *Ecological Monographs* **54**, 187–211.

HUTCHINSON, T. H., ANKLEY, G. T., SEGNER, H. & TYLER, C. R. (2006). Screening and testing for endocrine disruption in fish—Biomarkers as "signposts," not "traffic lights," in risk assessment. *Environmental Health Perspectives* **114**, 106–114.

JENNIONS, M. D. & MØLLER, A. P. (2003). A survey of the statistical power of research in behavioral ecology and animal behavior. *Behavioral Ecology* **14**, 438–445.

JOHANSSON, F., LEDERER, B. & LIND, M. I. (2010). Trait performance correlations across life stages under environmental stress conditions in the common frog, *Rana temporaria*. *PLoS One* **5**, e11680.

JONES, N. A. R., WEBSTER, M. M. & SALVANES, A. G. V. (2021). Physical enrichment research for captive fish: time to focus on the DETAILS. *Journal of Fish Biology* **99**, 704–725.

JUTFELT, F., SUNDIN, J., RABY, G. D., KRÅNG, A. & CLARK, T. D. (2016). Two-current choice flumes for testing avoidance and preference in aquatic animals. *Methods in Ecology and Evolution* **8**, 379–390.

KARDISH, M. R., MUELLER, U. G., AMADOR-VARGAS, S., DIETRICH, E. I., MA, R., BARRETT, B. & FANG, C. C. (2015). Blind trust in unblinded observation in ecology, evolution, and behavior. *Frontiers in Ecology and Evolution* **3**, 51.

KLIMISCH, H. -J., ANDREAE, M. & TILLMANN, U. (1997). A systematic approach for evaluating the quality of experimental toxicological and ecotoxicological data. *Regulatory Toxicology and Pharmacology* **25**, 1–5.

KLIOT, A. & GHANIM, M. (2012). Fitness costs associated with insecticide resistance. *Pest Management Science* **68**, 1431–1437.

KOHDA, M., SOGAMA, S., JORDAN, A. L., KUBO, N., AWATA, S., SATOH, S., KOBAYASHI, T., FUJITA, A. & BSHARY, R. (2022). Further evidence for the capacity of mirror self-recognition in cleaner fish and the significance of ecologically relevant marks. *PLOS Biology* **17**, e3000021.

KOHLER, S. A., PARKER, M. O. & FORD, A. T. (2018). Shape and size of the arenas affect amphipod behaviours: implications for ecotoxicology. *PeerJ* **6**, e5271.

LAGESSON, A., BRODIN, T., FAHLMAN, J., FICK, J., JONSSON, M., PERSSON, J., BYSTRÖM, P. & KLAMINDER, J. (2018). No evidence of increased growth or mortality in fish exposed to oxazepam in semi-natural ecosystems. *Science of the Total Environment* **615**, 608–614.

LAGESSON, A., SAARISTO, M., BRODIN, T., FICK, J., KLAMINDER, J., MARTIN, J. M. & WONG, B. B. M. (2019). Fish on steroids: temperature-dependent effects of 17β-trenbolone on predator escape, boldness, and exploratory behaviors. *Environmental Pollution* **245**, 243–252.

LEVIN, E. D., BENCAN, Z. & CERUTTI, D. T. (2007). Anxiolytic effects of nicotine in zebrafish. *Physiology & Behavior* **90**, 54–58.

LIMA, S. L. (1998). Stress and decision-making under the risk of predation: recent developments from behavioral, reproductive, and ecological perspectives. *Advances in the Study of Behaviour* **27**, 215–290.

LIU, C. M. & KANOSKI, S. E. (2018). Homeostatic and non-homeostatic controls of feeding behavior: distinct vs. common neural systems. *Physiology & Behavior* **193**, 223–231.

MAKARAS, T., STANKEVIČIŪTĖ, M., ŠIDAGYTĖ-COPILAS, E., VIRBICKAS, T. & RAZUMIENĖ, J. (2021). Acclimation effect on fish behavioural characteristics: determination of appropriate acclimation period for different species. *Journal of Fish Biology* **99**, 502–512.

MARSHALL, D. J. (2024). Principles of experimental design for ecology and evolution. *Ecology Letters*, **27**, e14400.

MARTIN, J. M., BERTRAM, M. G., SAARISTO, M., FURSDON, J. B., HANNINGTON, S. L., BROOKS, B. W., BURKET, S. R., MOLE, R. A., DEAL, N. D. S. & WONG, B. B. M. (2019*a*). Antidepressants in surface waters: fluoxetine influences mosquitofish anxiety-related behavior at environmentally relevant levels. *Environmental Science & Technology* **53**, 6035–6043.

MARTIN, J. M. & MCCALLUM, E. S. (2021). Incorporating animal social context in ecotoxicology: can a single individual tell the collective story? *Environmental Science & Technology* **55**, 10908–10910.

MARTIN, J. M., SAARISTO, M., BERTRAM, M. G., LEWIS, P. J., COGGAN, T. L., CLARKE, B. O. & WONG, B. B. M. (2017). The psychoactive pollutant fluoxetine compromises antipredator behaviour in fish. *Environmental Pollution* **222**, 592–599.

MARTIN, J. M., SAARISTO, M., HUNG, T., BERTRAM, M. G., NAGARAJAN-RADHA, V., DOWLING, D. K. & WONG, B. B. M. (2019*b*). Field-realistic antidepressant exposure disrupts group foraging dynamics in mosquitofish. *Biology Letters* **5**, 20190615.

MASON, R. T., MARTIN, J. M., TAN, H., BRAND, J. A., BERTRAM, M. G., TINGLEY, R., TODD-WECKMANN, A. & WONG, B. B. M. (2021). Context is key: social environment mediates the impacts of a psychoactive pollutant on shoaling behavior in fish. *Environmental Science & Technology* **55**, 13024−13032.

McNamara, J. M. & Houston, A.I. (1986). The common currency for behavioral decisions. *The American Naturalist* **127**, 358–378.

McPhail, D. J. & Paragamian, V. L., (2000). Burbot biology and life history, in: McPhail, D.J., Willis, D.W. (Eds.), Burbot: Biology, Ecology, and Management, Fisheries Management Section. American Fisheries Society, pp. 11–23.

Melvin, S. D. (2017). Effect of antidepressants on circadian rhythms in fish: insights and implications regarding the design of behavioural toxicity tests. *Aquatic Toxicology* **182**, 20–30.

Melvin, S. D., Petit, M. A., Duvignacq, M. C. & Sumpter, J. P. (2017). Towards improved behavioural testing in aquatic toxicology: acclimation and observation times are important factors when designing behavioural tests with fish. *Chemosphere* **180**, 430–436.

Melvin, S. D. & Wilson, S. P. (2013). The utility of behavioral studies for aquatic toxicology testing: a meta-analysis. *Chemosphere* **93**, 2217–2223.

Mensink, B. J. W. G., Smith, C. E. & Montforts M. H. M. M. (2008). Manual for summarizing and evaluating aspects of plant protection products. Report number 601712006/2010. RIVM, Bilthoven, The Netherlands.

Michelangeli, M., Martin, J. M., Pinter-Wollman, N., Ioannou, C. C., McCallum, E. S., Bertram, M. G. & Brodin, T. (2022). Predicting the impacts of chemical pollutants on animal groups. *Trends in Ecology & Evolution* **37**, 789–802.

Moermond, C. T. A., Kase, R., Korkaric, M. & Ågerstrand, M. (2016). CRED: criteria for reporting and evaluating ecotoxicity data. *Environmental Toxicology and Chemistry* **35**, 1297–1309.

Mohammed, A. (2013). Why are early life stages of aquatic organisms more sensitive to toxicants than adults? *New Insights into Toxicity and Drug Testing*, 49–62.

Morgan, R., Andreassen, A. H., Åsheim, E. R., Finnøen, M. H., Dresler, G., Brembu, T., Loh, A., Miest, J. J. & Jutfelt, F. (2022). Reduced physiological plasticity in a fish adapted to stable temperatures. *Proceedings of the National Academy of Sciences* **119**, e2201919119.

MUNDRY, R. (2010). Issues in information theory-based statistical inference—a commentary from a frequentist's perspective. *Behavioral Ecology and Sociobiology* **65**, 57–68.

NAKAGAWA, S. (2004). Farewell to Bonferroni: the problems of low statistical power and publication bias. *Behavioral Ecology* **6**, 1044–1045.

NÄSLUND, J. & JOHNSSON, J. I. (2014). Environmental enrichment for fish in captive environments: effects of physical structures and substrates. *Fish and Fisheries* **17**, 1–30.

NATHAN, R., MONK, C., ARLINGHAUS, R., ADAM, T., ALÓS, J., ASSAF, M., BAKTOFT, H., BEARDSWORTH, C. E., BERTRAM, M. G., BIJLEVELD, A., BRODIN, T., BROOKS, J., CAMPOS-CANDELA, A., COOKE, S. J., GJELLAND, K. J., *ET AL*. (2022). Big-data approaches lead to an increased understanding of the ecology of animal movement. *Science* **375**, eabg1780.

NICKERSON, R. S. (1998). Confirmation bias: a ubiquitous phenomenon in many guises. *Review of General Psychology* **2**, 175–220.

ORFORD, J. T., TAN, H., TINGLEY, R., ALTON, L. A., WONG, B. B. M. & MARTIN, J. M. (2023). Bigger and bolder: widespread agricultural pollutant 17β-trenbolone increases growth and alters behaviour in tadpoles (*Litoria ewingii*). *Aquatic Toxicology* **260**, 106577.

ORGANISATION FOR ECONOMIC CO-OPERATION AND DEVELOPMENT (OECD) (2006). Current approaches in the statistical analysis of ecotoxicology data: a guidance to application. OECD Series on Testing and Assessment, no. 54. Paris, France. Available at: https://www.oecd-ilibrary.org/environment/current-approaches-in-the-statistical-analysis-of-ecotoxicity-data_9789264085275-en

ORGANISATION FOR ECONOMIC CO-OPERATION AND DEVELOPMENT (OECD) (2012). Information on OECD work related to endocrine disrupters. Paris, France. Available at: http://www.oecd.org/env/ehs/testing/50067203.pdf

PARKER, T. H., FORSTMEIER, W., KORICHEVA, J., FIDLER, F., HADFIELD, J. D., CHEE, Y. E., KELLY, C. D., GUREVITCH, J. & NAKAGAWA, S. (2016). Transparency in ecology and evolution: real problems, real solutions. *Trends in Ecology & Evolution* **31**, 711–719.

PAULL, G. C., VAN LOOK, K. J. W., SANTOS, E. M., FILBY, A. L., GRAY, D. M., NASH, J. P. & TYLER, C. R. (2008). Variability in measures of reproductive success in laboratory-kept

colonies of zebrafish and implications for studies addressing population-level effects of environmental chemicals. *Aquatic Toxicology* **87**, 115–126.

PETERSON, E. K., BUCHWALTER, D. B., KERBY, J. L., LEFAUVE, M. K., VARIAN-RAMOS, C. W. & SWADDLE, J. P. (2017). Integrative behavioral ecotoxicology: bringing together fields to establish new insight to behavioral ecology, toxicology, and conservation. *Current Zoology* **63**, 185–194.

POLVERINO, G., MARTIN, J. M., BERTRAM, M. G., ROMAN, V. R., TAN, H. BRAND, J. A., MASON, R. T. & WONG, B. B. M. (2021). Psychoactive pollution suppresses individual differences in fish behaviour. *Proceedings of the Royal Society B: Biological Sciences* **288**, 20202294.

QUINN, G. P. & KEOUGH, M. J. (2002). *Experimental designs and data analysis for biologists*. Cambridge, UK: Cambridge University Press.

RADERSCHALL, C. A., MAGRATH, R. D. & HEMMI, J. M. (2011). Habituation under natural conditions: model predators are distinguished by approach direction. *Journal of Experimental Biology* **214**, 4209–4216.

REYHANIAN, N., VOLKOVA, K., HALLGREN, S., BOLLNER, T., OLSSON, P. E., OLSÉN, H. & HÄLLSTRÖM, I. P. (2011). 17α-Ethinyl estradiol affects anxiety and shoaling behavior in adult male zebra fish (*Danio rerio*). *Aquatic Toxicology* **105**, 41–48.

RICHARDSON, J. & ZUK, M. (2023). Unlike a virgin: a meta-analytical review of female mating status in studies of female mate choice. *Behavioral Ecology* **34**, 165–182.

RODRIGUEZ, A., ZHANG, H., KLAMINDER, J., BRODIN, T., ANDERSSON, P. L. & ANDERSSON, M. (2018). ToxTrac: a fast and robust software for tracking organisms. *Methods in Ecology and Evolution* **9**, 460–464.

ROYLE, N. J., SMISETH, P. T. & KÖLLIKER, M. (Eds.). (2012). The evolution of parental care. Oxford University Press.

SAARISTO, M., BRODIN, T., BALSHINE, S., BERTRAM, M. G., BROOKS, B.W., EHLMAN, S. M., MCCALLUM, E. S., SIH, A., SUNDIN, J., WONG, B. B. M. & ARNOLD, K. E. (2018). Direct and indirect effects of chemical contaminants on the behaviour, ecology and evolution of wildlife. *Proceedings of the Royal Society B: Biological Sciences* **285**, 20181297.

SAARISTO, M., LAGESSON, A., BERTRAM, M.G., FICK, J., KLAMINDER, J., JOHNSTONE, C.P., WONG, B.B.M. & BRODIN, T. (2019). Behavioural effects of psychoactive pharmaceutical exposure on European perch (*Perca fluviatilis*) in a multi-stressor environment. *Science of the Total Environment* **655**, 1311–1320.

SCHLIGLER, J., CORTESE, D., BELDADE, R., SWEARER, S. E. & MILLS, S. C. (2021). Long-term exposure to artificial light at night in the wild decreases survival and growth of a coral reef fish. *Proceedings of the Royal Society B: Biological Sciences* **288**, 20210454.

SHAW, A. K. (2020). Causes and consequences of individual variation in animal movement. *Movement Ecology* **8**, 12.

SIH, A., BOLNICK, D. I., LUTTBEG, B., ORROCK, J. L., PEACOR, S. D., PINTOR, L. M., PREISSER, E., REHAGE, J. S. & VONESH, J. R. (2010). Predator–prey naïveté, antipredator behavior, and the ecology of predator invasions. *Oikos* **119**, 610–621.

SIMÃO, F. C. P., MARTINEZ-JERONIMO, F., BLASCO, V., MORENO, F., PORTA, J. M., PESTANA, J. L. T., SOARES, A., RALDUA, D. & BARATA, C. (2019). Using a new high-throughput video-tracking platform to assess behavioural changes in *Daphnia magna* exposed to neuro-active drugs. *Science of the Total Environment* **662**, 160–167.

SÖFFKER, M. & TYLER, C. R. (2012). Endocrine disrupting chemicals and sexual behaviors in fish–a critical review on effects and possible consequences. *Critical Reviews in Toxicology* **42**, 653–668.

SUNDIN, J., JUTFELT, F., THORLACIUS, M., FICK, J. & BRODIN, T. (2019). Behavioural alterations induced by the anxiolytic pollutant oxazepam are reversible after depuration in a freshwater fish. *Science of the Total Environment* **665**, 390–399.

SUURVÄLI, J., WHITELEY, A. R., ZHEN, Y., GHARBI, K., LEPTIN, M. & WIEHE, T. (2020). The laboratory domestication of zebrafish: from diverse populations to inbred substrains. *Molecular Biology and Evolution* **37**, 1056–1069.

TAI, J. K. A. C., HORZMANN, K. A., JENKINS, T. L., AKORO, I. N., STRADTMAN, S., ARYAL, U. K. & FREEMAN, J. L. (2023). Adverse developmental impacts in progeny of zebrafish exposed to the agricultural herbicide atrazine during embryogenesis. *Environment International* **180**, 108213.

TAN, H., POLVERINO, G., MARTIN, J. M., BERTRAM, M. G., WILES, S. C., PALACIOS, M. M., BYWATER, C. L., WHITE, C. R. & WONG, B. B. M. (2020). Chronic exposure to a pervasive pharmaceutical pollutant erodes among-individual phenotypic variation in a fish. *Environmental Pollution* **263**, 114450.

TANOUE, R., MARGIOTTA-CASALUCI, L., HUERTA, B., RUNNALLS, T. J., EGUCHI, A., NOMIYAMA, K., KUNISUE, T., TABABE, S. & SUMPTER, J. P. (2019). Protecting the environment from psychoactive drugs: problems for regulators illustrated by the possible effects of tramadol on fish behaviour. *Science of the Total Environment* **664**, 915–926.

THORÉ, E. S. J., AULSEBROOK, A. E., BRAND, J. A., ALMEIDA, R. A., BRODIN, T. & BERTRAM, M. G. (2023*a*). Time is of the essence: the importance of considering biological rhythms in an increasingly polluted world. *PLoS Biology* **22**, e3002478.

THORÉ, E. S. J., BRENDONCK, L. & PINCEEL, T. (2020). Conspecific density and environmental complexity impact behaviour of turquoise killifish (*Nothobranchius furzeri*). *Journal of Fish Biology* **97**, 1448–1461.

THORÉ, E. S. J., BRENDONCK, L. & PINCEEL, T. (2021*a*). Natural daily patterns in fish behaviour may confound results of ecotoxicological testing. *Environmental Pollution* **276**, 116738.

THORÉ, E. S. J., BRENDONCK, L. & PINCEEL, T. (2021*b*). Neurochemical exposure disrupts sex-specific trade-offs between body length and behaviour in a freshwater crustacean. *Aquatic Toxicology* **237**, 105877.

THORÉ, E. S. J., GRÉGOIR, A. F., ADRIAENSSENS, B., PHILIPPE, C., STOKS, R., BRENDONCK, L. & PINCEEL, T. (2019*a*). Population-, sex- and individual level divergence in life-history and activity patterns in an annual killifish. *PeerJ* **7**, e7177.

THORÉ, E. S. J., PHILIPPE, C., BRENDONCK, L. & PINCEEL, T. (2021*c*). Towards improved fish tests in ecotoxicology-efficient chronic and multi-generational testing with the killifish *Nothobranchius furzeri*. *Chemosphere* **273**, 129697.

THORÉ, E. S. J., STEENAERTS, L. PHILIPPE, C., GRÉGOIR, A. F., BRENDONCK, L. & PINCEEL, T. (2019*b*). Improving the reliability and ecological validity of pharmaceutical risk assessment: turquoise killifish (*Nothobranchius furzeri*) as a model in behavioral ecotoxicology. *Environmental Toxicology and Chemistry* **38**, 262–270.

THORÉ, E. S. J., VANDEN BERGHEN, B., BRENDONCK, L. & PINCEEL, T. (2023*b*). Long-term exposure to a pharmaceutical pollutant affects geotaxic behaviour in the adult but not juvenile life stage of killifish. *Science of the Total Environment* **876**, 162746.

VERHEYEN, J., DELNAT, V. & STOKS, R. (2019). Increased daily temperature fluctuations overrule the ability of gradual thermal evolution to offset the increased pesticide toxicity under global warming. *Environmental Science & Technology* **53**, 4600–4608.

VOSSEN, L. E., BRUNBERG, R., RÅDÉN, P., WINBERG, S. & ROMAN, E. (2022). Sex-specific effects of acute ethanol exposure on locomotory activity and exploratory behavior in adult zebrafish (*Danio rerio*). *Frontiers in Pharmacology* **13**, 853936.

VOSSEN, L. E., CERVENY, D., SARMA, O. S., THÖRNQVIST, P. O., JUTFELT, F., FICK, J., BRODIN, T. & WINBERG, S. (2020). Low concentrations of the benzodiazepine drug oxazepam induce anxiolytic effects in wild-caught but not in laboratory zebrafish. *Science of the Total Environment* **703**, 134701.

WARD, A. J. W., KENT, M. I. & WEBSTER, M. M. (2020). Social recognition and social attraction in group-living fishes. *Frontiers in Ecology and Evolution* **8**, 15.

WONG, B. B. M. & CANDOLIN, U. (2015). Behavioral responses to changing environments. *Behavioral Ecology* **26**, 665–673.
